# An Enzymatic Platform for Late‐Stage (Radio)isotope Labelling of Oligonucleotides With Methyltransferases

**DOI:** 10.1002/anie.8706793

**Published:** 2026-07-27

**Authors:** Christopher R. B. Swanson, Ulrich Kauhl, Martin R. Edelmann, Florian P. Seebeck

**Affiliations:** ^1^ Department of Chemistry University of Basel Basel Switzerland; ^2^ Therapeutic Modalities, Isotope Synthesis Roche Innovation Center Basel Basel Switzerland

**Keywords:** biocatalysis, enzyme catalysis, methyltransferase, oligonucleotides, radiochemistry

## Abstract

The development of therapeutic nucleic acids has become a cornerstone of modern drug discovery. To support the clinical translation of these modalities, radiolabelled analogues are vital for absorption, distribution, metabolism and excretion (ADME) studies, particularly for quantitative whole‐body autoradiography (QWBA). Access to such radio probes is limited because established methods for the preparation of ^3^H‐labelled oligonucleotides are characterised by high costs, extensive lead times, low specific activity and the generation of substantial radioactive waste. To address these limitations, we developed a biocatalytic platform for late‐stage ^3^H‐radiolabelling of oligonucleotides using methyltransferases, reducing precursor costs and radioactive waste by orders of magnitude. We demonstrate that DNA‐cytosine‐5‐methyltransferases can methylate oligonucleotides with high regioselectivity at a preparative scale, yielding stable‐ and radioisotope‐labelled (^2^H and ^3^H) samples with an unprecedented molar activity up to 3 TBq mmol^−1^ (81 Ci mmol^−1^). In view of the precision and efficiency of methyltransferase‐mediated radiolabelling, we anticipate that similar approaches will facilitate QWBA studies with other drug modalities.

## Graphical abstract

State‐of‐the‐art synthesis of radiolabelled oligonucleotides involves multi‐step synthesis with high costs, extended timelines and significant radioactive waste generation. Herein, a dual‐methyltransferase platform enables late‐stage, site‐specific labelling, overcoming these limitations through reduced costs, faster turnaround and minimal radioactive waste.

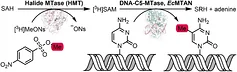

## 1 Introduction

**FIGURE 1:**
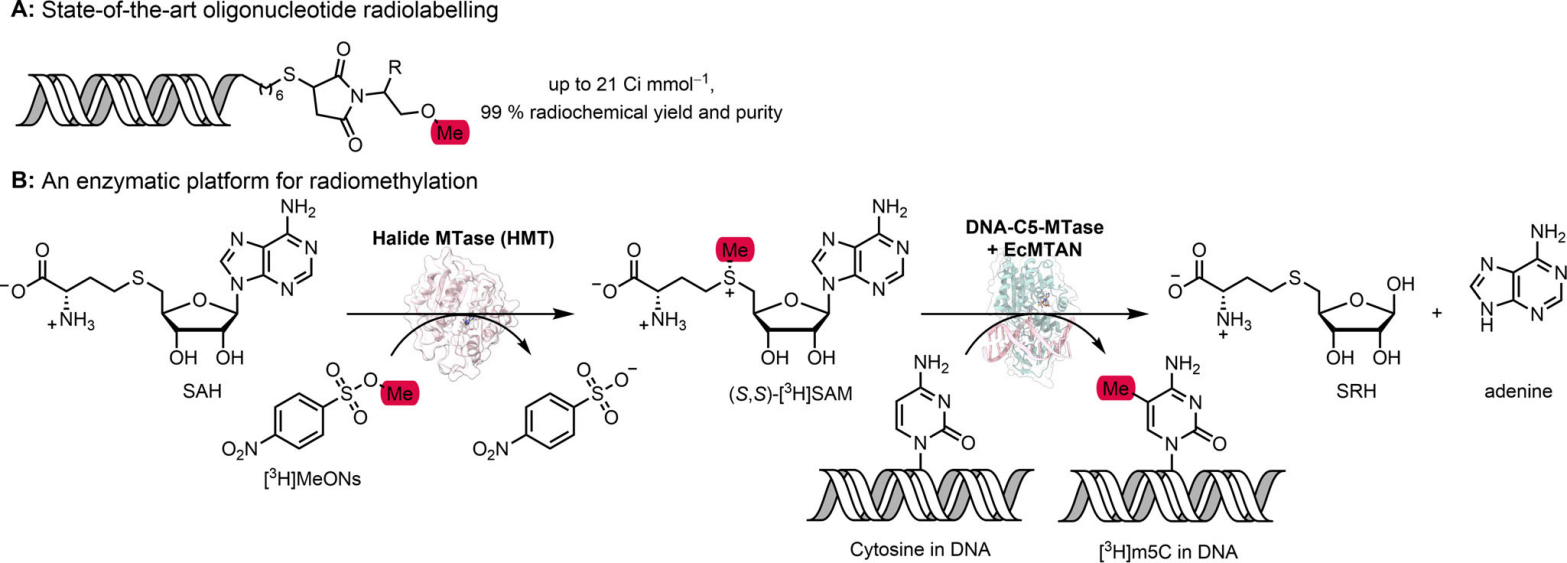
(A) The state‐of‐the‐art method for oligonucleotide radiolabelling using a thiol conjugated oligonucleotide and radiolabelled maleimide substrate [28]. (B) An enzymatic concept for selective isotope labelling of oligonucleotides via methyltransferases. **SAM**: *S*‐adenosyl‐l‐methionine; **SAH**: *S*‐adenosyl‐l‐homocysteine; **SRH**: *S*‐ribosyl‐l‐homocysteine; **HMT**: halide methyltransferase; **DNA‐C‐MTase**: DNA‐cytosine‐5‐methyltransferase; **MTAN**: 5′‐methylthioadenosine/ *S*‐adenosyl‐l‐homocysteine nucleosidase.

Therapeutic oligonucleotides are a rapidly expanding class of therapeutics set to revolutionise the treatment of previously ‘undruggable’ diseases [1, 2]. As the range of their therapeutic application expands and the patient populations into which they are administered grow, so does the need for new methods for the synthesis and preclinical tracking of these molecules [1, 2, 3]. In particular, radiolabelled oligonucleotides are required for absorption, distribution, metabolism and excretion (ADME) studies, particularly for quantitative whole‐body autoradiography (QWBA) [3, 4, 5]. Given the molecular complexity of oligonucleotides, the preparation of radioactive derivatives presents a formidable challenge. Current state‐of‐the‐art chemical synthesis via phosphoramidite chemistry is hampered by the requirement for complex radioactive precursors, dedicated solid‐phase synthesis instrumentation and a comparatively low labelling efficiency [3, 6, 7]. Using up to a 10‐fold excess of tritium‐labelled phosphoramidite precursor, these methods only furnish oligonucleotides with molar activities of 2.2 to 37 GBq mmol^−1^ (0.06 to 1 Ci mmol^−1^) [3, 6, 7, 8]. Late‐stage labelling by bioconjugation with radioactive auxiliaries produces compounds that are structurally distinct from the therapeutic compound (Figure 1A), and tritium exchange of nucleobase protons lacks selectivity, generating heterogeneous mixtures of oligonucleotides with labile radiotracers, alongside substantial amounts of radioactively contaminated waste [3, 6, 9].

Since oligonucleotides are natural compounds, biocatalysis may provide solutions to the challenge of late‐stage radiolabelling [2, 10, 11, 12, 13, 14]. Indeed, the introduction of radioactive phosphorus (^32^P or ^33^P) to oligonucleotides using [γ‐^32^P] or [γ‐^33^P]ATP and polynucleotide kinase has been a central method in molecular biology for almost a century [15, 16, 17, 18]. However, these radioisotopes have short half‐lives (14 and 25 days, respectively) and therefore find limited application in in vivo preclinical studies. 5′‐Thiophosphorylation with [γ‐^35^S]ATP and a polynucleotide kinase provides a radiolabel with a useful half‐life (*t*_1_/_2_ = 87 days), but the terminal thiophosphate monoester is vulnerable to phosphatase‐mediated cleavage in vivo [17, 19, 20, 21].

An enzymatic labelling strategy based on DNA‐methyltransferases offers a promising alternative. These enzymes transfer methyl groups to specific nucleobases *via* stable C─C or C─N bonds [22, 23], enabling DNA to be decorated with tritium (^3^H) or carbon‐14 (^14^C) [6, 24]. However, this approach relies on a complex radioactive methyl donor: [^3^H]*S*‐adenosyl‐l‐methionine ([^3^H]SAM). Enzyme‐catalysed synthesis of [^3^H]SAM from ATP or 5′‐chloroadenosine has been reported and successfully applied to radiolabelling of both DNA and RNA (Figure S1) [24, 25]. Nevertheless, these methods still depend on [^3^H]l‐methionine as an upstream, specialised radiochemical reagent [25].

In this report, we present a streamlined method for radiolabelling of oligonucleotides. First, we introduce halide methyltransferases (HMTs) as biocatalysts for preparing [^3^H]SAM from tritiated methyl nosylate ([^3^H]MeONs) as the methyl donor (Figure 1B) [26, 27]. This approach is particularly advantageous because [^3^H]MeONs is a well‐established and commercially available reagent for chemical radiomethylation—and significantly cheaper than the specialised radiochemicals [^3^H]SAM and [^3^H]l‐methionine (see Figure S1 and Table S1) [28]. Second, we developed a high‐resolution electrospray ionisation mass spectrometry‐based (HR‐ESI‐MS) high‐throughput oligonucleotide methylation assay. This enabled the first systematic study of how well DNA‐methyltransferases tolerate backbone modifications characteristic of therapeutic oligonucleotides. Third, we demonstrate the direct use of DNA‐methyltransferases to prepare radiolabelled oligonucleotides with record‐high molar activity, achieved with less than a two‐fold excess of commercial [^3^H]MeONs.

## 2 Results and Discussion

To develop this methodology, we focused on DNA‐cytosine‐5‐methyltransferases (DNA‐C5‐MTases, EC 2.1.1.37, Figure 1B) as a model family of nucleic acid methyltransferases [22, 29]. Methylation of cytosine nucleobases to form 5‐methyl cytosine (m5C) enhances oligonucleotide target hybridisation and physiological stability [1, 30]. Therefore, m5C is a common, albeit not universal, modification in marketed therapeutic oligonucleotides [31, 32]. Amongst the 24 FDA‐approved therapeutic oligonucleotides, eight (33%) contain m5C. Notably, all eight are antisense oligonucleotides (ASOs), with Fomivirsen—the first FDA‐approved ASO—being the only one that lacks m5C. In contrast, no FDA‐approved siRNA or PMO‐ASO therapeutics contain m5C to date [31, 32]. Most importantly, substitution of cytosines by m5C appears to have become a routine design strategy for ASOs—supported by its inclusion in Tofersen, Olezarsen and Donidalorsen (approved in 2023, 2024 and 2025, respectively) [32].

In Nature, 5‐methyl cytosine serves as an epigenetic mark that affects fundamental biological processes, including development, differentiation, and genomic imprinting in eukaryotes, as well as restriction‐modification and mismatch repair systems in bacteria [33, 34, 35]. DNA‐C5‐MTases catalyse the SAM‐dependent C5 methylation of cytosines at specific recognition sites within DNA [35, 36]. Several DNA‐C5‐MTases have been characterised with respect to their structure, activity and biological roles [29, 33, 35, 37]. This enzyme family represents a large and, for the most part, unexplored class of biocatalysts with potential utility in late‐stage oligonucleotide radiolabelling and structural diversification. The family encompasses over 55,000 putative DNA‐C5‐MTase sequences (PFAM database, entry PF00145), yet only 505 confirmed DNA‐C5‐MTases are recorded in the REBASE database, which comprises 114 unique methylation sites, as illustrated in Figure S2 [3, 38, 39].

For the present study, we assembled a panel of six representative DNA‐C5‐MTases from five different subfamilies (see sequence similarity network in Figure S2), recognising six distinct methylation sites. These enzymes were M.HhaI (G**C**GC, methylated C in bold) [40], M.HaeIII (GG**C**C), M.MpeI (**C**G), M.HpaII (C**C**GG), M.Hpy99III (G**C**GC), EcDCM (M.EcoDCM, C**C**AGG and C**C**TGG). Recombinant DNA‐methyltransferases were produced in T7express *Escherichia coli* cells (NEB). The homogeneity of the recombinant proteins was assessed by SDS‐PAGE, and their identity was confirmed by HR‐ESI‐MS (Figures S3 and S4; Table S9). In addition, we produced and purified the auxiliary enzymes *Uma* (a MeONs utilising HMT) and *Ec*MTAN from ∆mtn *E. coli* cells and analysed them by SDS‐PAGE and HR‐ESI‐MS [26, 27].

To quantify the activity of DNA‐C5‐MTases towards synthetic DNA, we applied intact oligonucleotide HR‐ESI‐MS [41, 42, 43, 44, 45]. DNA methyltransferases are commonly assayed by enzymatic oligonucleotide digestion to nucleosides coupled with HPLC, gel‐based restriction enzyme cleavage/protection assays, or through the transfer of radioactive methyl groups [24]. In comparison, a mass spectrometry‐based approach offers increased throughput, generality across oligonucleotide modifications, and the direct observation and confirmation of the desired methylated oligonucleotide product [41, 42, 43, 44, 45].

To quantify the degree of methylation, we compared the signal intensities of the methylated and unmethylated oligonucleotides. Oligonucleotides are large, polyanionic species that primarily ionise via the charge residue model (CRM), and their ionisation efficiency is dominated by the phosphodiester backbone [46, 47]. As the backbone remains unchanged upon nucleobase methylation, the +14 Da mass shift is essentially invisible to the ionisation process for a large polyanion of this type. Indeed, quantifying the ratio between methylated and unmethylated nucleic acids by ESI‐MS is an established methodology [41, 48, 49]. The ratio of oligonucleotide signal intensities has even been shown to be quantitative when the phosphodiester backbone is perturbed by sequence truncation (*n*−1 impurity) [50]. Consistently, we found that the observed MS signal intensity ratio of methylated and unmethylated oligonucleotide **1** correlated well (*R* ^2^ ≥ 0.995) with the concentration ratio of analytes across multiple charge states (Figure S5).

**FIGURE 2:**
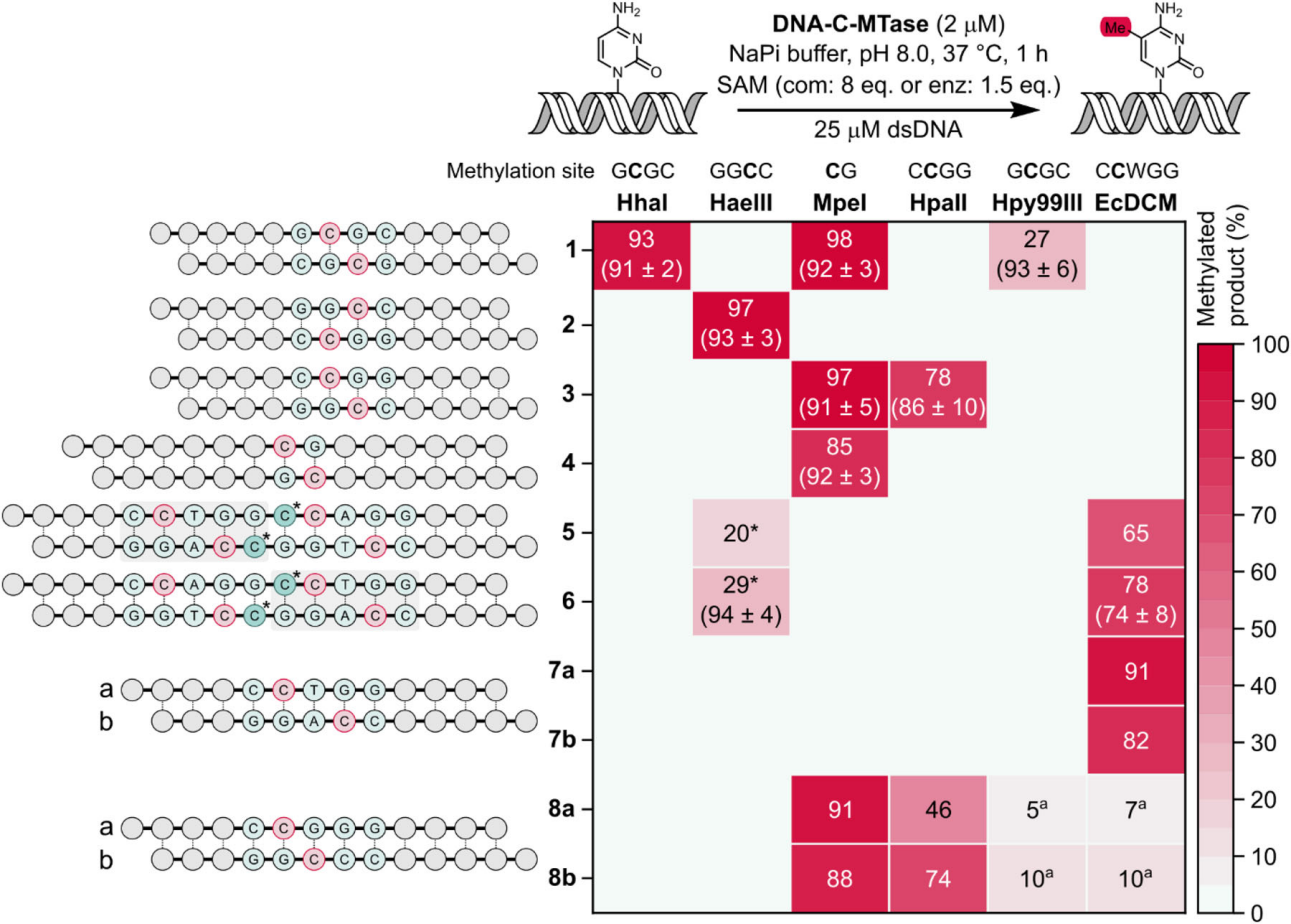
Screening of DNA‐cytosine‐5‐methyltransferases by HR‐ESI‐MS. The nucleotide to be methylated is highlighted in red (or, for substrates **5** and **6**, the HaeIII methylation site is highlighted in dark blue with an asterisk). Methylated product (%) was calculated by comparison of monoisotopic HRMS peaks for substrate and product, corrected for a no enzyme control reaction of each substrate. Data not in brackets: screening with 8 equiv commercial SAM; data in brackets (average methylation over three independent replicates ± standard deviation): reactions performed with enzymatically synthesised (*S*,*S*)‐SAM (1.5 equiv) and SAH removal (***Ec*MTAN**, 2 µM). In addition, all reactions contained: **DNA‐C5‐MTase** (2 µM), dsDNA substrate (**1–8**, 25 µM) in NaPi buffer (50 mM, pH 8.0) with DTT (1 mM), 37°C, 1 h. Blank cells: < 5% methylation. ^a^: methylation of a substrate that does not contain that enzyme's native methylation site. Full protocols, HRMS data plots and oligonucleotide sequences can be found in the Supporting Information (Figures S6–S15 and S20–S22).

In an initial enzyme panel screen, each 10 µL reaction contained 2 µM enzyme and 25 µM (double‐stranded basis) of one of eight double‐stranded DNA 13–17‐mers with either complementary or palindromic sequences (Figure 2). Commercial SAM was added as the methyl donor at 400–800 µM, corresponding to 8 equivalents relative to the methylatable cytosines in each reaction. Reactions were conducted in phosphate buffer (pH 8.0) and incubated at 37°C for 1 h. DNA was recovered by precipitation with ammonium acetate/isopropanol. Methylation efficiency was assessed by comparing ESI signal intensities of methylated and unmethylated oligonucleotides (Figures S6–S15). This analysis identified enzymes that efficiently methylated (≥78%) substrates **1–4** and **6–8**. Notably, the majority of enzyme–substrate combinations yielded less than 5% methylation (blank cells, Figure 2), highlighting the strict substrate specificity of DNA‐C5‐MTases, despite the abiotically high substrate concentration. Nevertheless, these data validate our ESI‐MS‐based assay for DNA‐methylation activity. Moreover, they indicate that near‐quantitative methylation (>90%) of synthetic oligonucleotides is achievable without extensive reaction optimisation (compounds **1–8**; Figures 2 and S6–S15).

In a second round of activity screening, substrates **1–4** and **6** (25 µM, double‐stranded basis) were incubated with the six DNA‐C5‐MTases (2 µM) in reactions supplemented with 2 µM of the SAH‐degrading enzyme EcMTAN, a nucleosidase included to prevent inhibitory accumulation of SAH [51]. Furthermore, we initiated the reaction by adding only 1.5 equivalents of (*S*,*S*)‐SAM (75 µM), which was generated by the HMT‐mediated methylation of SAH using MeONs as methyl donor. Despite its widespread use as a methyl donor for in vitro methylation reactions, commercial SAM presents notable pitfalls. Commercial SAM is produced by fermentation [52, 53, 54, 55, 56] and typically comprises a 2:1 to 1:1 mixture of *S*‐ and *R*‐epimers at the sulfonium centre, owing to the considerable rate of spontaneous epimerisation under physiological conditions (*k*_e_ = 1.8 × 10^−6^ s^−1^) [57, 58, 59]. Epimerisation reduces the efficiency of in vitro methylation reactions, as (*R*,*S*)‐SAM is catalytically inert yet can competitively inhibit methyltransferases [60, 61, 62, 63, 64]. These changes to the protocol—adding *Ec*MTAN and using (*S*,*S*)‐SAM improved oligonucleotide methylation efficiency (Figure 2, bracketed values). Notably, for two low‐yielding (<30%) enzyme–substrate combinations in the first screen (**1**:Hpy99III and **6**:HaeIII), methylation efficiency increased to >90%, despite using 5‐fold less SAM in the second screen.

**SCHEME 1:**
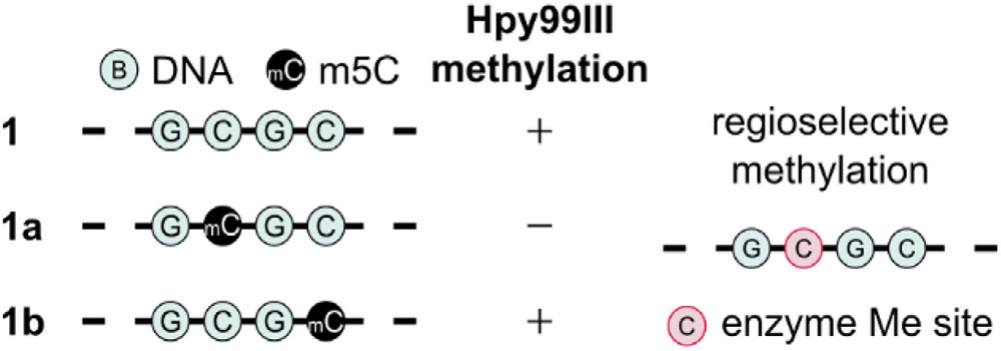
Establishment of M.Hpy99III methylation regioselectivity (GCGC) by reactions with premethylated substrate probes (based on substrate **1**). Reaction conditions were identical to those with enzymatically generated SAM in Figure 2. Full data can be found in the Supporting Information (Figure S23).

Of the enzymes tested in Figure 2, all but M.Hpy99III have been characterised with respect to their methylation site regioselectivity in vitro [22, 29]. To confirm the methylation specificity of M.Hpy99III, we used substrate probes with m5C pre‐installed at defined positions (Scheme 1 and Figure S23). Blocking of the internal cytosine of the GCGC motif (**1a**, Scheme 1) precluded oligonucleotide methylation by M.Hpy99III, whereas a substrate with the external m5C pre‐installed (**1b**, Scheme 1) was efficiently turned over by M.Hpy99III. These results support the annotation of M.Hpy99III as a G**C**GC specific methyltransferase and reaffirm the regioselectivity of DNA‐C5‐MTases under synthetically relevant reaction conditions.

**FIGURE 3:**
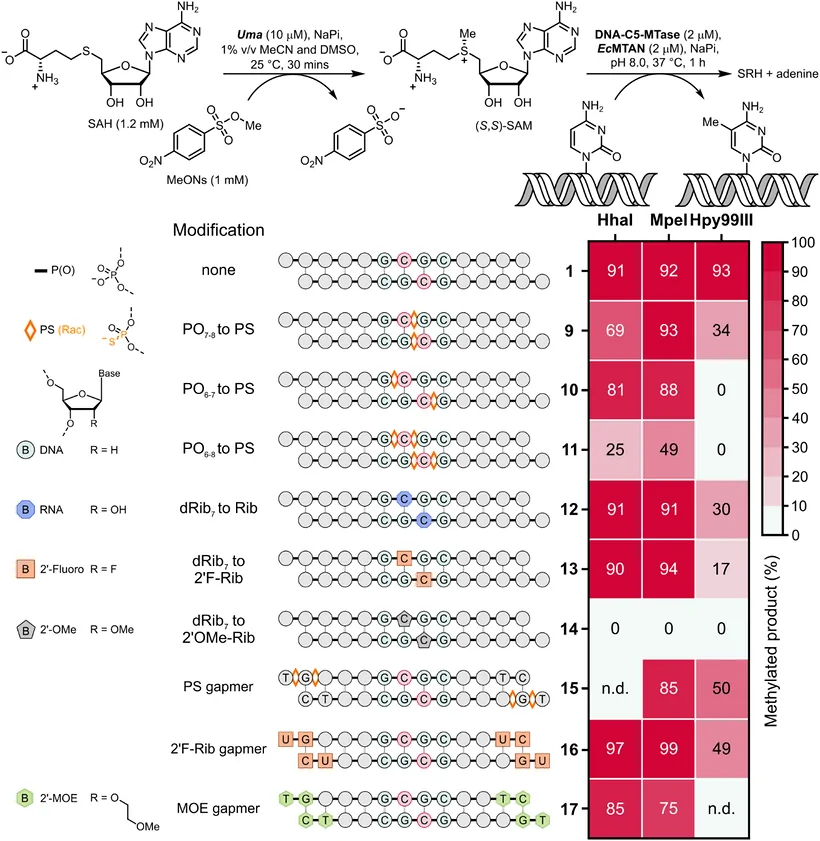
Methylation of oligonucleotides containing therapeutically relevant modifications by M.HhaI, M.MpeI and M.Hpy99III. Methylated product (%) was calculated as the mean of three independent replicates and the average experimental error is less than 10% (standard deviations can be found in Table S11). Reaction conditions were as follows: SAM formation: ***Uma*** (10 µM), SAH (1.2 mM) and MeONs (1 mM) in NaPi buffer (50 mM, pH 8.0) with 1% v/v each MeCN and DMSO, incubated at 25°C for 30 min and monitored by IEX‐HPLC‐UV. DNA methylation: **DNA‐C5‐MTase** (2 µM), ***Ec*MTAN** (2 µM), dsDNA substrate (**1–6**, 25 µM) and SAM (75 µM, or 1.5 equiv, from the crude SAM formation reaction) in NaPi buffer (50 mM, pH 8.0) with DTT (1 mM), incubated 37°C for 1 h before DNA precipitation and HR‐ESI‐MS analysis. Full data can be found in the Supporting Information (Figures S24–S32 and Table S11). n.d. = not determined.

With an efficient ESI‐based assay established, we explored the extent to which DNA‐C5‐MTases can methylate backbone‐modified nucleic acids. Therapeutic oligonucleotides contain extensively modified backbones to improve their in vivo stability, cell targeting specificity and hence pharmaceutical efficiency [1, 2, 30]. Key modifications include phosphorothioate linkages (replacing phosphodiester bonds) and nucleotides with 2′‐substituted riboses (Figure 3). These include 2′‐*O*‐methoxyethyl‐ribose (2′‐MOE), which is commonly incorporated into antisense oligonucleotides (ASOs), as well as 2′‐fluoro and 2′‐methoxy ribonucleotides (2′‐F and 2′‐OMe, respectively), which are standard building blocks of small interfering RNA (siRNA) [1]. As yet, no systematic evaluation of DNA methyltransferases against highly modified substrates containing therapeutically relevant modifications has been reported.

We approached this question in two ways. First, we designed a set of substrates bearing modifications at the target nucleotide itself. Since this nucleotide must undergo base flipping out of the DNA helix to engage the methyltransferase active site, non‐natural modifications at this position were expected to cause the greatest interference with catalysis [65, 66]. The second set of substrates bears modifications at the 5′‐ and 3′‐termini, representative of gapmer‐type antisense oligonucleotides [1, 67]. This substrate series assesses the impact of distal modifications on overall oligonucleotide binding and recognition. All oligonucleotides in this comparison contained the same sequence as substrate **1** and were tested as substrates for the ‘GCGC’ and ‘CG’‐methylating enzymes M.HhaI, M.MpeI and M.Hpy99III (Figure 3).

Oligonucleotides bearing one phosphorothioate linkage 5′ or 3′ to the target cytosine (**9** and **10**, Figure 3) were methylated efficiently (>69%, Figure 3) by both M.HhaI and M.MpeI. The substrate with 5′ and 3′‐phosphorothioate linkages (**11**), on the other hand, proved a lesser substrate (<50%), suggesting that additive effects reduce the efficiency of substrate recognition. M.Hpy99III was significantly less active against modified substrates, indicating that substrate recognition by this enzyme depends on more subtle features than M.HhaI and M.MpeI.

Amongst substrates with modifications at the 2′‐deoxyribose of the target nucleotide (**12–14**), M.MpeI and M.HhaI again showed remarkable tolerance for ribo‐cytidine (**12**) and 2′‐fluoro‐cytidine (**13**), with methylation efficiencies comparable to the native substrate **1**. In contrast, the 2′‐methoxy‐modified substrate (**14**) was not methylated by any enzyme. One explanation for this exclusion is that the methyltransferase active site cannot accommodate the extra methyl group on the substrate [66, 68, 69]. Indeed, the crystal structure of M.HhaI in complex with SAH and a DNA substrate (PDB: 5MHT) reveals direct van der Waals contacts between the backbone of residues 251–253 and the deoxyribose of the flipped cytosine. The tolerance for ribo‐cytidine in **12** may be rationalised by the ability of the 2′‐hydroxyl to form a hydrogen bond with the amide N–H of Ala253, in contrast to the *O*‐atom of a 2′‐methoxy group [66, 68, 69].

As anticipated, methyltransferase activity was significantly less affected by modifications in the flanking regions. M.MpeI efficiently methylated gapmers with multiple phosphorothioate linkages, 2′‐fluoro or 2′‐methoxyethyl (MOE) nucleotides at their termini (**15–17**) and M.HhaI was similarly efficient against 2′‐ribose modified substrates **15** and **16**. Hpy99III also tolerated terminally modified substrates **15** and **16** (ca. 50%) more efficiently than the internally modified substrates **10** and **13** (0%–34%), highlighting the higher tolerance of modifications distal to the methylation site. To our knowledge, this is the first report of DNA methyltransferases acting upon oligonucleotides with modified ribose backbones. This activity demonstrates the potential of DNA‐C5‐MTases for modification of therapeutically relevant ASO scaffolds, particularly for gapmer‐type compounds with bulky modifications confined to the 5′ or 3′ termini.

Besides backbone modifications, the strandedness of ASOs is another important structural feature that distinguishes therapeutic nucleic acids from natural DNA‐C5‐MTase substrates. ASOs are single stranded, whereas DNA‐C5‐MTases require a duplex DNA for their activity. To explore the methylation of a genuine ASO sequence, we selected the orphan drug Aganirsen (5′‐TATC **C** GGAGGGCT **C** GCCATGCTGCT‐3′) which contains two MpeI methylation sites. Surprisingly, when treated with M.MpeI (4 µM) and 1.5 equiv of (*S*,*S*)‐SAM, the single stranded substrate was efficiently singly methylated (>90%, no doubly methylated compound detected, Figure S34–S37) within 1 h without need of an annealed complimentary strand (Figures S34–S36). The exact methylation site was established using pre‐methylated substrate probes and was found to reside within a 6‐nucleotide palindromic sequence close to the 5′ terminus (5′‐…TC **C** GGA…‐3′). We hypothesise that this sequence forms a transient 6‐base pair homodimer duplex, enabling the site‐selective methylation of this site whilst the other site—which remains in a single‐stranded environment—is not methylated by the enzyme (Figure S34).

Finally, to apply methyltransferase biocatalysis to the production of radio probes, we addressed three additional aspects: optimising the production of [^3^H]SAM; scaling oligonucleotide methylation to quantities required for QWBA studies; and quantifying the overall radiolabelling efficiency across eight enzyme–substrate combinations as a proof of concept.

MeONs is unstable in neutral buffer (*t*½ = 3 h), leading to significant loss of activated methyl groups to the formation of methanol [27]. In radiolabelling applications, this not only wastes specific activity but also generates volatile radioactivity. To minimise this problem, we increased the concentration of HMT to ensure complete methyl transfer from the limiting MeONs (1 mM) to SAH (1.2 mM). Under these conditions, over 95% of methyl groups are transferred to SAH.

**FIGURE 4:**
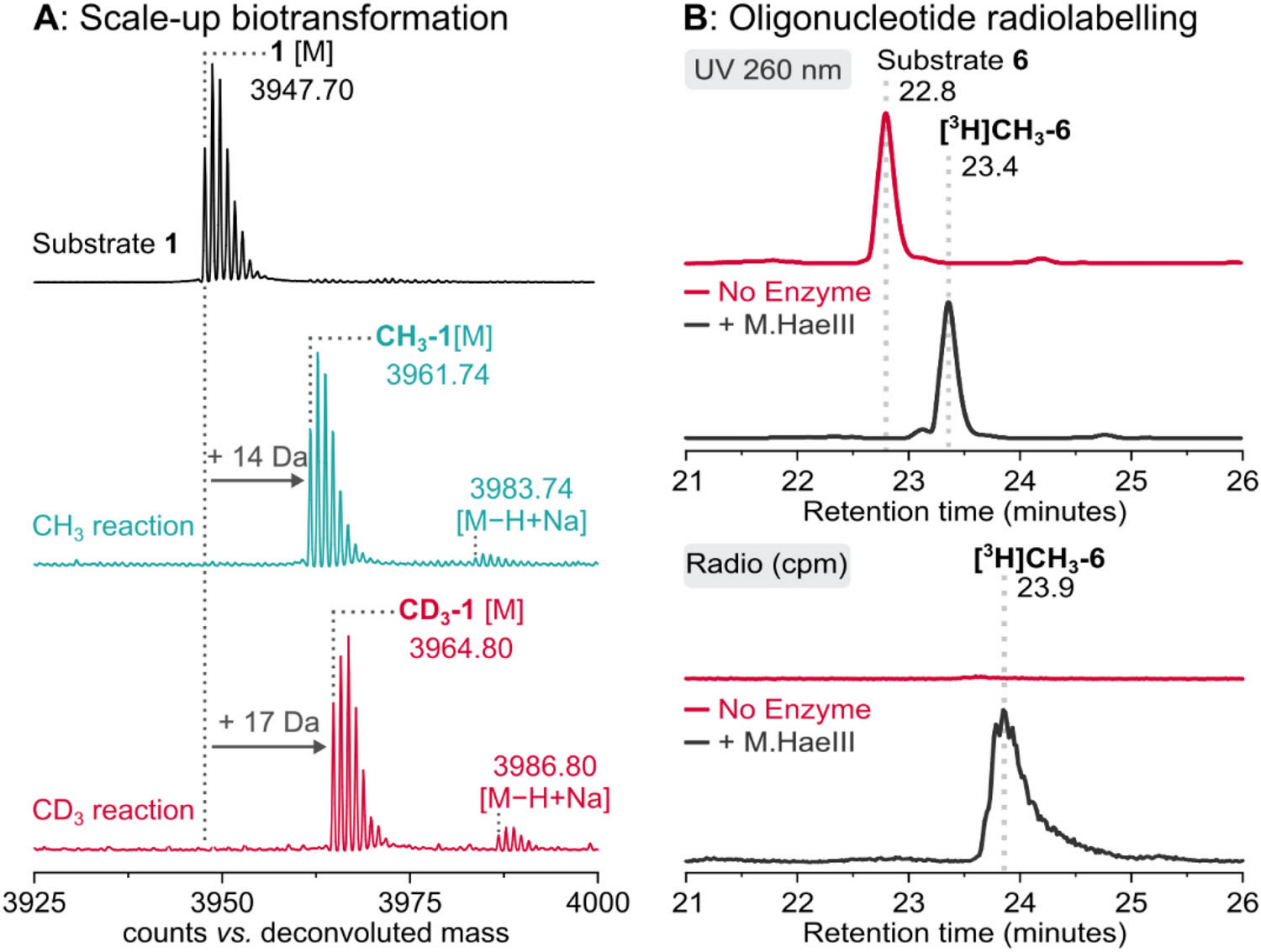
(A) Deconvoluted HRMS spectra from 2 mg scale methylation of oligonucleotide substrate **1** with M.MpeI and natural isotope abundance or trideuterated (*S*,*S*)‐SAM (both synthesised enzymatically from SAH with *Uma*). Neutral monoisotopic masses of the detected [M] and [M−H+Na] species are marked. B: HPLC traces (UV and radio detection) from enzymatic radiomethylation of oligonucleotide **6** with M.HaeIII and [^3^H]SAM (synthesised enzymatically from SAH with *Uma*). The difference in retention time (0.5 min) between the UV and radio HPLC peaks of [^3^H]CH_3_‐6 (panel **B**) is due to the physical distance between the two detectors. Full protocols, HRMS and HPLC data are available in the Supporting Information (Figures S38–S44).

A typical QWBA study in mice involves 5–10 whole‐body cryosection experiments. A radiolabelled probe is administered intravenously or subcutaneously, after which thin tissue sections are exposed to imaging plates to map the spatial distribution and accumulation of the test compound [70]. With injected doses of approximately 0.1–0.15 mg per animal (assuming a dose of 5 mg kg^−1^_body weight_), such studies require roughly 2 mg of radioactive test compound [70, 71]. To test whether methyltransferase biocatalysis could meet this requirement, we assembled reactions containing M.MpeI (2 µM), 2 mg of oligonucleotide **1** (25 µM, double‐stranded basis), and 1.5 equiv of (*S*,*S*)‐SAM or deuterated (*S*,*S*)‐[^2^H]SAM in a total volume of 10 mL (Figure 4A). ESI‐MS analysis confirmed complete conversion in both cases. Achieving quantitative methylation at this scale—approximately 1000‐fold above the screening conditions—demonstrates the robustness of the developed protocol and highlights the broader synthetic utility of DNA‐C5‐MTases as preparative biocatalysts.

In the isotope laboratory, the established protocol was applied to eight enzyme–substrate combinations using [^3^H]MeONs. As a note for practitioners: commercial [^3^H]MeONs is supplied in toluene, which must be exchanged for acetonitrile or DMSO before use, as toluene inhibits the MeONs‐accepting HMTs *Uma*, *Acl* and *Kal* (Figure S16)—most likely by competing for their flat and hydrophobic substrate‐binding pocket (see PDB: 9H06) [72]. Accordingly, [^3^H]SAM was synthesised in a 625 µL reaction containing *Uma* (10 µM), [^3^H]MeONs (1 mM, 3 TBq mmol^−1^, 81 Ci mmol^−1^) and SAH (1.2 mM) in NaPi buffer (pH 8.0) with 1% v/v DMSO or acetonitrile. The resulting solution of [^3^H]SAM was used directly or stored at −80°C for future use. Oligonucleotide ^3^H‐methylation reactions (100 µL) contained DNA‐C5‐MTase (2 µM), oligonucleotide substrate (25 µM, double‐stranded basis), and [^3^H]SAM (1.5 equiv, 75 µM). Radio‐HPLC and HPLC‐UV analysis revealed ^3^H‐radiomethylation efficiencies generally exceeded 70% (Table 1 and Figures S38–S44), with M.MpeI and M.HaeIII methylating substrates **1** and **2** to >99% (Table 1 and Figure 4B). This protocol enabled production of radiolabelled oligonucleotides with molar activities up to 3 TBq mmol^−1^ (81 Ci mmol^−1^, per strand basis), two orders of magnitude higher than custom‐synthesised oligonucleotide‐based radio probes, and four‐fold above the highest molar activity reported to date [28]. The ability to access radiolabelled oligonucleotides in up to 3 TBq mmol^−1^ (81 Ci mmol^−1^) underscores the complete retention of radioactivity in the enzyme‐catalysed synthesis of [^3^H]SAM from [^3^H]MeONs. Crucially, omitting the DNA‐C5‐MTase abolished all transfer of radioactivity to the oligonucleotide (Figure 4B). This confirms that radiomethylation is strictly enzyme‐dependent and that no radioactive oligonucleotide‐related side products are formed. A routine desalting work‐up (centrifugal filtration or isopropanol/ethanol precipitation) is sufficient to remove the proteins, small molecules and buffer salts from the reaction mixture.

**TABLE 1:** Enzymatic radiomethylation of oligonucleotides.

Substrate	Enzyme	DoL /%	MA / Ci mmol^−1^	^3^H‐Oligo:SAM /%
**1**	M.HhaI	69	55.3	69
**2**	M.HaeIII	>99	>80	75
**6**	M.HaeIII	69	55.3	71
**1**	M.MpeI	>99	>80	72
**3**	M.MpeI	97	77.7	77
**4**	M.MpeI	76	60.4	72
**3**	M.HpaII	85	67.9	74
**6**	EcDCM	28	22.3	35

DoL: Degree of labelling; MA: Molar activity, calculated per labelled oligonucleotide strand; % ^3^H‐Oligo: SAM is calculated from integration of Radio‐HPLC traces.

As the vast and largely untapped diversity of DNA‐C5‐MTases (>55,000 putative sequences, c. 500 characterised enzymes and >110 unique methylation sites identified to date) continues to be explored, we anticipate that a suitable enzyme candidate could be identified for most ASOs [37, 39]. At present, eight of nine approved ASO therapies (excluding PMOs) contain the methylation site of at least one characterised DNA‐C5‐MTase—five of which already contain m5C at the methyltransferase modification site in the parent drug (see Table S13 for an overview). The identification or engineering of novel DNA‐C5‐MTases with short methylation sites is an active field of research with applications in epigenetics, molecular biology and now also the radiolabelling of antisense oligonucleotides [73, 74].

## 3 Conclusion

The development of nucleic acid‐based therapeutics is a rapidly expanding frontier, and precise ADME profiling will grow in importance as the structural complexity of these candidates increases. To support these preclinical pipelines, this report demonstrates a modular biocatalytic methodology for late‐stage tritiation of oligonucleotides. Leveraging the utility of halide methyltransferases (HMTs), we establish a rapid and operationally simple protocol to generate stereomerically pure (*S*,*S*)‐[^3^H]SAM with high specific activity, from the inexpensive and commercially available methylation reagent [^3^H]MeONs. As a representative example, we demonstrate highly efficient ^3^H‐methylation using DNA‐C5‐MTases, generating radioactively labelled oligonucleotides with record‐breaking specific activity (3 TBq mmol^−1^; 81 Ci mmol^−1^) from only a slight excess of (S,S)‐[^3^H]SAM.

We implemented intact oligonucleotide HR‐ESI‐MS to systematically evaluate the potential of this biocatalytic platform. This enabled the key discovery that DNA‐C5‐MTases efficiently therapeutically modified oligonucleotides bearing phosphorothioate linkages, 2′‐fluoro or 2′‐MOE nucleotides at their termini and single stranded ASOs with a minimal six‐nucleotide duplex formation. This broadens the applicability of this approach to next‐generation antisense oligonucleotide gapmers within the current drug development pipeline.

Through continued development and enzyme discovery, the modular biocatalytic platform established herein lays the foundation for effective radiolabelling of therapeutic oligonucleotides, proteins, oligosaccharides, lipids and natural products. By replacing expensive, specialised synthetic processes that generate substantial radioactive waste with rapid enzymatic labelling, methyltransferase biocatalysis is poised to accelerate the preclinical evaluation of nucleic acid‐based drug candidates.

## Author Contributions

**Christopher R. B. Swanson**: investigation, methodology, validation, visualisation, writing – review and editing, formal analysis, project administration, data curation, writing – original draft. **Ulrich Kauhl**: formal analysis, methodology, writing – review and editing. **Martin R. Edelmann**: conceptualisation, investigation, funding acquisition, writing – review and editing, methodology, validation, formal analysis. **Florian P. Seebeck**: conceptualisation, investigation, funding acquisition, writing – review and editing, supervision, formal analysis, project administration.

## Funding

This project was funded by the Roche Access to Distinguished Scientists programme (ROADS).

## Conflicts of Interest

The authors declare no conflicts of interest.

## Supporting information




**Supporting File**: anie73785‐sup‐0001‐SuppMat.pdf.

## Data Availability

The data that supports the findings of this study are available in the Supporting Information of this article.
