# Supplementary material for: An Enzymatic Platform for Late‐Stage (Radio)isotope Labelling of Oligonucleotides With Methyltransferases
Source: Angew Chem Int Ed Engl. 2026 Jul 27;65(39):e8706793. doi: 10.1002/anie.8706793 (PMC13592302; doi:10.1002/anie.8706793)
Supplement: Supplementary file 1 [file ANIE-65-e8706793-s001.pdf]

Supporting information for

An Enzymatic Platform for Late-stage (Radio)isotope Labelling of Oligonucleotides with Methyltransferases.

Christopher R. B. Swanson,[a] Ulrich Kauhl,[b] Martin R. Edelmann,[b] Florian P. Seebeck[a*]

[a] Department of Chemistry, Mattenstrasse 22, University of Basel, 4058 Basel, Switzerland

[b] Therapeutic Modalities, Isotope Synthesis, Roche Innovation Center Basel, 4070, Basel, Switzerland

*Email: florian.seebeck@unibas.ch

Contents

Materials and methods 1
High-performance liquid chromatography (HPLC) 3
High-resolution electrospray ionisation mass spectrometry (HR-ESI-MS) 4
Nucleic acid precipitation method 5
Molecular Biology and Protein Production 5
Protein sequences 7
Protein production & purification 9
SDS-PAGE and HR-ESI-MS analysis 10
Biotransformation Methods 12
Scale up biotransformations 43
Method for enzymatic radiolabelling of oligonucleotides 49
References 56

Materials and methods

Unless otherwise stated, all chemicals and reagents were purchased from Sigma Aldrich and used as supplied. Antibiotics used for protein expression were purchased from PanReac AppliChem. Isopropyl β-D-thiogalactoside (IPTG) was purchased from Acros Organics. [^{3}H]Methyl nosylate was purchased from RC Tritec AG. Roti®garose-His/Ni beads was purchased from ROTH (Art. # 1308.2). Buffer ingredients were purchased from Acros Organics. Molecular weight marker for SDS-PAGE was purchased from BioRad (Precision plus blue or dual colour protein standard). Molecular biology reagents (Q5® High-Fidelity 2X Master Mix and NEBuilder® HiFi DNA Assembly Master Mix) were purchased from NEB (*via* BioConcept). Plasmid DNA was purified using PureYield plasmid miniprep system (Promega) and sequenced by Microsynth AG. All synthetic oligonucleotides were purchased from Microsynth AG with desalting work-up (except for modified oligonucleotides which were ordered with HPLC purification).

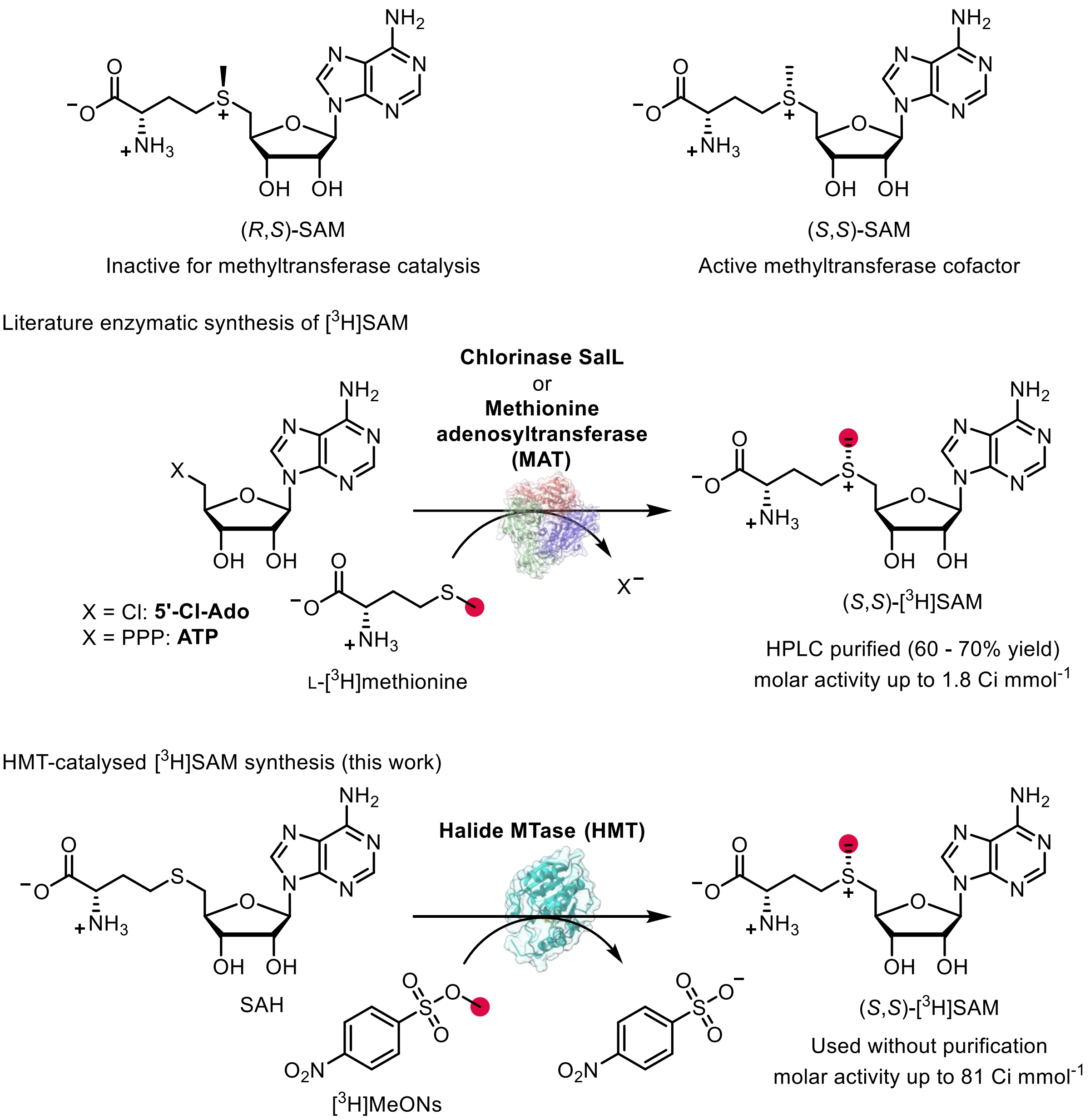

Figure S1: Structures of (*R*,*S*)- and (*S*,*S*)-SAM, comparison of literature methods[1] for [^{3}H]SAM synthesis and the HMT-based method developed herein.

Table S1: Comparison of availability and costs of relevant radiochemical reagents.

^{3}H-Compound	Listed pack sizes (mCi)	List price (CHF mCi^{-1})
[^{3}H]SAM	0.1 – 1	2,500
L-[^{3}H]methionine	0.25 – 1	1672
[^{3}H]MeONs	20 – 1000	21.2

As accessed from suppliers RC Tritec AG or Revvity (2026-04-09). List price calculated using the largest available pack size.

High-performance liquid chromatography (HPLC)

Cation exchange HPLC (IEX-HPLC) was recorded with a Shimadzu prominence HPLC paired with cation-exchange LC column 150 × 4.6 mm (Luna 5 µm SCX 100 Å). The mobile phase was a mixture of buffer A (20 mM H_3PO_4) and buffer B (20 mM H_3PO_4, 1 M NaCl, pH 2, filtered with 0.22 µm MCE Membrane MF-Millipore™). The HPLC traces were exported as ASCII files and processed in Origin2024b.

Table S2: Ion Exchange HPLC-UV method used for analysis of SAM formation in this work. Buffer A = 20 mM H_3PO_4, buffer B = 20 mM H_3PO_4, 1 M NaCl, pH 2, filtered with 0.22 µm MCE Membrane MF-Millipore™. This method was applied with a Luna 5 µm SCX 100 Å, 150 x 4.6 mm column as stationary phase and a flow rate of 1 mL min^{-1}.

Time / min	% solvent A	% solvent B
0.0	90	10
0.5	90	10
9.0	60	40
11	1	99
15.0	1	99
15.5	90	10
17.0	90	10

Table S3: Ion Exchange HPLC-UV method 2 used for analysis of SAM formation in this work. Buffer A = 20 mM H_3PO_4, buffer B = 20 mM H_3PO_4, 1 M NaCl, pH 2, filtered with 0.22 µm MCE Membrane MF-Millipore™. This method was applied with a Luna 5 µm SCX 100 Å, 150 x 4.6 mm column as stationary phase and a flow rate of 1 mL min^{-1}.

Time / min	% solvent A	% solvent B
0.0	70	30
0.5	70	30
3.5	60	40
4.5	1	99
8.5	1	99
9.0	70	30
10	70	30

HPLC-UV-Radio analysis of oligonucleotides was performed on an Agilent HPLC equipped with an ACQUITY Premier Peptide BEH 300Å C18 column (1.7 µm, 2.1 x 50 mm). The mobile phase was a mixture of solvent A (15 mM amylamine (pentylamine) and 200 mM HFIP in 980 mL water) and solvent B (9:1 MeOH/Acetonitrile) according to the gradient in Table S4.

Table S4: HPLC-UV-Radio method used for analysis of radioactive biotransformations in this work. Solvent A = 15 mM amylamine (pentylamine) and 200 mM HFIP in 980 mL water, solvent B = 9:1 MeOH/Acetonitrile. This method was applied on an Agilent 1200 HPLC system with an ACQUITY Premier Peptide BEH 300A C18 column (1.7 µm, 2.1 x 50 mm) as stationary phase. The column oven was set to 80 °C, the flow rate 0.4 mL min^{-1} and the post run time was 3 minutes.

Time / min	% solvent A	% solvent B
0.0	95	5
3.0	95	5
25.5	80	20
31	100	0
33	100	0

High-resolution electrospray ionisation mass spectrometry (HR-ESI-MS)

HR-ESI-MS spectra were obtained on a Bruker maXis 4G UHR-TOF mass spectrometer coupled with a Shimadzu Nexera X2 series LC.

For proteins, samples were prepared at 0.2 mg mL^{-1} in 0.1 % (v/v) formic acid in MilliQ water and 1 µL sample was injected. A ReproSil Gold 30 C4 column (5 µm, 50 x 2 mm, Dr. Maisch) was used as stationary phase and the mobile phase was a mixture of buffer A (0.1% formic acid in milliQ water) and buffer B (0.1% formic acid in MeCN) according to the gradient in Table S5 at a flow rate of 0.5 mL min^{-1}. The mass spectrometer was operated in positive mode using sodium formate as an internal calibrant.

Table S5: LC-HR-ESI-MS method used for protein analysis in this work. Buffer A = dH_2O + 0.1% formic acid, buffer B = Acetonitrile + 0.1% formic acid. This method was used with a ReproSil Gold 30 C4 (5 µm, 50 x 2 mm, Dr. Maisch) column as stationary phase.

Time / min	% solvent A	% solvent B
0.00	90	10
1.0	90	10
4.5	45	55
5.5	45	55
6.5	5	95
7.5	5	95
8.5	90	10
10.0	90	10

For oligonucleotides, no column was used and the sample was routed directly into the MS source. The mobile phase consisted of 1:1 milliQ water/MeCN (no additives) with a flow rate of 0.4 mL min^{-1}. The cycle time was 1 minute per sample. The mass spectrometer was operated in negative mode using sodium formate as an internal calibrant and the settings outlined in Table S6.

Table S6: MS settings used on the Bruker maXis instrument for oligonucleotide analysis

MS setting	Value
Mass Range	300 – 3000 *m/z*
Source	ESI
End plate offset	500 V
Nebuliser	4 Bar
Dry temp	220 °C
Capillary	4500 V
Dry gas	8.0 L min^{-1}

Nucleic acid precipitation method

Prior to HR-ESI-MS analysis, oligonucleotides from enzymatic reactions were desalted *via* precipitation using the following protocol in either 8-well PCR tube strips or 96-well PCR plates.

1. The salt concentration of the oligonucleotide solution (5 µL) was adjusted with an ammonium acetate and glycogen solution (5 µL, [NH_4OAc]: 5 M stock, 2.5 M final; [glycogen] 2 mg mL^{-1} stock, 1 mg mL^{-1} final) and mixed gently.
2. Room-temperature isopropanol (10 µL) was added and the solution mixed gently. The sample was immediately centrifuged at 21,300 x g (max speed) for 15 minutes at 4 °C (8-well strips) or 5,250 x g (max speed) for 60 minutes at 4°C (96-well PCR plates).
3. The supernatant was decanted and the pellet washed with cold 70% ethanol (100 µL).
4. The sample was centrifuged again at 21,300 x g (max speed) for 15 minutes at 4 °C (8 well strips) or 5,250 x g (max speed) for 30 minutes at 4°C (96-well PCR plates).
5. The supernatant was decanted and the pellet was air-dried for 5 minutes.
6. The DNA was dissolved to a concentration of c. 20 µM in **MQ water** and transferred into vials or 96-well plates for HR-ESI-MS analysis. Optionally, 1 volume of a 20 mM imidazole and 1 v% Et_3N in acetonitrile solution was added to improve MS sensitivity.
7. 1 – 5 µL of sample was injected into the HR-ESI-MS.

Molecular Biology and Protein Production

E. coli codon optimised gene fragments for each of the DNA-C-MTases were purchased from Twist Bioscience. Genes were amplified by PCR with NEB Q5 polymerase (2x master mix) and oligonucleotide primers (Microsynth AG). Plasmid DNAs were assembled *via* HiFi DNA assembly (NEB), used to transform chemically competent *E. coli* TOP10 cells, and purified by mini-prep (Promega PureYield kit) and identity confirmed by Sanger or ONT sequencing (Microsynth AG).

Table S7: Oligonucleotide primers used in this work

Name	Sequence 5' – 3'
HhaI_HiFi_F	GAAGGAGATATACCATGGGCATGATTGAAATAAAAGATAAACAGC
HhaI_HiFi_R	TGGTGGTGGTGGTGCTCGAGATACGGTTTAAAGTTCAGAC
HaeIII_HiFi_F	GAAGGAGATATACCATGGGCATGAACTTAATCTCTTTATTTAGTGGCG
HaeIII_HiFi_R	TGGTGGTGGTGGTGCTCGAGAAGGGCTGACTTTATAGTC
MpeI_HiFi_F	GAAGGAGATATACCATGGGCATGAACTCGAACAAAGATAAAATTAAAG
MpeI_HiFi_R	TGGTGGTGGTGGTGCTCGAGTTCTTCGTTATTCACAAATTCAAGCG
HpaII_HiFi_F	GAAGGAGATATACCATGGGCATGAAAGACGTACTGGATGATAATCTCTTAGAAG
HpaII_HiFi_R	TGGTGGTGGTGGTGCTCGAGATCATAGAGGTTCCCAAGTTTTTCCAAGATC
HpyIII_HiFi_F	GAAGGAGATATACCATGGGCATGGGTATTTTAACTTTTATGGATTTTTGCT
HpyIII_HiFi_R	TGGTGGTGGTGGTGCTCGAGTTCATTATTAAAAGCTTTCAGCATCTG
EcDCM_HiFi_F	GAAGGAGATATACCATGGGCATGCAGGAAAACATTAGCGTCACTGATTCTTACTC
EcDCM_HiFi_R	TGGTGGTGGTGGTGCTCGAGTCGTGAACGTCGCCCGTGCTG
pET28_HiFi_F	CTCGAGCACCACCACCAC
pET28_HiFi_R	GCCCATGGTATATCTCCTTCTTAAAG

For protein production, chemically competent *E. coli* cells (BL21, Δmtn or NEB T7express) were transformed with plasmid DNA containing genes for the desired protein and grown on LB agar plates supplemented with 1 w% D-glucose and 50 µg mL^{-1} kanamycin as shown in Table S8. Unless specified, all media contained 1 % w/v D-glucose to supress leaky expression.

Table S8**:** Proteins used in this work. T7 express cells were purchased from New England Biolabs (cat no. C25666H), Δmtn *E. coli* cells were derived from those generated in a previous study.[2]

Name	Uniprot ID	Species	Recognition site	Cell strain
M.HhaI	P05102	*Haemophilus haemolyticus*	GCGC	T7express
M.HaeIII	P20589	*Haemophilus aegyptius*	GGCC	T7express
M.MpeI	Q8EVR5	*Malacoplasma penetrans*	CG	T7express
M.HpaII	P15446	*Haemophilus parainfluenzae*	CCGG	T7express
M.Hpy99III	Q9ZK91	*Helicobacter pylori*	GCGC	T7express
*Ec*DCM	P0AED9	*E. coli*	CCWGG (W = A or T)	T7express
*Ec*MTAN	P0AF12	*E. coli*	-	Δmtn(DE3)
*Uma*HMT	A0A0D1DT00	*Ustilago maydis*	-	Δmtn(DE3)
*Acl*HMT	A1CIS5	*Aspergillus clavatus*	-	Δmtn(DE3)
*Kal*HMT	A9DNE6	*Kordia algicida*	-	Δmtn(DE3)

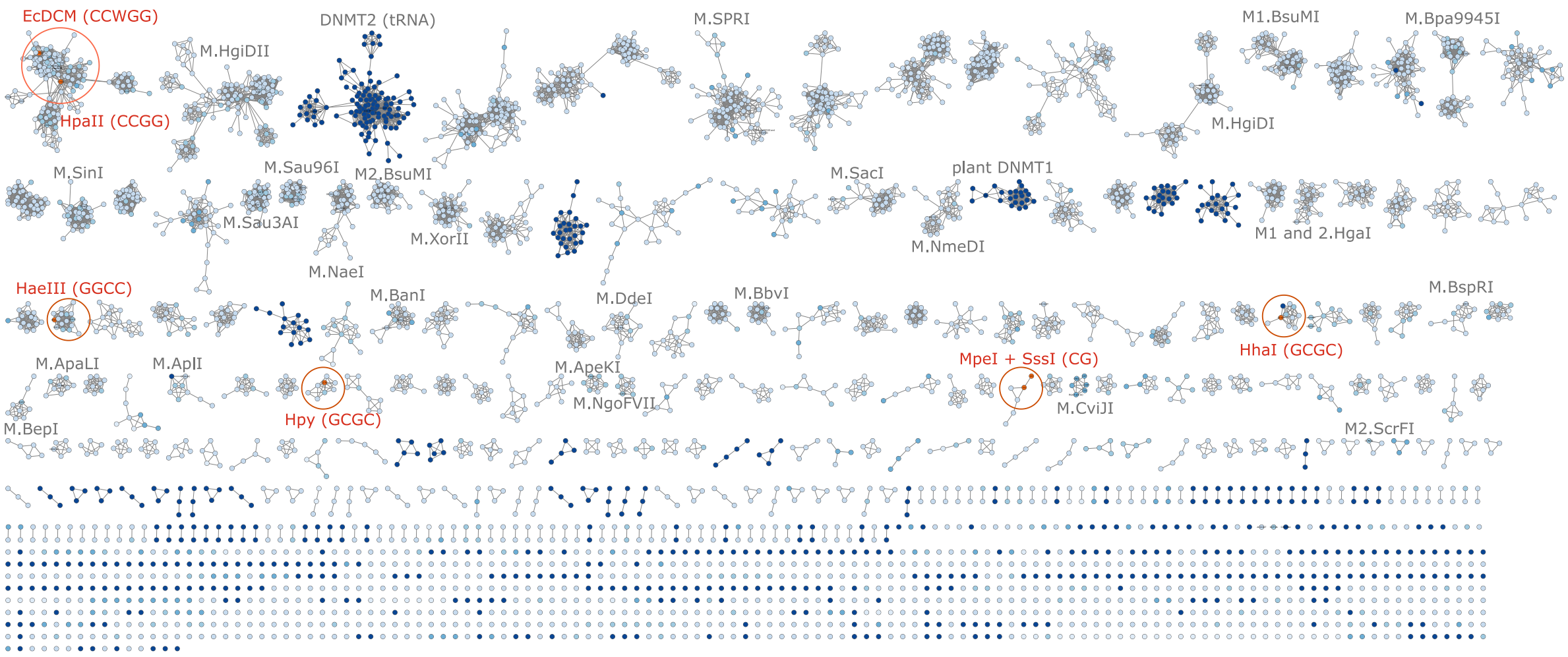

Figure S2: Sequence similarity network of DNA-C5-MTases generated using EFI-EST with PFAM00145. Each node corresponds to a UniRef50 identifier. The alignment score was set at 75 and the sequence length was trimmed to 280 – 500 amino acids to capture single domain DNA-C5-MTases. Proteins from the panel explored in this work are marked in orange.

Protein sequences

Sequences marked in bold originate from the vector backbone.

>M.HhaI (Δ324G truncated sequence described by Daujotye *et al.*)[3]
MGMIEIKDKQLTGLRFIDLFAGLGGFRLALESCGAECVYSNEWDKYAQEVYEMNFGEKPEGDITQVNEKTIPDHDI
LCAGFPCQAFSISGKQKGFEDSRGTLFFDIARIVREKKPKVVFMENVKNFASHDNGNTLEVVKNTMNELDYSFHAK
VLNALDYGIPQKRERIYMICFRNDLNIQNFQFPKPFELNTFVKDLLLPDSEVEHLVIDRKDLVMTNQEIEQTTPKTVR
LGIVGKGGQGERIYSTRGIAITLSAYGGGIFAKTGGYLVNGKTRKLHPRECARVMGYPDSYKVHPSTSQAYKQFGNS
VVINVLQYIAYNIGSSLNG**LEHHHHHH**

>M.HaeIII
MGMNLISLFSGAGGLDLGFQKAGFRIICANEYDKSIWKTYESNHSAKLIKGDISKISSDEFPKCDGIIGGPPCQSWSE
GGSLRGIDDPRGKLFYEYIRILKQKKPIFFLAENVKGMMAQRHNKAVQEFIQEFDNAGYDVHIILLNANDYGVAQD
RKRVFYIGFRKELNINYLPPIPHLIKPTFKDVIWDLKDNPIPALDKNKTNGNKCIYPNHEYFIGSYSTIFMSRNRVRQW
NEPAFTVQASGRQCQLHPQAPVMLKVSKNLNKFVEGKEHLYRRLTVRECARVQGFPDDFIFHYESLNDGYKMIGN
AVPVNLAYEIAKTIKSAL**LEHHHHHH**

>M.MpeI
MGMNSNKDKIKVIKVFEAFAGIGSQFKALKNIARSKNWEIQHSGMVEWFVDAIVSYVAIHSKNFNPKIERLDRDILS
ISNDSKMPISEYGIKKINNTIKASYLNYAKKHFNNLFDIKKVNKDNFPKNIDIFTYSFPCQDLSVQGLQKGIDKELNTRS
GLLWEIERILEEIKNSFSKEEMPKYLLMENVKNLLSHKNKKNYNTWLKQLEKFGYKSKTYLLNSKNFDNCQNRERVF
CLSIRDDYLEKTGFKFKELEKVKNPPKKIKDILVDSSNYKYLNLNKYETTTFRETKSNIISRPLKNYTTFNSENYVYNINGI
GPTLTASGANSRIKIETQQGVRYLTPLECFKYMQFDVNDFKKVQSTNLISENKMIYIAGNSIPVKILEAIFNTLEFVNN
EE**LEHHHHHH**

>M.HpaII
MGMKDVLDDNLLEEPAAQYSLFEPESNPNLREKFTFIDLFAGIGGFRIAMQNLGGKCIFSSEWDEQAQKTYEANF
GDLPYGDITLEETKAFIPEKFDILCAGFPCQAFSIAGKRGGFEDTRGTLFFDVAEIIRRHQPKAFFLENVKGLKNHDKG
RTLKTILNVLREDLGYFVPEPAIVNAKNFGVPQNRERIYIVGFHKSTGVNSFSYPEPLDKIVTFADIREEKTVPTKYYLS
TQYIDTLRKHKERHESKGNGFGYEIIPDDGIANAIVVGGMGRERNLVIDHRITDFTPTTNIKGEVNREGIRKMTPRE
WARLQGFPDSYVIPVSDASAYKQFGNSVAVPAIQATGKKILEKLGNLYD**LEHHHHHH**

>M.Hpy99III
MGMGILTFMDFCSGIGGGRLGLERCHLKCVGHAEINHEAIRTYELFFKDTHNFGDLMRINPNDLPNFDVLVSGFPC
QAFSINGKRKGLEDERGTIIYGLIRILKVKQPKCFLLENVKGLIHHKQQETFKTIIKALQEAGYTTHYQILNSADFQLAQ
KRERLYIVGFRKDLKRPFNFPLGLANDYCFKDFLDADNECYLDVSNATFQRYLRNPYNHNRVFLEDILTLENAVLDTR
QSDLRLYFNVFPTLRTSRHGLFYTQKGKIKRLNAVESLLLQGFPRDLIAKIKNNPNFKESHLLSQAGNAMSVNVIAAI
AKQMLKAFNNE**LEHHHHHH**

>EcDCM (M.EcoDCM)
MGMQENISVTDSYSTGNAAQAMLEKLLQIYDVKTLVAQLNGVGENHWSAAILKRALANDSAWHRLSEKEFAHL
QTLLPKPPAHHPHYAFRFIDLFAGIGGIRRGFESIGGQCVFTSEWNKHAVRTYKANHYCDPATHHFNEDIRDITLSH
KEGVSDEAAAEHIRQHIPEHDVLLAGFPCQPFSLAGVSKKNSLGRAHGFACDTQGTLFFDVVRIIDARRPAMFVLE
NVKNLKSHDQGKTFRIIMQTLDELGYDVADAEDNGPDDPKIIDGKHFLPQHRERIVLVGFRRDLNLKADFTLRDISE
CFPVLWKYLYRYAKKHQARGNGFGYGMVYPNNPQSVTRTLSARYYKDGAEILIDRGWDMATGEKDFDDPLNQQ
HRPRRLTPRECARLMGFEAPGEAKFRIPVSDTQAYRQFGNSVVVPVFAAVAKLLEPKIKQAVALRQQEAQHGRRS
R**LEHHHHHH**

>EcMTAN
MGSSHHHHHHSSGLVPRGSHMKIGIIGAMEEEVTLLRDKIENRQTISLGGCEIYTGQLNGTEVALLKSGIGKVAAAL
GATLLLEHCKPDVIINTGSAGGLAPTLKVGDIVVSDEARYHDADVTAFGYEYGQLPGCPAGFKADDKLIAAAEACIA

ELNLNAVRGLIVSGDAFINGSVGLAKIRHNFPQAIAVEMEATAIAHVCHNFNVPFVVVRAISDVADQQSHLSFDEFL
AVAAKQSSLMVESLVQKLAHG

>UmaHMT
MTSSLSKDDQIQNLRRLFADSGVPNDPKAWDQAWIDSTTPWDANRPQPALVELLEGAHDADAKVPDVDGNLIP
VSQAIPKGDGTAVVPGCGRGYDARVFAERGLTSYGVDISSNAVAAANKWLGDQDLPTELDDKVNFAEADFFTLGT
SKSLVLELSKPGQATLAYDYTFLCAIPPSLRTTWAETYTRLLAKHGVLIALVFPIHGDRPGGPPFSISPQLVRELLGSQK
NADGSAAWTELVELKPKGPETRPDVERMMVWRRS**LEHHHHHH**

>AclHMT
MSTPSLIPSGVHEVLAKYKDGNYVDGWAELWDKSKGDRLPWDRGFPNPALEDTLIQKRAIIGGPLGQDAQGKTY
RKKALVPGCGRGVDVLLLASFGYDAYGLEYSATAVDVCQEEQAKNGDQYPVRDAEIGQGKITFVQGDFFEDTWLE
KLNLTRNCFDVIYDYTFFCALNPSMRPQWALRHTQLLADSPRGHLICLEFPRHKDPSVQGPPWGSASEAYRAHLSH
PGEEIPYDASRQCQFDSSKAPSAQGLERVAYWQPERTHEVGKNEKGEVQDRVSIWQRPPQSSL**LEHHHHHH**

>KalHMT
MNSDATKEYWSQRYKDNSTGWDIGSPSTPLKTYIDQLKDRNLKILIPGAGNAYEAEYLLQQGFTNIYILDISEIPLQEF
KQRNPEFPSDRLLCDDFFTHKNTYDLIIEQTFFCSFPPLPETRAQYAKHMADLLNPNGKLVGLWFDFPLTDDLEKRP
FGGSKEEYLEYFKPYFDVKTFEKAYNSIAPRAGNELFGIFIKS**LEHHHHHH**

Protein production & purification

DNA-C5-MTases: A single colony of T7express *E. coli* cells harbouring the desired plasmid was added to 5 mL LB medium supplemented with 1 w% D-glucose and 50 µg mL^{-1} kanamycin and incubated overnight at 200 rpm and 37 °C. The preculture was diluted 100x into TB media (50 – 500 mL), supplemented with 1 w% D-glucose and 50 µg mL^{-1} kanamycin, in a baffled flask (250 mL – 3 L flask volume) and incubated at 37 °C, 200 rpm until optical cell density (OD600) of 0.6 – 0.8 was reached. At this point, the flasks were cooled in an ice bath (c. 15 minutes) and protein production was induced by addition of isopropyl-β-D-1-thiogalactopyranoside (IPTG, 0.5 mM final concentration). The cultures were then incubated at 18 °C, 200 rpm for 18 h. The cells were harvested by centrifugation at 17,568 x g for 15 min at 4 °C. The cell pellets were stored at -20 °C in 50 mL falcon tubes until further use.

For lysis and purification, cell pellets were thawed and resuspended in 5 mL $g^{-1}_{\text{cell wet weight}}$ of 4% Buffer B (50 mM NaPi buffer, 750 mM NaCl, 10 mM imidazole, 10% v/v glycerol, pH 8.0) and lysed by ultra-sonication (5 s ON, 5 s OFF, 10 minutes total time) while samples were submerged in an ice bath or cooling block. The lysed cells were clarified by centrifugation at 47,850 x g for 45 min at 4 °C. The clarified lysate was loaded onto a prewashed column of ROTIGarose-His/Ni NTA beads (Carl ROTH, 0.5 – 1 mL resin bed) and the flow through passed through the column a second time. The resin bed was washed sequentially with 10 column volumes 4% buffer B (50 mM NaPi buffer, 750 mM NaCl, 10 mM imidazole, 10% v/v glycerol, pH 8.0) and 10 column volumes 10 % buffer B (50 mM NaPi buffer, 750 mM NaCl, 25 mM imidazole, 10 % v/v glycerol, pH 8.0). The His-tagged protein was then eluted in fractions with 100% buffer B (50 mM NaPi buffer, 750 mM NaCl, 250 mM imidazole, 10 % v/v glycerol, pH 8.0). Fractions containing protein as determined by Nanodrop (using calculated molecular weights and extinction coefficients from ProtParam) were pooled for dialysis at 4 °C against NaPi buffer (50 mM, pH 8.0) containing 150 mM NaCl and 1 mM each EDTA and DTT. The pure protein was analysed by SDS-PAGE and HRMS, divided into aliquots, flash frozen and stored at −70 °C until further use. Expression yields of proteins ranged from 10 – 200 mg L^{-1} of culture broth.

HMTs and MTAN: A single colony of Δmtn(DE3) *E. coli* cells harbouring the desired plasmid was added to 5 mL LB medium supplemented and 50 µg mL^{-1} kanamycin and incubated overnight at 200 rpm and 37 °C. The preculture was diluted c. 100x into TB media (50 – 500 mL), supplemented with 50 µg mL^{-1} kanamycin, in a baffled flask (250 mL – 3 L flask volume) and incubated at 37 °C, 200 rpm until optical cell density (OD600) of 0.6 – 0.8 was reached. At this point, protein production was induced by addition of isopropyl-β-D-1-thiogalactopyranoside (IPTG, 0.5 mM final concentration). The cultures were then incubated at 25 °C, 200 rpm for 18 h.

The lysis and purification were performed as described above except the buffers used were:

Lysis/ 4% buffer B_{HMT}: 50 mM NaPi, 300 mM NaCl, 10 mM imidazole, pH 8.0

10% buffer B_{HMT}: 50 mM NaPi, 300 mM NaCl, 25 mM imidazole, pH 8.0

100% buffer B_{HMT}: 50 mM NaPi, 300 mM NaCl, 250 mM imidazole, pH 8.0

Dialysis $buffer_{HMT}$: 50 mM NaPi, pH 8.0

SDS-PAGE and HR-ESI-MS analysis

SDS-PAGE was performed on gels containing a 12% acrylamide resolving phase and 4% acrylamide stacking phase. A standard 1x TGS buffer was used as the running buffer (25 mM Tris, 192 mM glycine, 0.1 % w/v SDS). Samples were boiled in 1x SDS-PAGE loading dye. The molecular weight marker was precision plus blue or dual colour protein standard (BioRad).

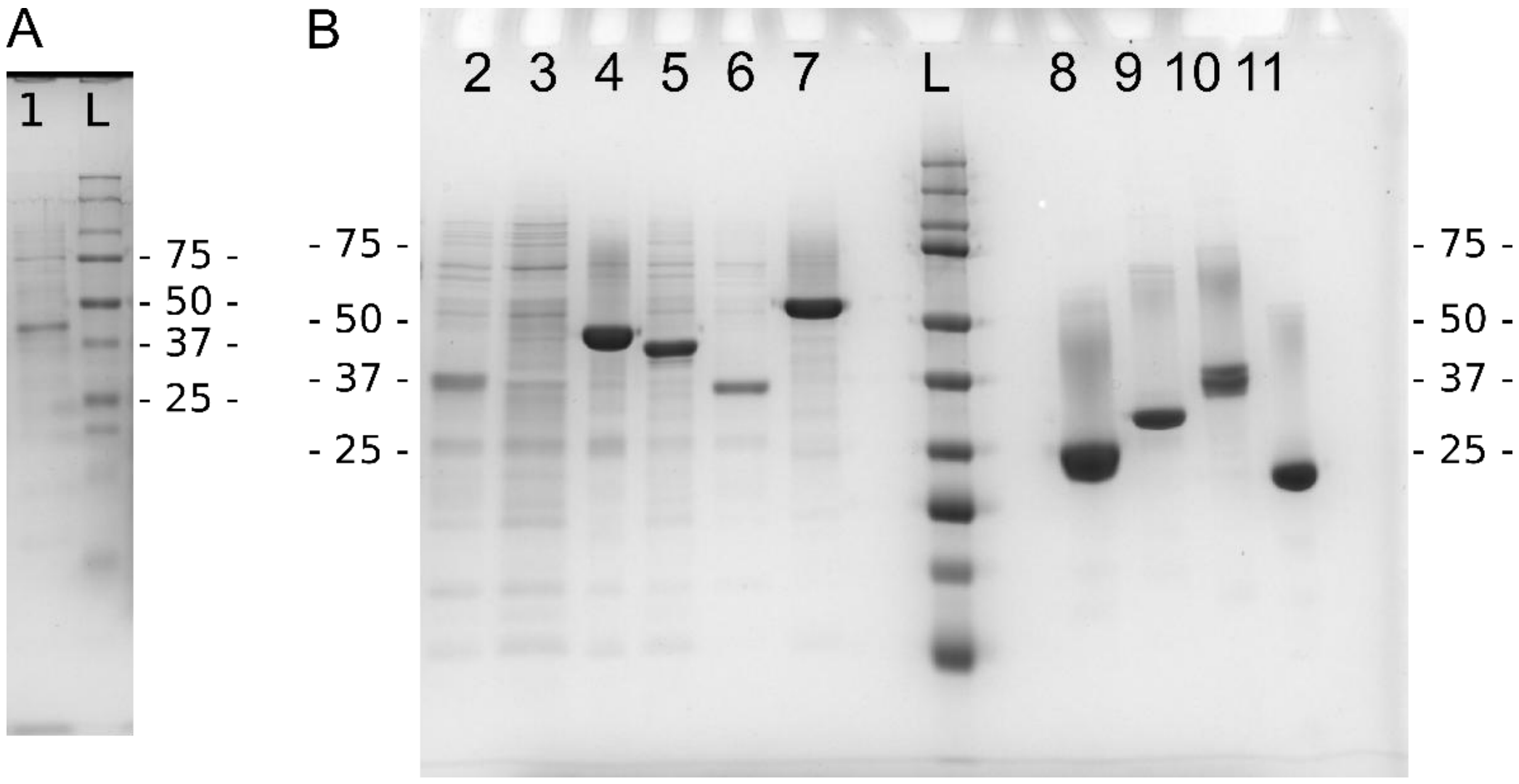

Figure S3: SDS-PAGE analysis of proteins used in this work. Protein samples (left to right) A: 1 – M.HhaI (37 kDa), B: 2 – M.HaeIII (38 kDa); 3 – empty pET28 expression control; 4 – M.MpeI (47 kDa); 5 – M.HpaII (41 kDa); 6 – M.Hpy99III (38 kDa); 7 – EcDCM (54 kDa); 8 – EcMTAN (26 kDa); 9 – Uma (29 kDa); 10 – Acl (33 kDa); 11 – Kal (24 kDa). Lanes marked 'L' were loaded with ProteinPlus standard (BioRad).

Table S9: Calculated exctinction coefficents and molecular weights of produced proteins and observed molecular weight by HRMS.

Name	Uniprot ID	Extinction coefficient (ε, $M^{-1} cm^{-1}$)	Molecular weight (calc, Da)	Molecular weight (obs, Da)
M.HhaI	P05102	27850	37640	37639
M.HaeIII	P20589	44350	38164	38164
M.MpeI	Q8EVR5	50310	47228	47227
M.HpaII	P15446	30370	41522	41521
M.Hpy99III	Q9ZK91	16390	38042	38041
*Ec*DCM	P0AED9	49850	54587	54585
*Ec*MTAN	P0AF12	5960	26385	26385
*Uma*HMT	A0A0D1DT00	45950	29390	29388
*Acl*HMT	A1CIS5	61880	33265	33263
*Kal*HMT	A9DNE6	34380	24228	24228

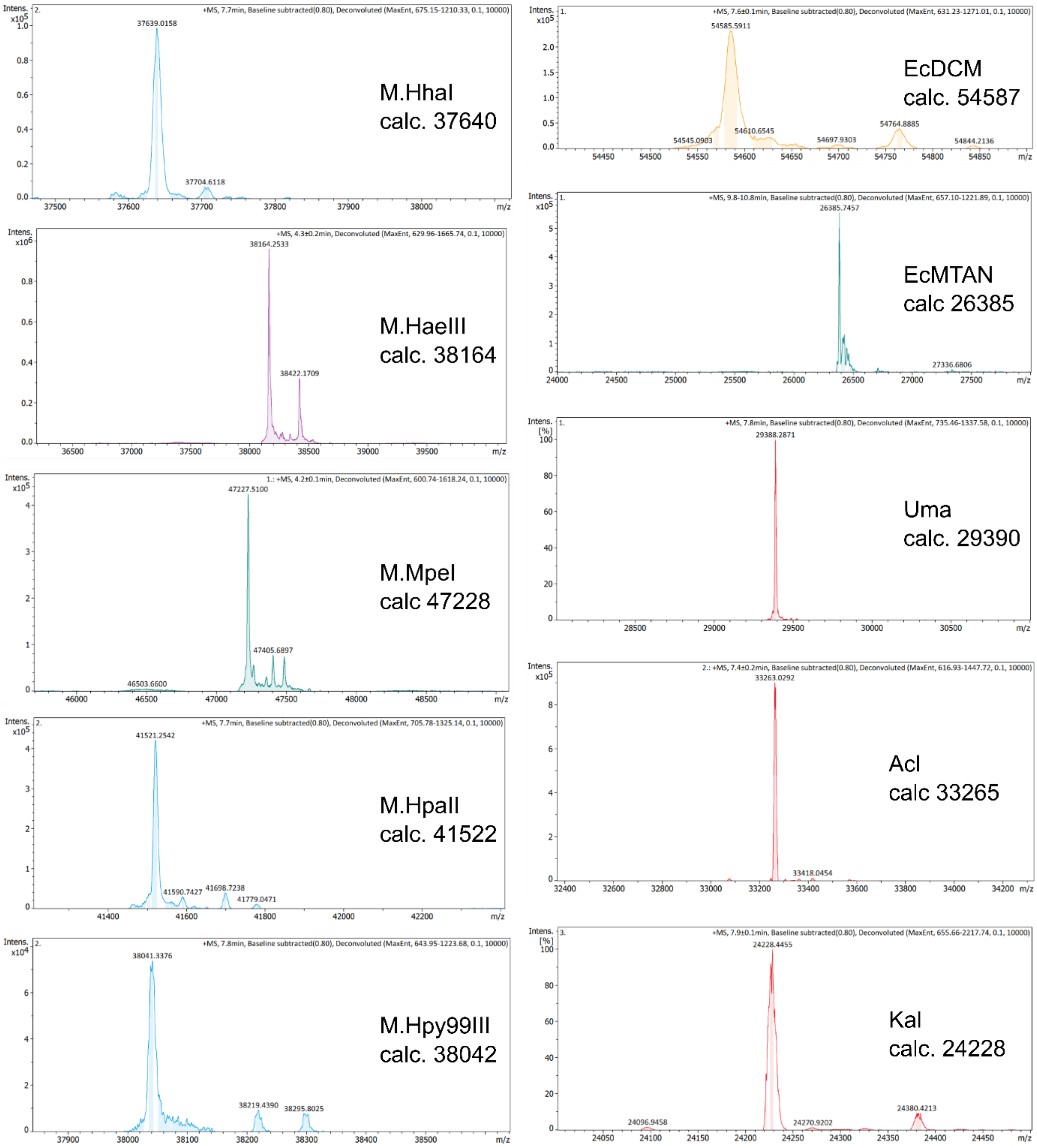

Figure S4: HRMS analysis of proteins used in this work.

Biotransformation Methods

Table S10: Sequences of oligonucleotides used as substrates in this work. Regions shown in bold correspond to a DNA-C-MTase recognition site and the underlined C base expected to be methylated.

Oligonucleotide	Sequence (5' – 3')	Monoisotopic mass / Da
1	TGATA**GCGC**TATC	3947.70
1a	TGATA**GMGC**TATC	3961.71
1b	TGATA**GCGM**TATC	3961.71
2	TGATA**GGCC**TATC	3947.70
3	TGATA**CCGG**TATC	3947.70
4	TGATCCT**CG**AGGATC	4565.80
5[a]	TGAT**CCTGGCCAGG**ATC	5183.90
6[a]	TGAT**CCAGGCCTGG**ATC	5183.90
7 strand a	TGAT**CCTGG**TATC	3938.69
7 strand b	GGATA**CCAGG**ATC	3981.72
8 strand a	TGAT**CCGG**GTATC	3963.69
8 strand b	TGATAC**CCGG**ATC	3932.70
9	TGATAGC*GCTATC	3963.68
10	TGATAG*CGCTATC	3963.68
11	TGATAG*C*GCTATC	3979.65
12	TGATA**GrCGC**TATC	3963.69
13	TGATA**GfCGC**TATC	3965.69
14	TGATA**GmCGC**TATC	3977.71
15	T*G*ATAGCGCTATC	3979.65
16	fUfGATA**GCGC**TAfUfC	3991.63
17	oToGATA**GCGC**TAoToC	4257.84
18	TATC**CG**GAGGGCT**CG**CCATGCTGCT	7646.28
18a	TATC**MG**GAGGGCT**CG**CCATGCTGCT	7660.29
18b	TATC**CG**GAGGGCT**MG**CCATGCTGCT	7660.29

Oligonucleotides 1 – 6 and 9 – 17 contain palindromic nucleobase sequences and form self-complimentary duplexes. dsDNAs for oligonucleotides 7 and 8 were formed by annealing the two complementary strands (a and b). Unless indicated, all oligonucleotides are formed of standard DNA nucleotides. Where marked, the following modifications apply at the specified position: M = 5-methylcytosine base; * = racemic phosphorothioate linkage; r = RNA nucleotide; f = 2'-fluoro nucleotide; m = 2'-methoxy nucleotide; o = 2’-methoxyethyl (MOE) nucleotide. [a] Oligonucleotides 5 and 6 contain two copies of the EcDCM recognition site (CCWGG), and one copy of the M.HaeIII recognition site (substrate 5: TGATCCT**GGCC**AGGATC; substrate 6: TGATCCA**GGCC**TGGATC).

High-throughput screening of DNA-MTases

Screening reactions were performed in 96-well PCR plates with 10 µL reaction volumes. Each reaction well contained; DNA-C5-MTase (2 µM), oligonucleotide substrate (25 µM, dsDNA basis), SAM (400 µM, commercial preparation containing a mixture of sulphur stereoisomers), DTT (1 mM), EDTA (1 mM) and NaPi buffer (50 mM, pH 8.0). The reaction plates were incubated at 37 °C for 1 hour in a PCR thermocycler (no mixing). Samples were prepared for HRMS by DNA precipitation, transferred into 96-well sample plates and analysed by HR-ESI-MS as outlined in “Materials and methods”. The HRMS spectra were processed in Bruker Data compass and Origin2024.

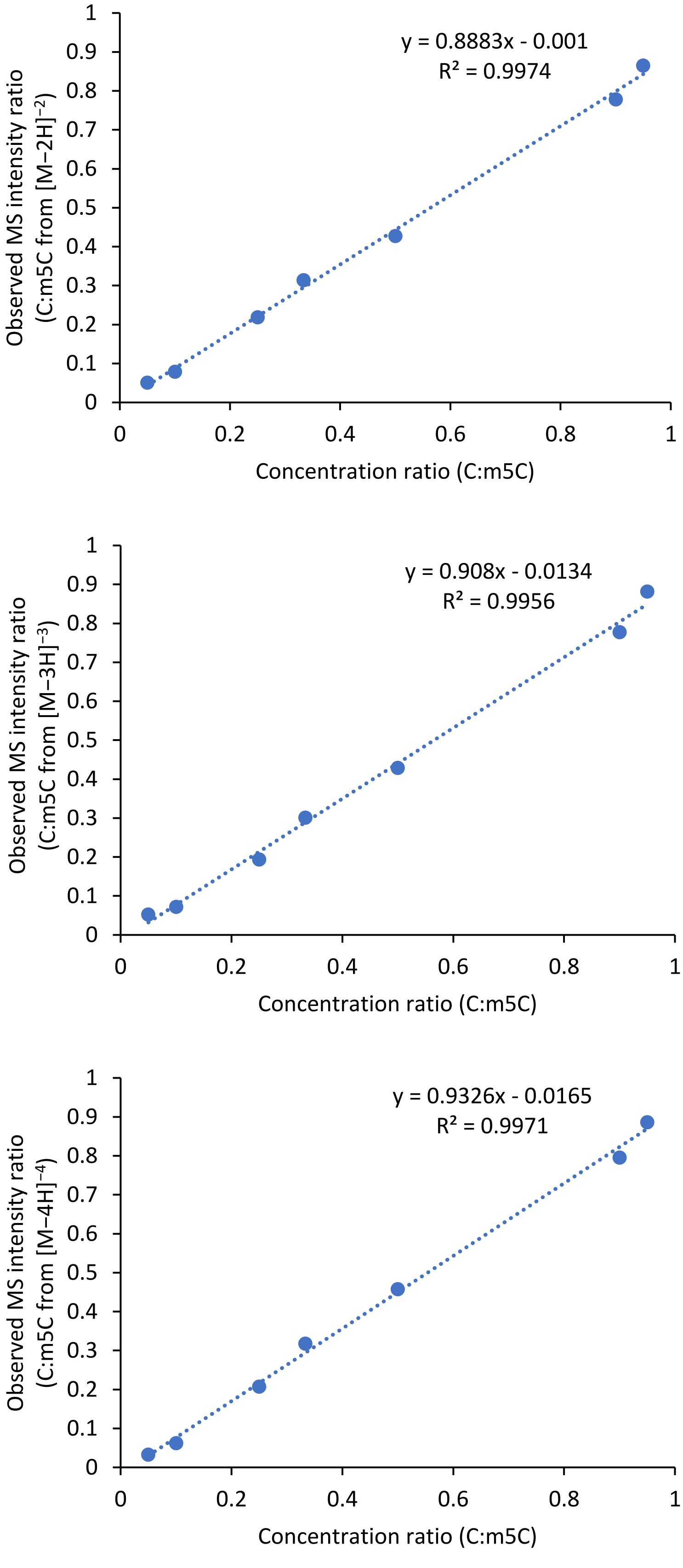

Figure S5: Analysis of defined mixtures of unmethylated and methylated oligonucleotide **1** demonstrating the linearity of quantification across multiple charge states.

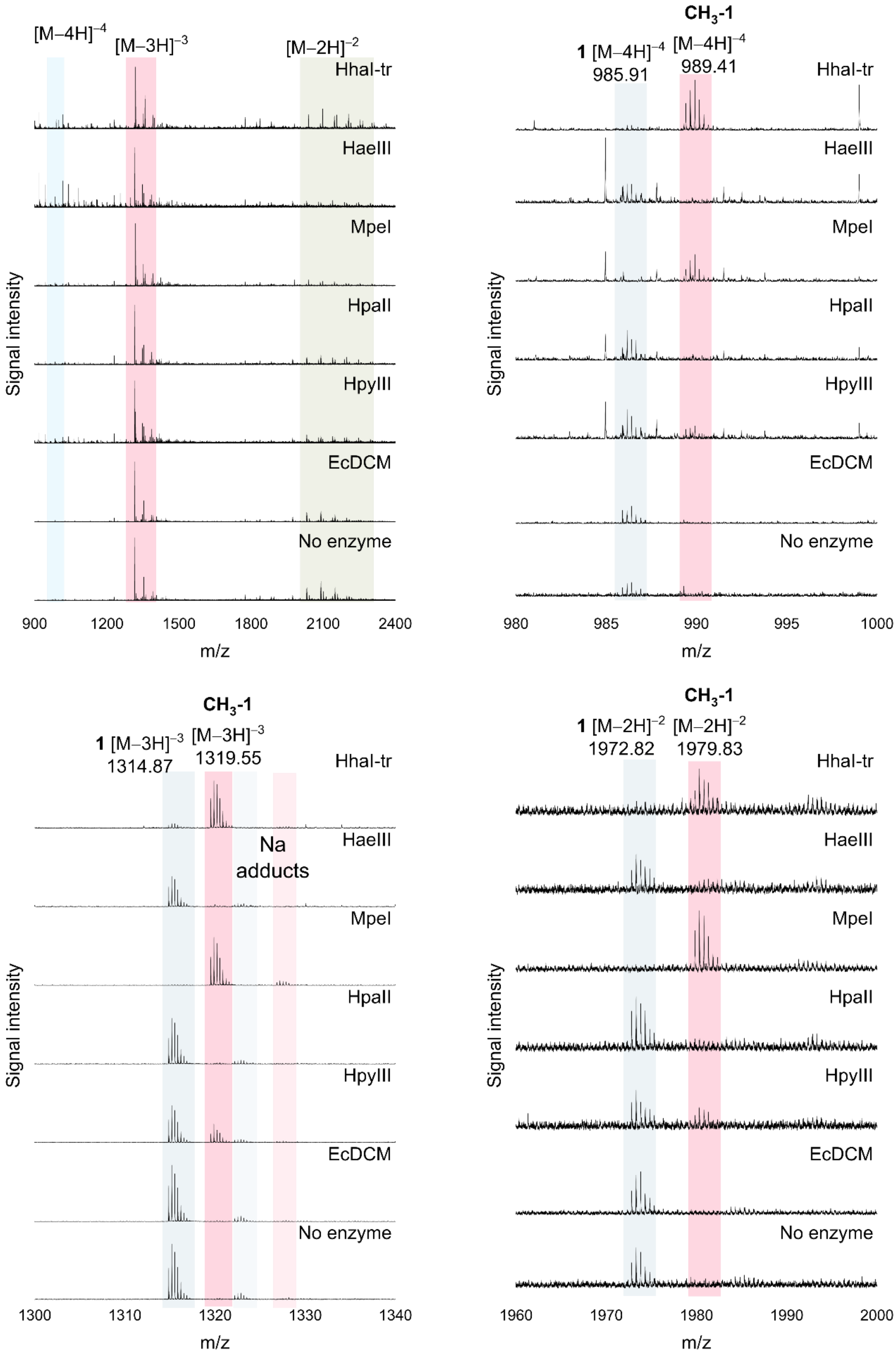

Figure S6: Example stacked raw HRMS spectra of biotransformations of substrate **1** in full view (top left), or focussed on the -4, -3 and -2 ion species (top right, bottom left and bottom right, respectively). The -3-charge state was by far the most prominent species observed. The raw

spectra annotated in this figure were used to generate maximum entropy deconvoluted spectra shown in Figure S8. The quantification of methylation was within 10% regardless of the different charge state, adduct or deconvoluted monoisotopic mass peaks used. For clarity in all of the following spectra, the deconvoluted mass spectra, or only the most prominent charged species are shown.

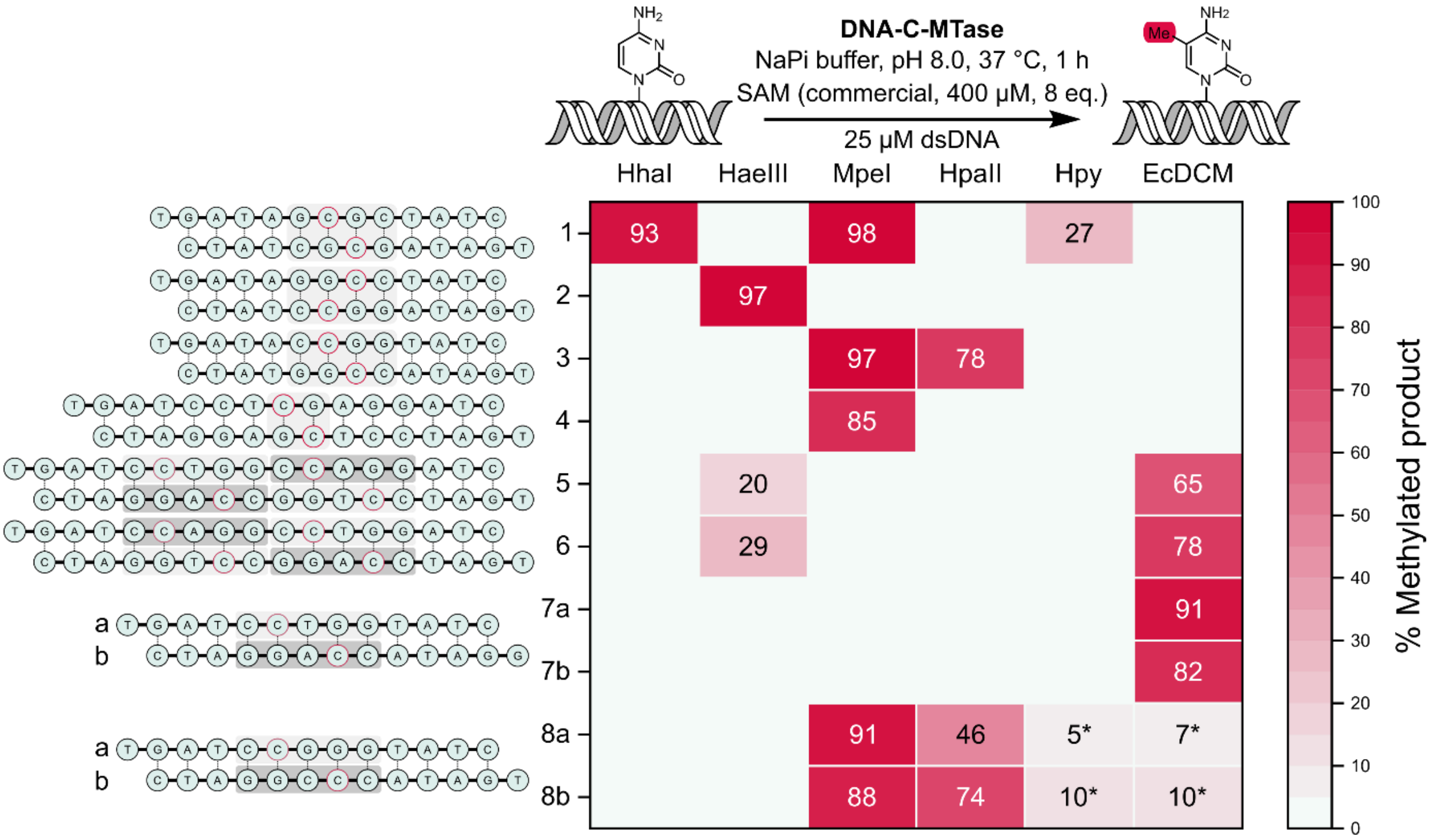

Figure S7: Percent methylated oligonucleotide detected after screening of DNA methyltransferases by HR-ESI-MS against substrates containing cognate recognition sites. Blank cells indicate less than 5% methylation and * indicates activity against a substrate not containing a cognate methylation site of that enzyme.

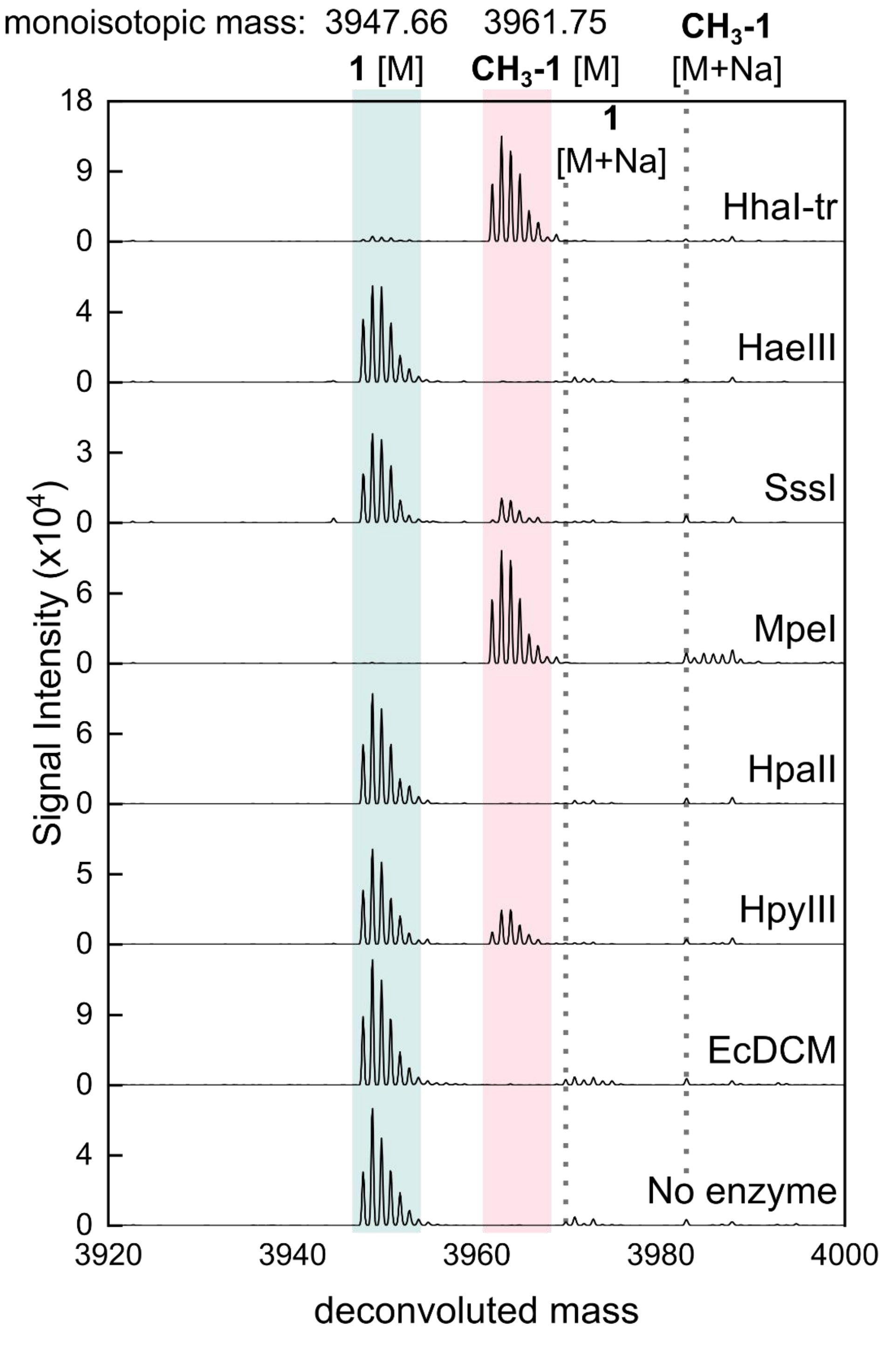

Figure S8: Stacked deconvoluted HR-ESI-MS data from screening biotransformations of oligonucleotide **1**.

Calculated monoisotopic mass **1** [M]: 3947.6979, observed: 3947.66 (mass error 9.6 ppm). Calculated monoisotopic mass **CH_3-1** [M]: 3961.7135, observed: 3961.75 (mass error 9.2 ppm).

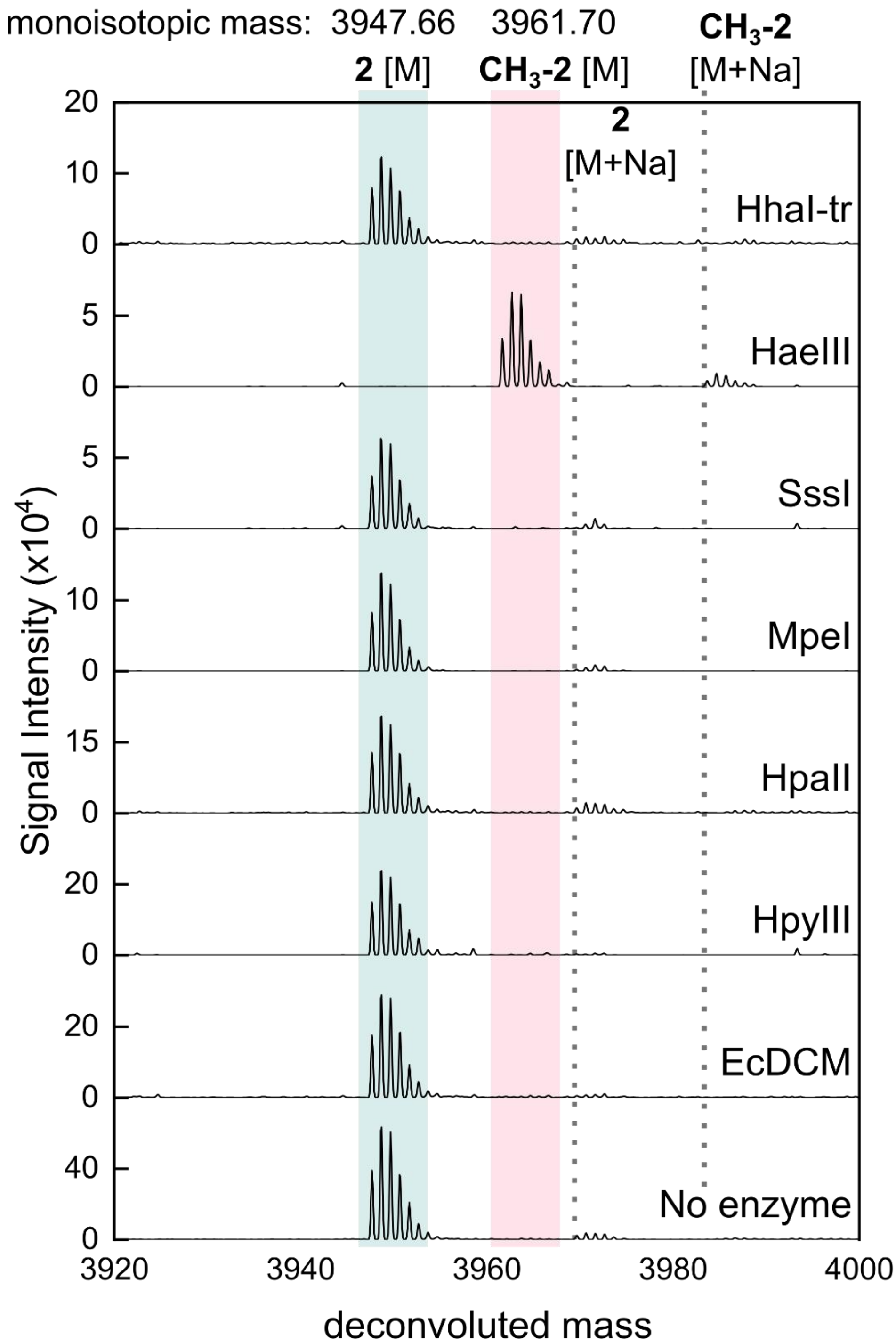

Figure S9: Stacked deconvoluted HR-ESI-MS data from screening biotransformations of oligonucleotide **2**.

Calculated monoisotopic mass **2** [M]: 3947.6979, observed: 3947.66 (mass error 9.6 ppm). Calculated monoisotopic mass **CH_3-2** [M]: 3961.7135, observed: 3961.70 (mass error 3.4 ppm).

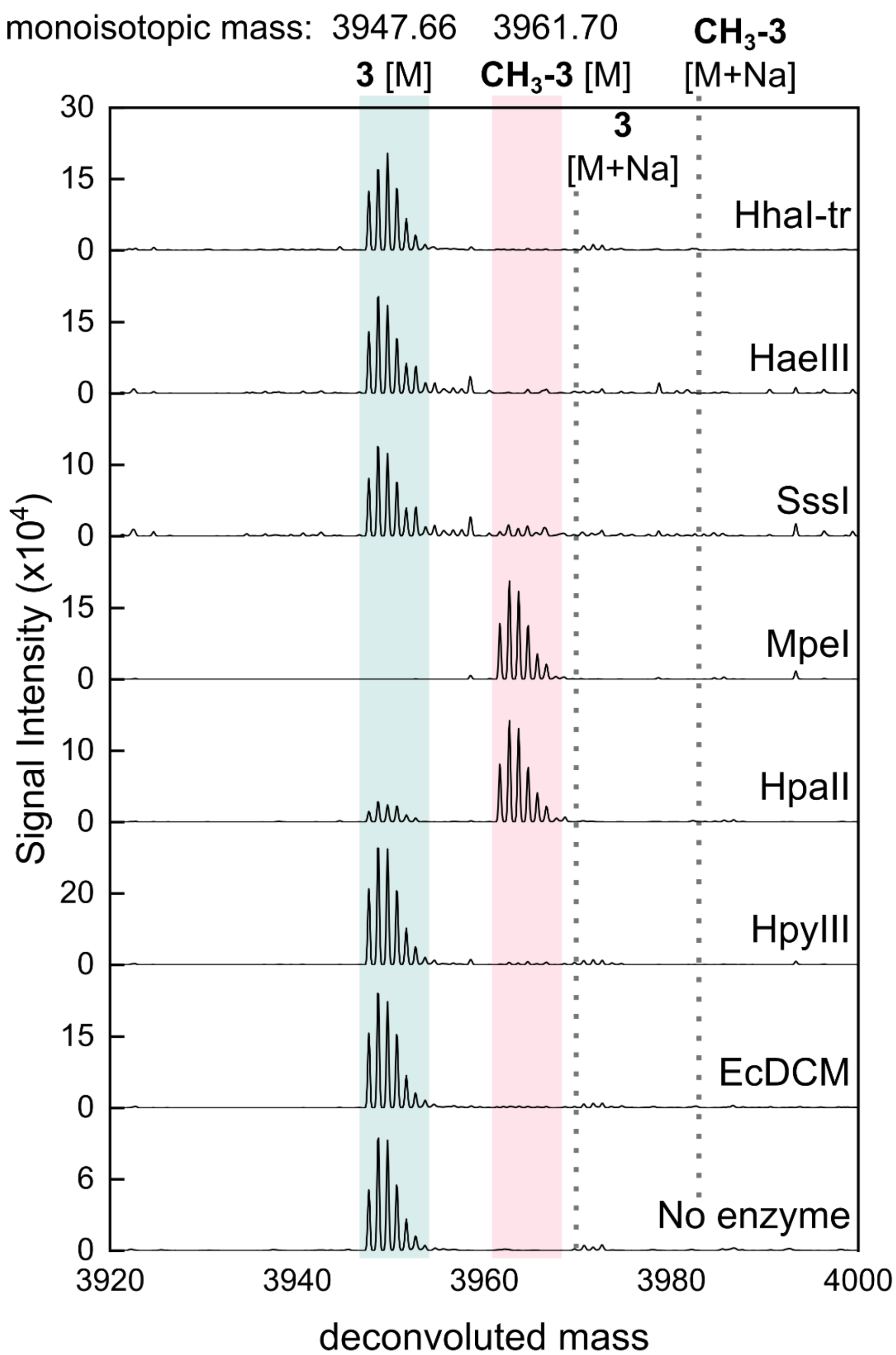

Figure S10: Stacked deconvoluted HR-ESI-MS data from screening biotransformations of oligonucleotide **3**.

Calculated monoisotopic mass **3** [M]: 3947.6979, observed: 3947.66 (mass error 9.6 ppm). Calculated monoisotopic mass **CH_3-3** [M]: 3961.7135, observed: 3961.70 (mass error 3.4 ppm).

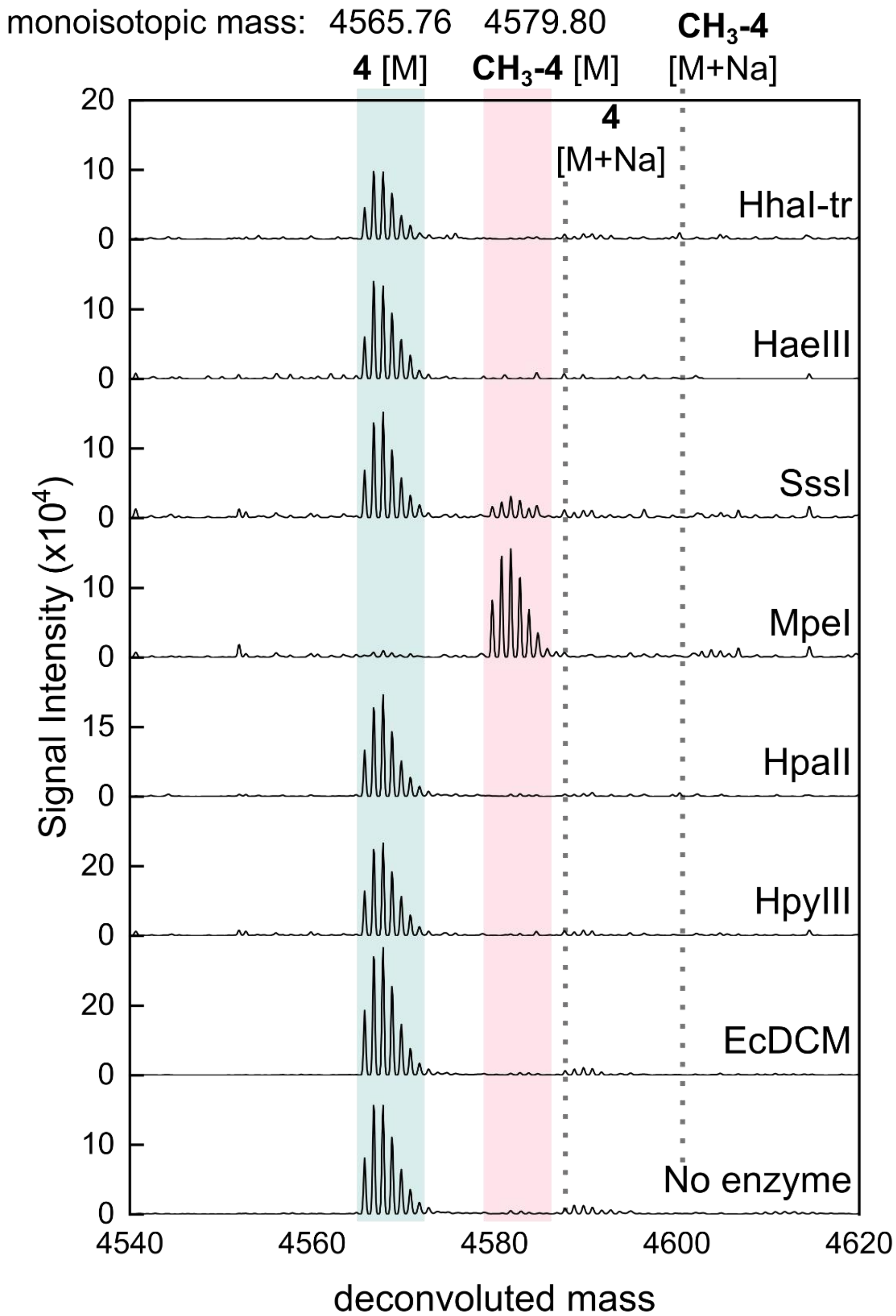

Figure S11: Stacked deconvoluted HR-ESI-MS data from screening biotransformations of oligonucleotide **4**.

Calculated monoisotopic mass **4** [M]: 4565.7968, observed: 4565.76 (mass error 8.1 ppm). Calculated monoisotopic mass **CH_3-4** [M]: 4579.8124, observed: 4579.80 (mass error 2.7 ppm).

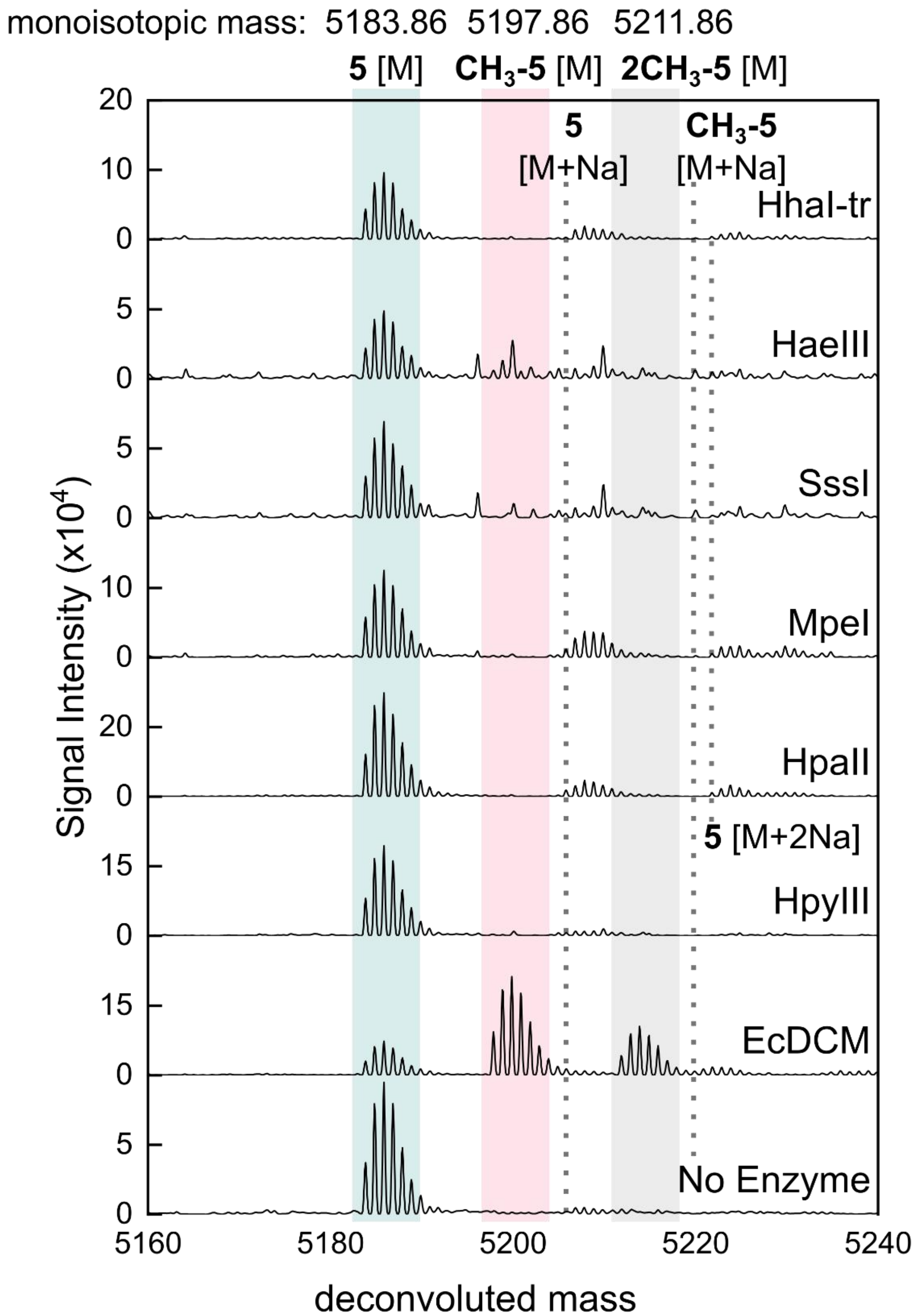

Figure S12: Stacked deconvoluted HR-ESI-MS data from screening biotransformations of oligonucleotide **5**.

Calculated monoisotopic mass **5** [M]: 5183.8957, observed: 5183.86 (mass error 6.8 ppm). Calculated monoisotopic mass **CH_3-5** [M]: 5197.9113, observed: 5197.86 (mass error 9.9 ppm). Calculated monoisotopic mass **$2CH_3$-5** [M]: 5211.9230, observed: 5211.86 (mass error 12 ppm).

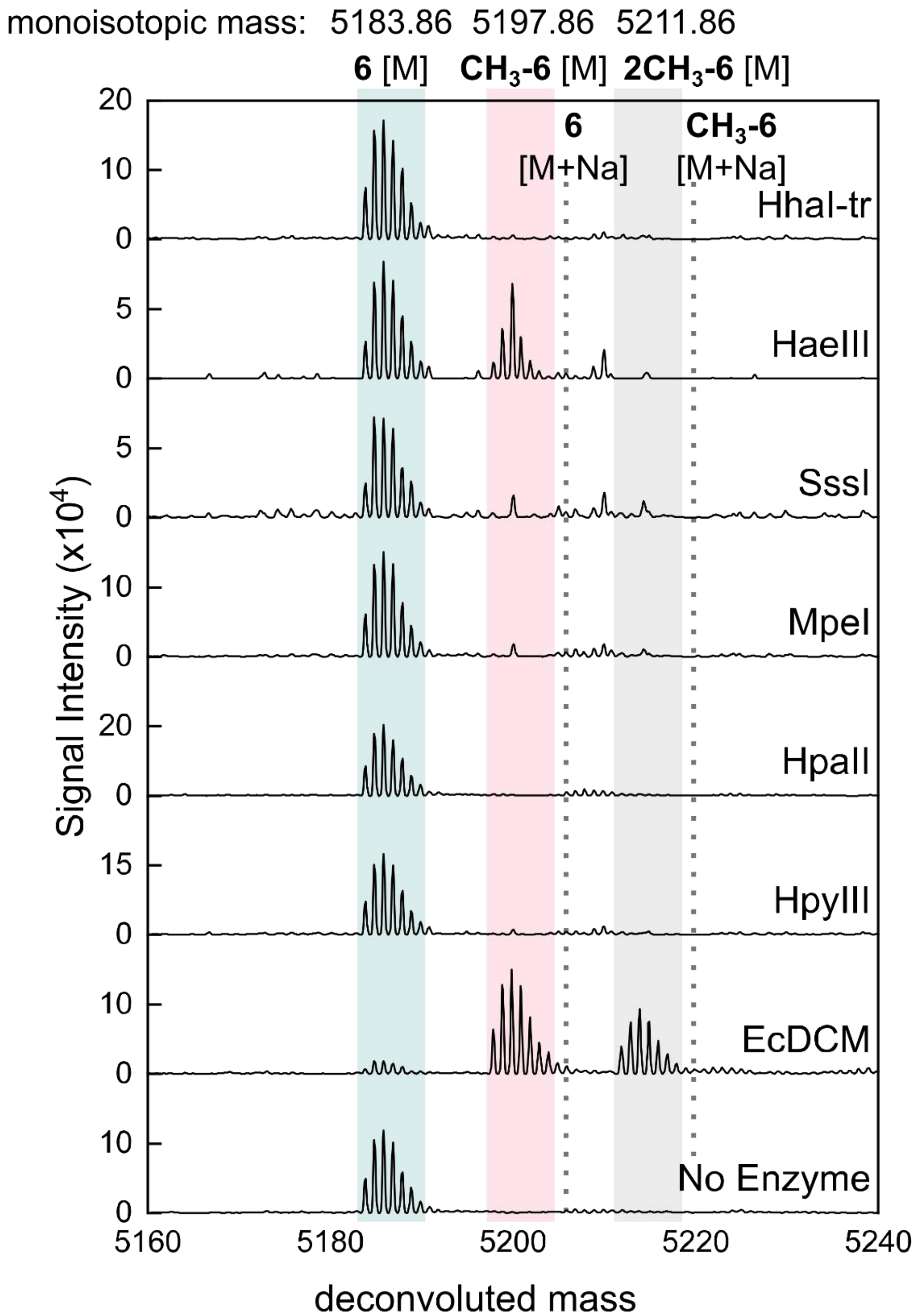

Figure S13: Stacked deconvoluted HR-ESI-MS data from screening biotransformations of oligonucleotide **6**.

Calculated monoisotopic mass **6** [M]: 5183.8957, observed: 5183.86 (mass error 6.8 ppm). Calculated monoisotopic mass **CH_3-6** [M]: 5197.9113, observed: 5197.86 (mass error 9.9 ppm). Calculated monoisotopic mass **2CH_3-6** [M]: 5211.9230, observed: 5211.86 (mass error 12 ppm).

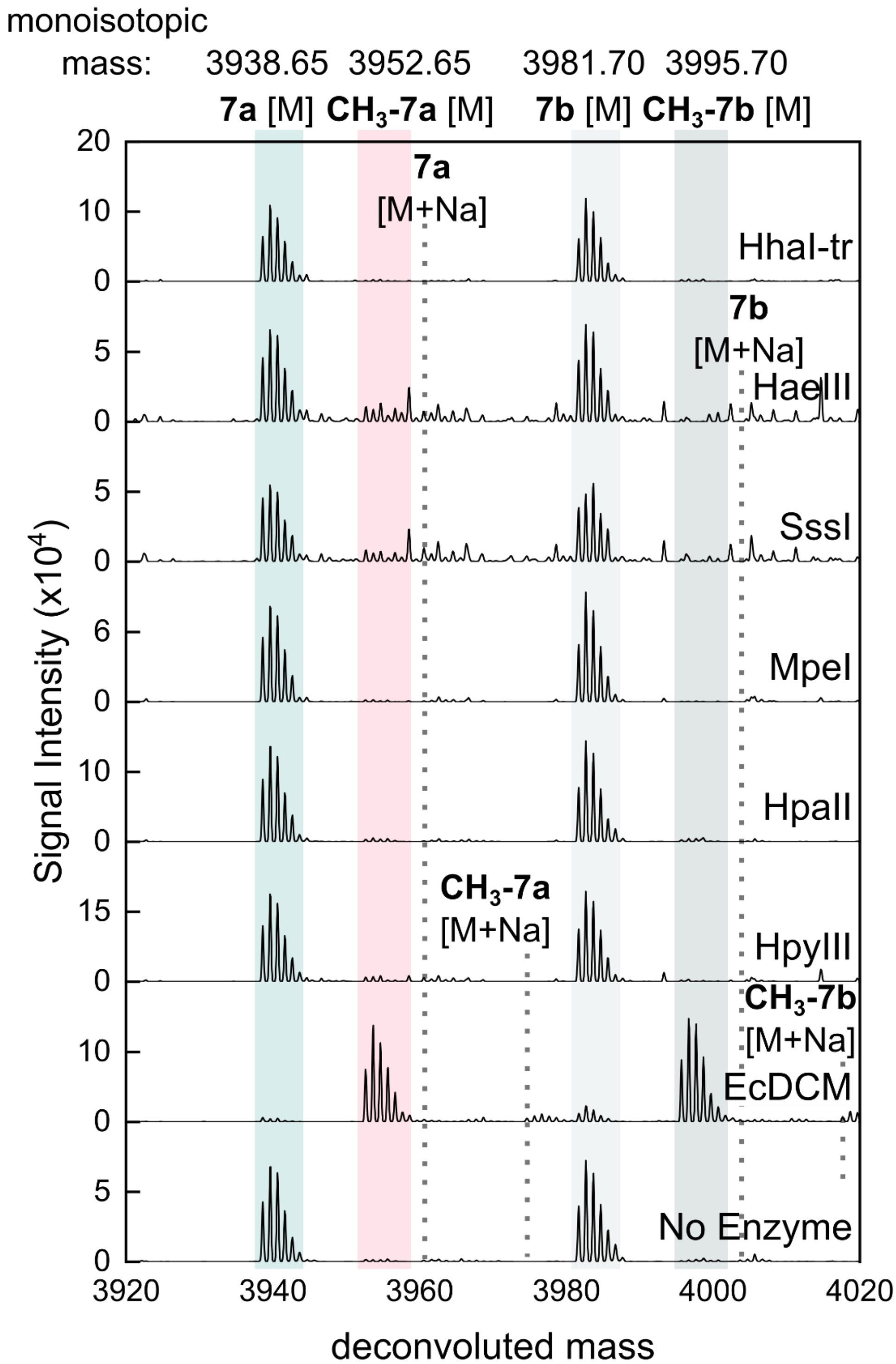

Figure S14: Stacked deconvoluted HR-ESI-MS data from screening biotransformations of oligonucleotide **7**.

Calculated monoisotopic mass **7a** [M]: 3938.6863, observed: 3938.65 (mass error 9.2 ppm). Calculated monoisotopic mass **CH_3-7a** [M]: 3952.7020, observed: 3938.65 (mass error 13 ppm). Calculated monoisotopic mass **7b** [M]: 3981.7159, observed: 3981.70 (mass error 4.0 ppm). Calculated monoisotopic mass **CH_3-7b** [M]: 3995.7316, observed: 3995.70 (mass error 7.9 ppm).

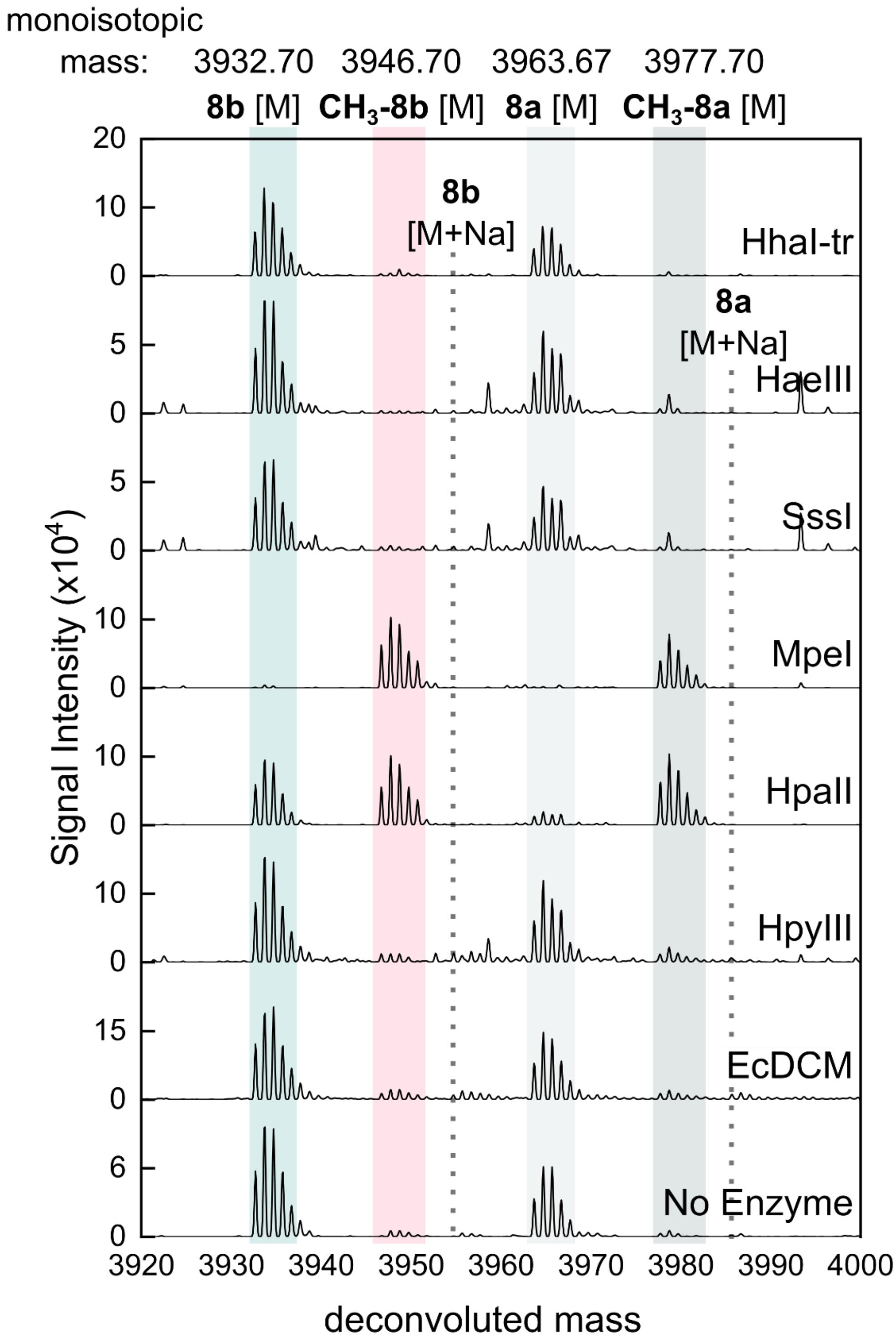

Figure S15: Stacked deconvoluted HR-ESI-MS data from screening biotransformations of oligonucleotide **8**.

Calculated monoisotopic mass **8a** [M]: 3963.6928, observed: 3963.67 (mass error 5.7 ppm). Calculated monoisotopic mass **CH_3-8a** [M]: 3977.7084, observed: 3977.70 (mass error 2.1 ppm). Calculated monoisotopic mass **8b** [M]: 3932.6982, observed: 3932.70 (mass error 0.45 ppm). Calculated monoisotopic mass **CH_3-8b** [M]: 3946.7139, observed: 3946.70 (mass error 3.5 ppm).

HMT reaction condition screening and MTAN activity confirmation

Reactions were performed in 1.5 mL Eppendorf tubes with a 200 µL reaction volume and contained: **HMT** (10 µM), SAH (1 mM), MeONs (1.1, 1.5 or 2 mM, from a 100x concentrated stock in MeCN or toluene) and NaPi buffer (50 mM, pH 8.0). The reactions were incubated at 25 °C, 400 rpm. 10 µL of the reaction mixture was taken and quenched with 1% v/v TFA in MeCN (10 µL), centrifuged at 15,000 x g for 10 minutes and analysed by IEX-HPLC at 20-, 40-and 60-minute timepoints as outlined in 'Materials and Methods'.

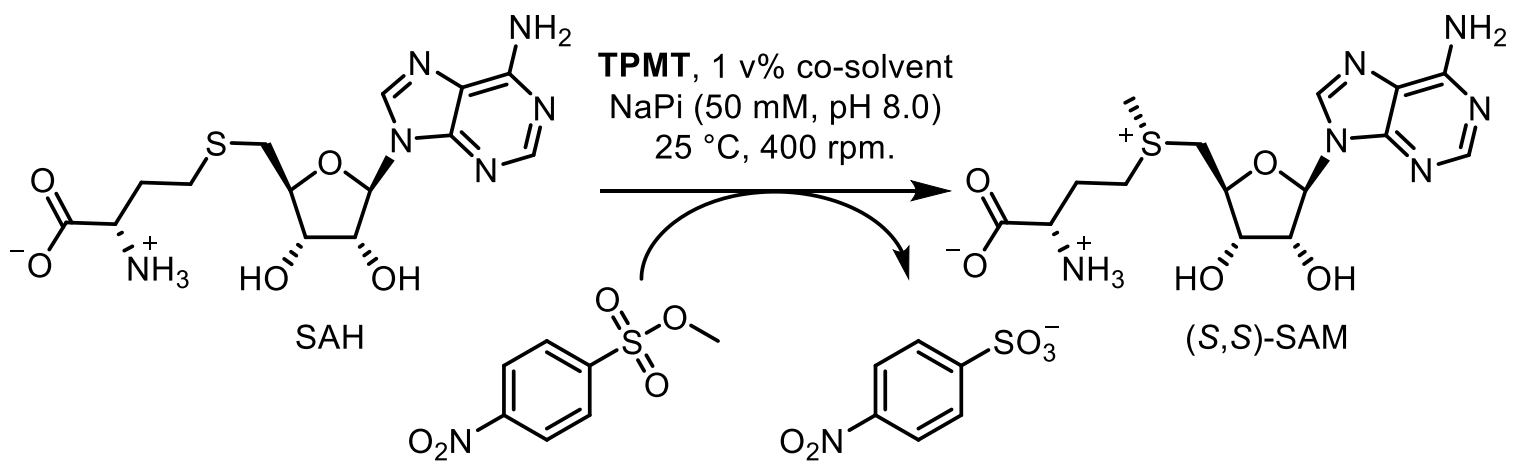

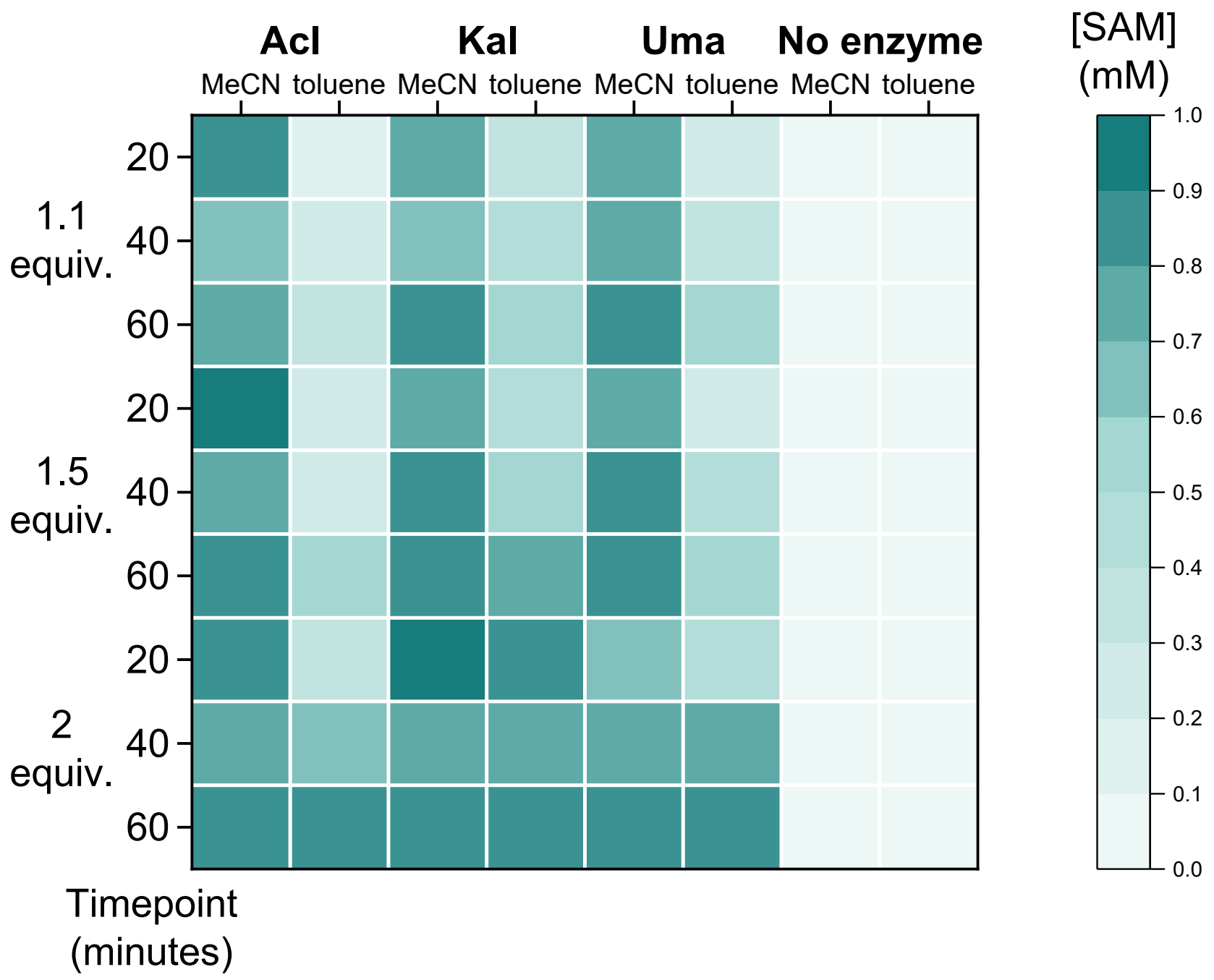

Figure S16: HMT assay to investigate the formation of SAM with varying equivalents of MeONs (1.1, 1.5 or 2) and co-solvent (MeCN or toluene) with three HMT enzymes (**Acl**, **Kal** and **Uma**) and a no enzyme control. Reactions conditions: **HMT** (10 µM), SAH (1 mM), MeONs (1.1, 1.5 or 2 mM, from a 100x concentrated stock in MeCN or toluene) and NaPi buffer (50 mM, pH 8.0). The reactions were incubated at 25 °C, 400 rpm and analysed by IEX-HPLC at 20-, 40-and 60-minute timepoints.

With efficient formation of SAM observed with only 1.1 equiv. of MeONs, a further experiment using MeONs as the limiting reagent was performed. The excess of SAH used would not interfere with downstream reactions as SAH is degraded by supplementation of reactions with MTAN.

Formation of SAM with limiting [MeONs]: Reactions were performed with a volume of 200 µL in 1.5 mL Eppendorf tubes and contained; **HMT** (***Uma, Acl*** or ***Kal***, 10 µM), SAH (1.2 mM, from a 5 mM stock in 2.5 % v/v DMSO $_{(aq.)}$), MeONs (1 mM, from a 100 mM stock in MeCN) and NaPi buffer (50 mM, pH 8.0). The reactions were incubated in a thermomixer at 25 °C for 30 minutes and the SAM

concentration quantified by IEX-HPLC as described in "Materials and methods" (Figure S8). Generally, Uma catalysed reactions formed c. 0.95 mM SAM, with *Acl* and *Kal* each forming c. 0.85 mM SAM. *Uma* was taken forward for further studies.

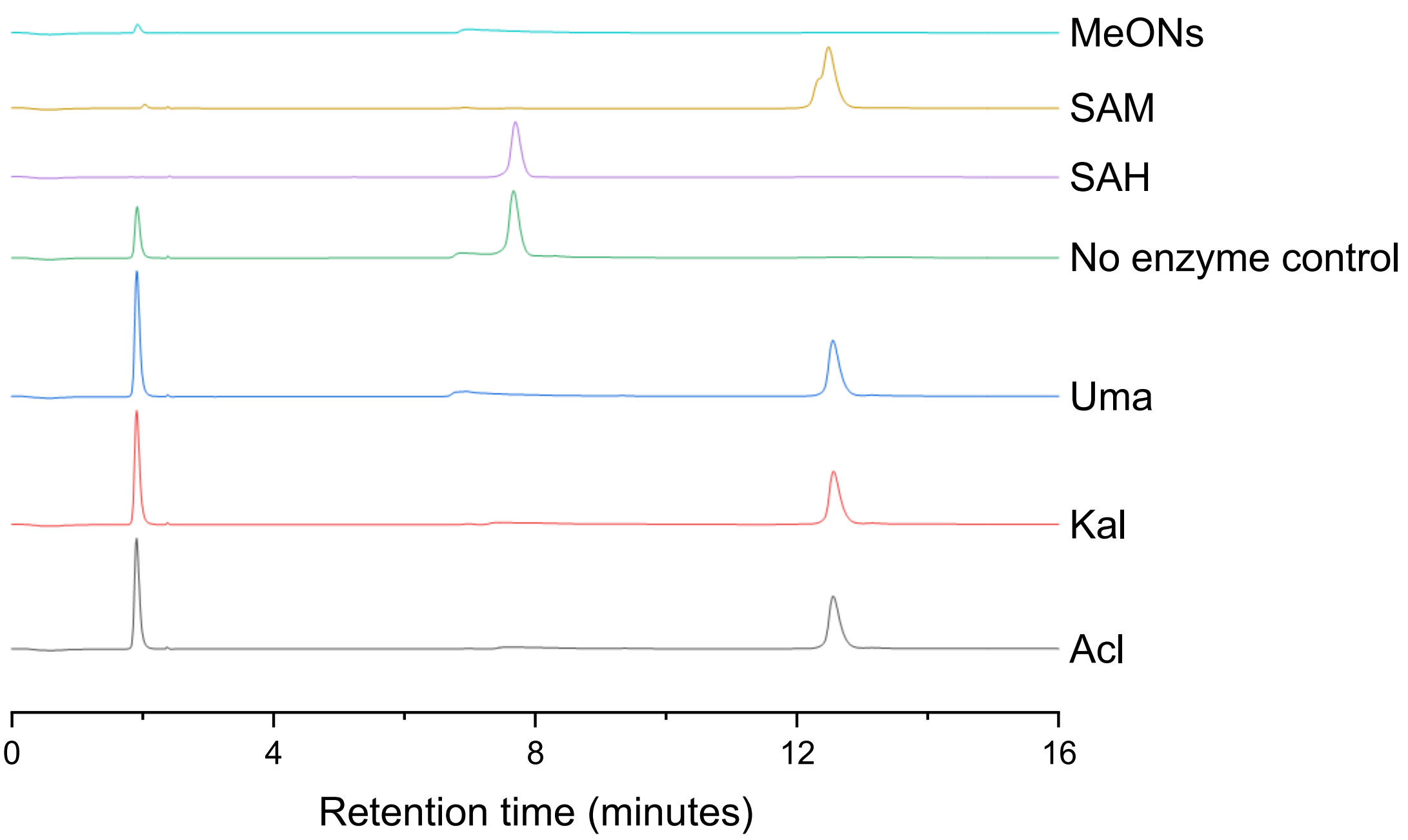

Figure S17: Stacked HPLC traces (UV 254 nm) from SAM formation biotransformations with MeONs as the limiting reagent and substrate and product standards MeONs, SAH and SAM (commercial, note the split peak corresponding to sulphonium epimerisation).

MTAN biotransformations were performed to confirm activity of the produced enzyme under the planned conditions of the DNA methylation reaction. Reactions were performed in 1.5 mL Eppendorf tubes with a total volume of 200 µL and contained: MTAN (1 µM) and SAH (reaction i: 1 mM or reaction ii: 0.1 mM) in NaPi buffer (50 mM, pH 8.0). The solutions were mixed at 37 °C, 400 rpm for 15 minutes. 10 µL of the reaction mixture was taken and quenched with 1 v% TFA in MeCN (10 µL), centrifuged at 15,000 x g for 10 minutes and analysed by IEX-HPLC as outlined in 'Materials and Methods'. Full depletion of SAH substrate (i: 1 mM and ii: 0.1 mM) was observed within 15 minutes in each biotransformation, accompanied by formation of adenine.

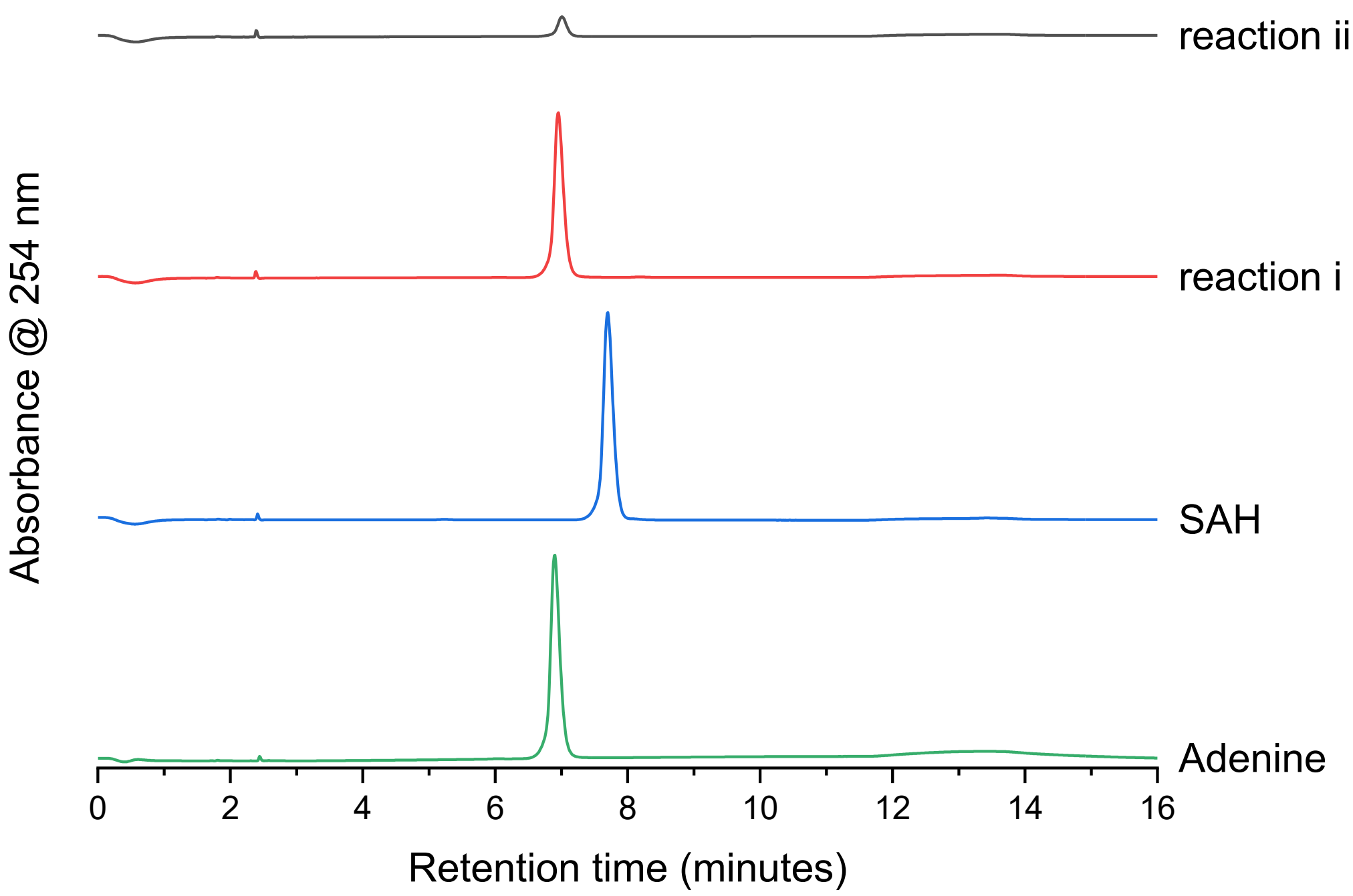

Figure S18: Stacked HPLC traces (UV 254 nm) from MTAN biotransformations and standards of SAH and adenine. Reactions i and ii contained 1 and 0.1 mM SAH, respectively. Full conversion to adenine was observed in both cases.

Enzymatic SAM formation and DNA methylation cascade

SAM formation. Reactions were generally performed with a volume of 200 µL in 1.5 mL Eppendorf tubes and contained: ***Uma*HMT** (10 µM), SAH (1.2 mM, from a 5 mM stock in 2.5% v/v DMSO $_{(aq.)}$), MeONs (1 mM, from a 100 mM stock in MeCN) and NaPi buffer (50 mM, pH 8.0). The reactions were incubated in a thermomixer at 25 °C for 30 minutes and the SAM concentration quantified by IEX-HPLC as described in "Materials and methods".

The remaining reaction mixture was used directly as a stock solution for the subsequent DNA methylation reaction without work up or purification.

DNA methylation. Typically, 10 µL reactions were performed in 8-well PCR tube strips or 96-well PCR plates and contained: **DNA-C5-MTase** (2 µM), ***Ec*MTAN** (2 µM), SAM (75 µM, as a crude preparation from the HMT reaction, 1.5 eq.), oligonucleotide substrate (50 µM single stranded basis), DTT (0.5 to 1 mM) and NaPi buffer (50 mM, pH 8.0). The reactions were incubated in a thermomixer or PCR thermocycler at 37 °C for 1 hour. The reaction was analysed by HRMS following DNA precipitation as described in "Materials and methods" and percent methylation was calculated. Analogous no enzyme control reactions were routinely performed by replacing the volume of enzyme stock with autoclaved MilliQ water.

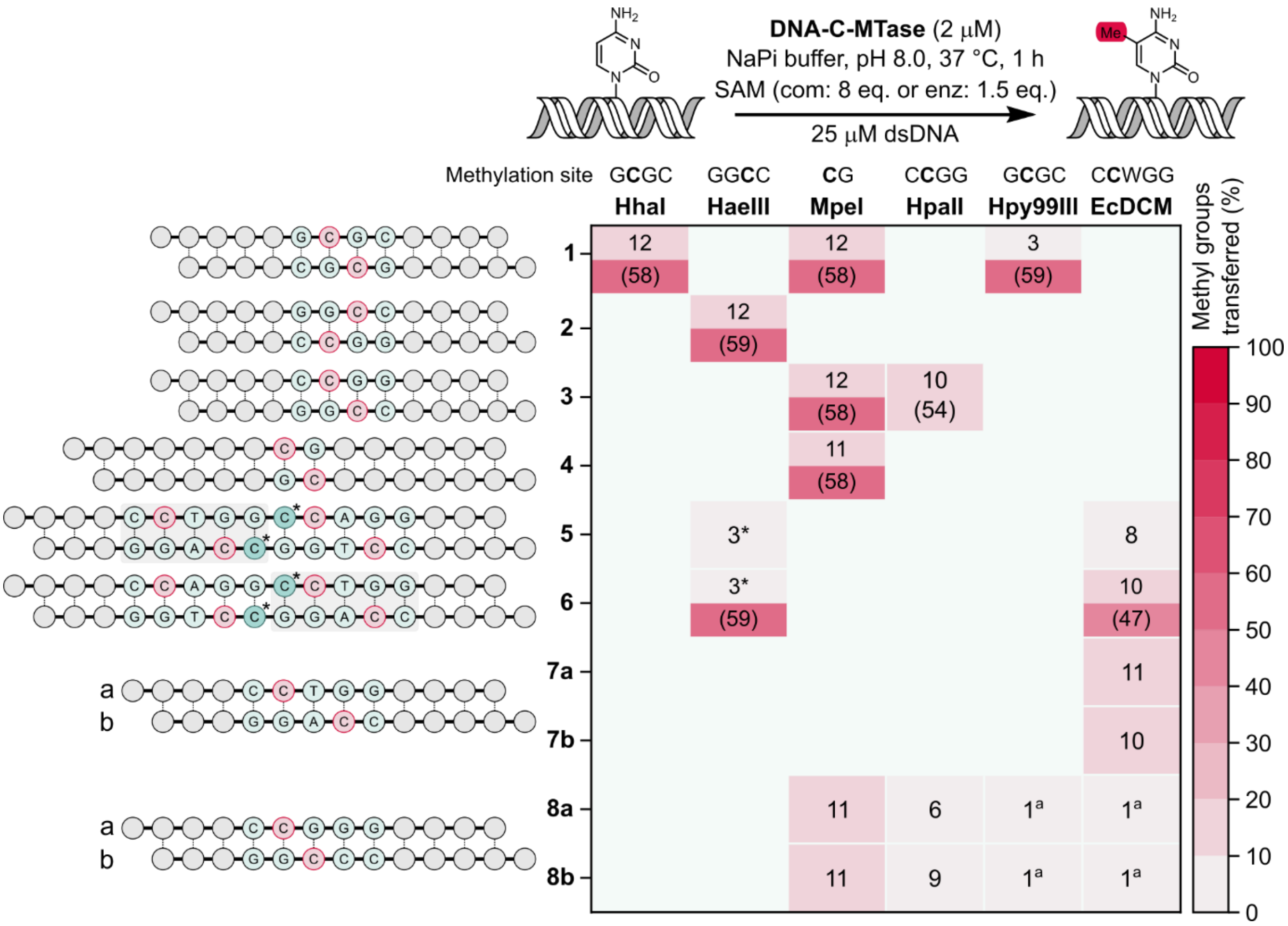

Figure S19: Combined heatmap of screening and optimised (data in brackets) DNA methylation reactions as a function of % of methyl groups transferred from the methyl source to oligonucleotide. These values are calculated as the amount of methylated oligonucleotide formed compared to the amount of SAM added to each reaction. For enzymatically formed SAM, this is corrected for the efficiency of SAM synthesis from MeONs (c. 95%). E.g., the theoretical maximum % methyl groups transferred in a biotransformation using 1.5 equivalents of SAM would be 66.67%. This metric informs the overall reaction efficiency with respect to the methyl group source.

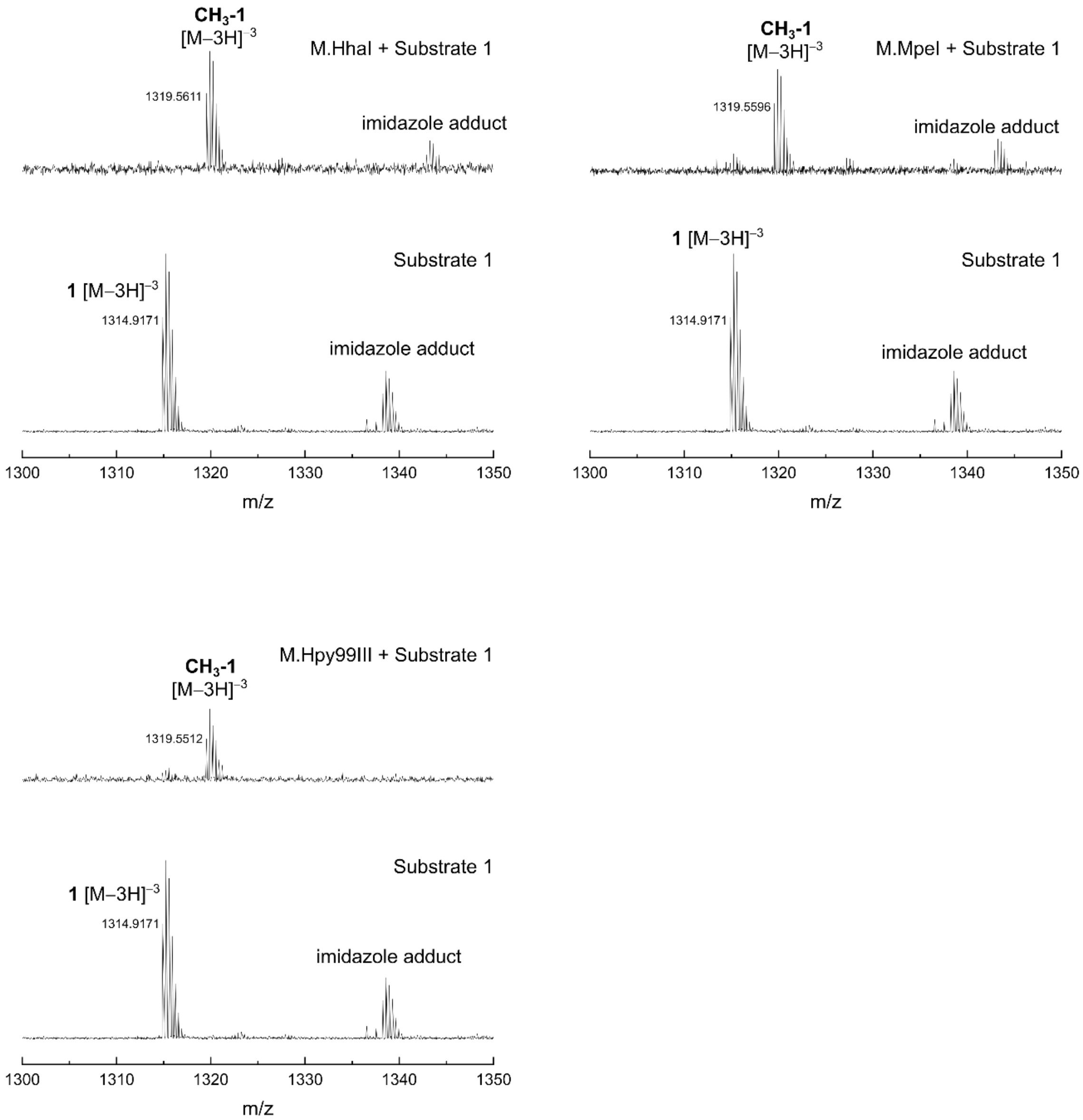

Figure S20: Stacked HR-ESI-MS spectra of oligonucleotide methylation biotransformations of substrate 1 with M.HhaI, M.MpeI and M.Hpy99III using enzymatically synthesised (*S*,*S*)-SAM. Plotted spectra are the raw MS data with multiply charged species. Here, the −3-charge state of oligonucleotide 1 and corresponding methylated oligonucleotide are shown. Additional mass envelopes correspond to imidazole adducts of each oligonucleotide.

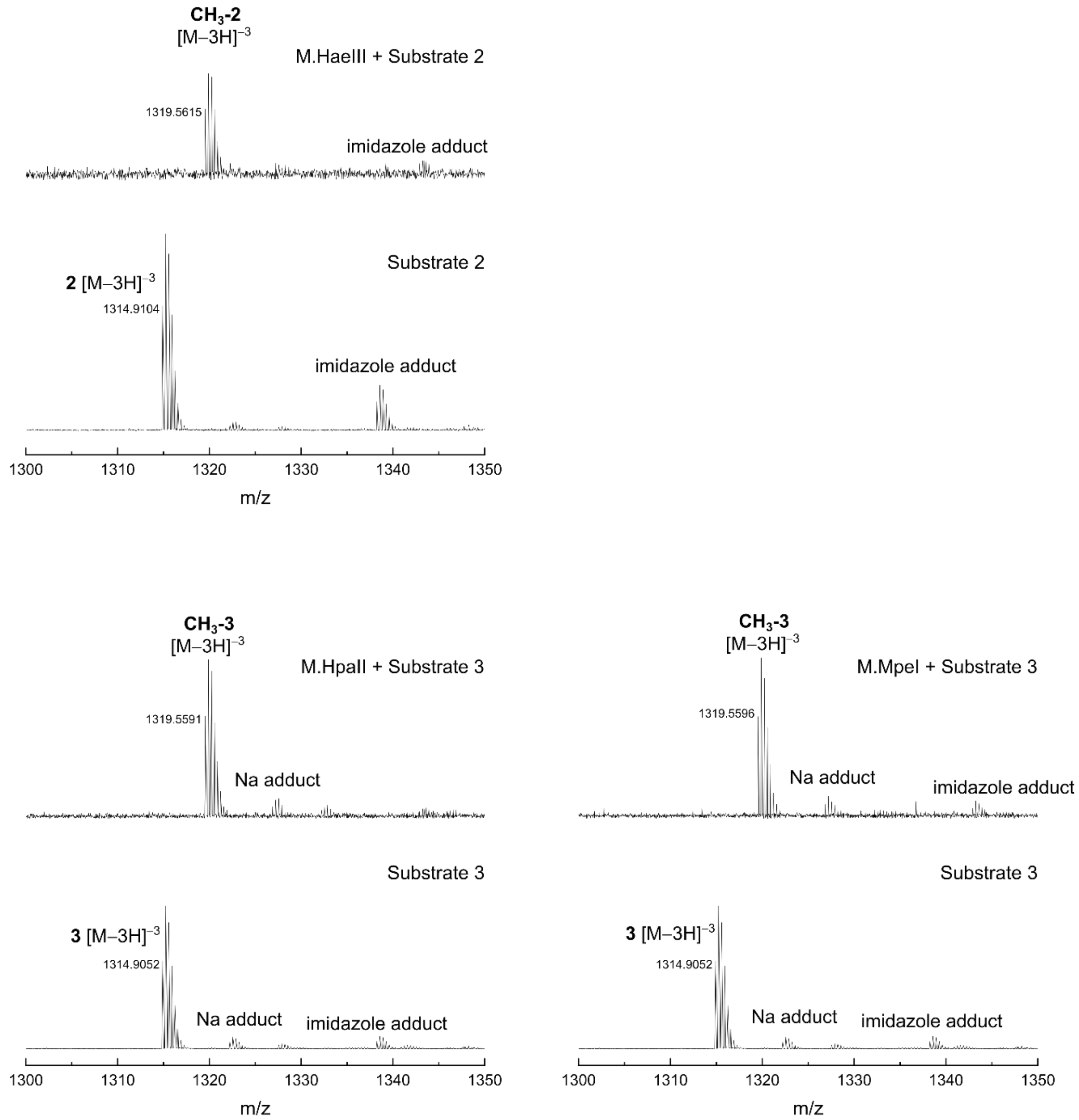

Figure S21: Stacked HR-ESI-MS spectra of oligonucleotide methylation biotransformations of substrate 2 with M.HaeIII and substrate 3 with M.MpeI and M.HpaII using enzymatically synthesised (*S*,*S*)-SAM. Plotted spectra are the raw MS data with multiply charged species. Here, the −3-charge state of each oligonucleotide 2 and 3 and corresponding methylated oligonucleotide are shown.

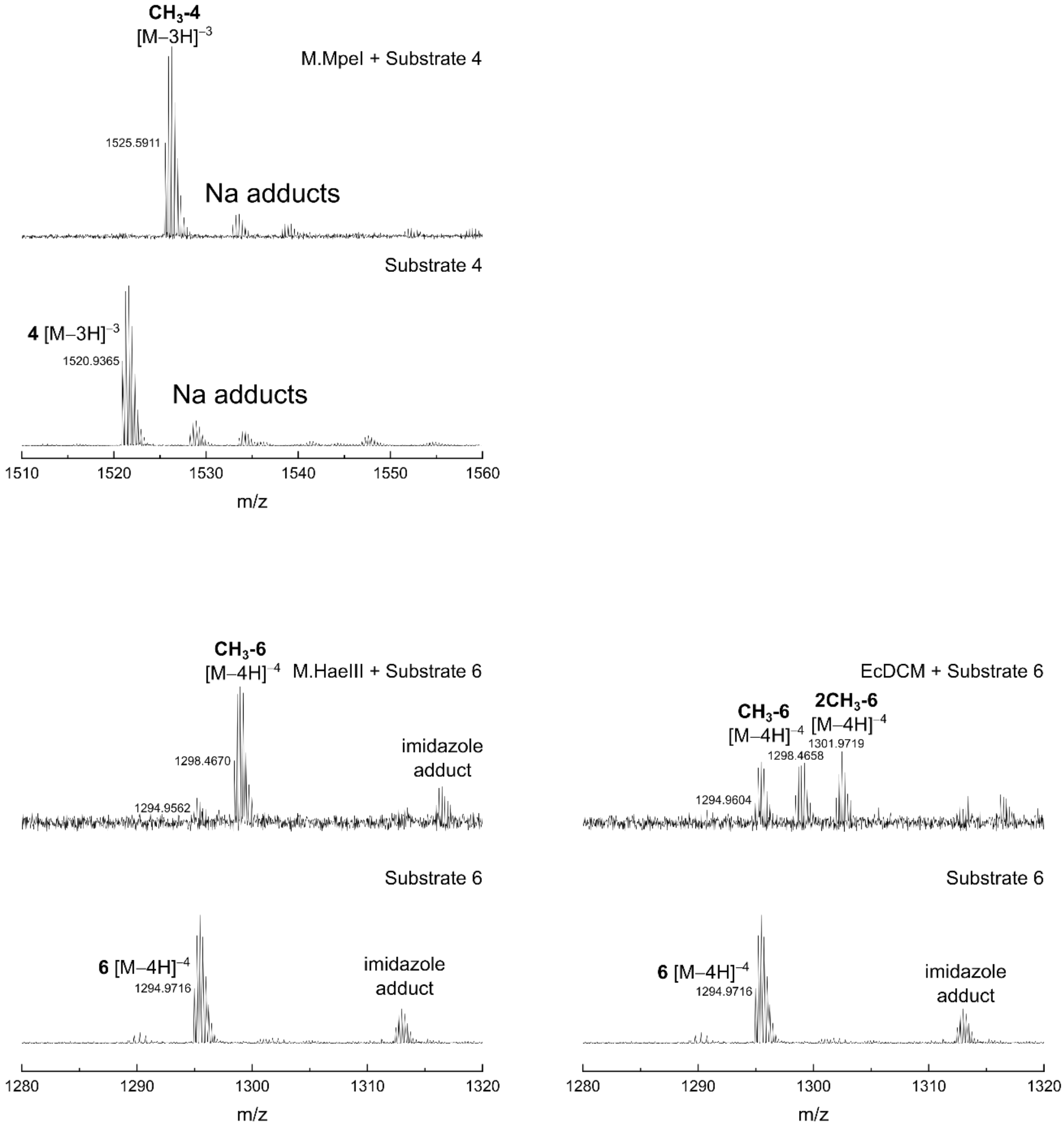

Figure S22: Stacked HR-ESI-MS spectra of oligonucleotide methylation biotransformations of substrate 4 with M.MpeI and substrate 6 with M.HaeIII and EcDCM using enzymatically synthesised (*S*,*S*)-SAM. Plotted spectra are the raw MS data with multiply charged species. Here, the −3-charge state of oligonucleotide 4 and −4-charge state of oligonucleotide 6 are shown, alongside their corresponding methylated oligonucleotides.

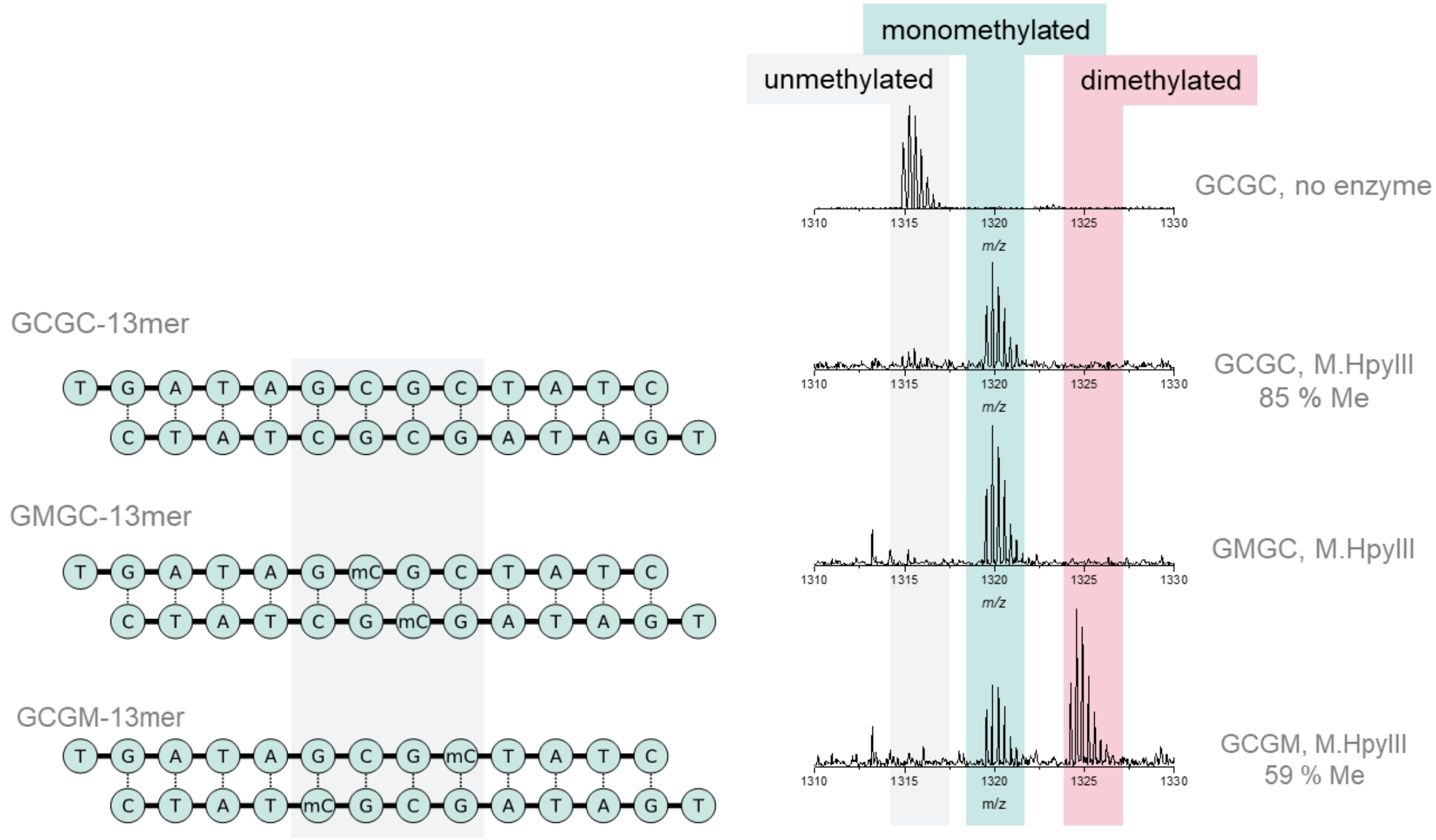

Figure S23: Confirmation of M.Hpy99III methylation site regioselectivity using pre-methylated substrate probes (based on substrate **1**) with cytosine or 5-methylcytosine (mC or M) in key positions. M.HpyIII methylated the oligonucleotide with internal 'GCGC' and 'GCGmC' motifs but not the substrate containing 'GmCGC', indicating that the internal cytosine of the G**C**GC motif is methylated regioselectively. These reactions were performed under the general conditions above using enzymatically synthesised (*S*,*S*)-SAM. The HRMS data shown correspond to the $[M-3H]^{3-}$ species of the unmethylated (grey, left), monomethylated (blue, middle) and dimethylated (red, right) forms of oligonucleotide **1**, respectively.

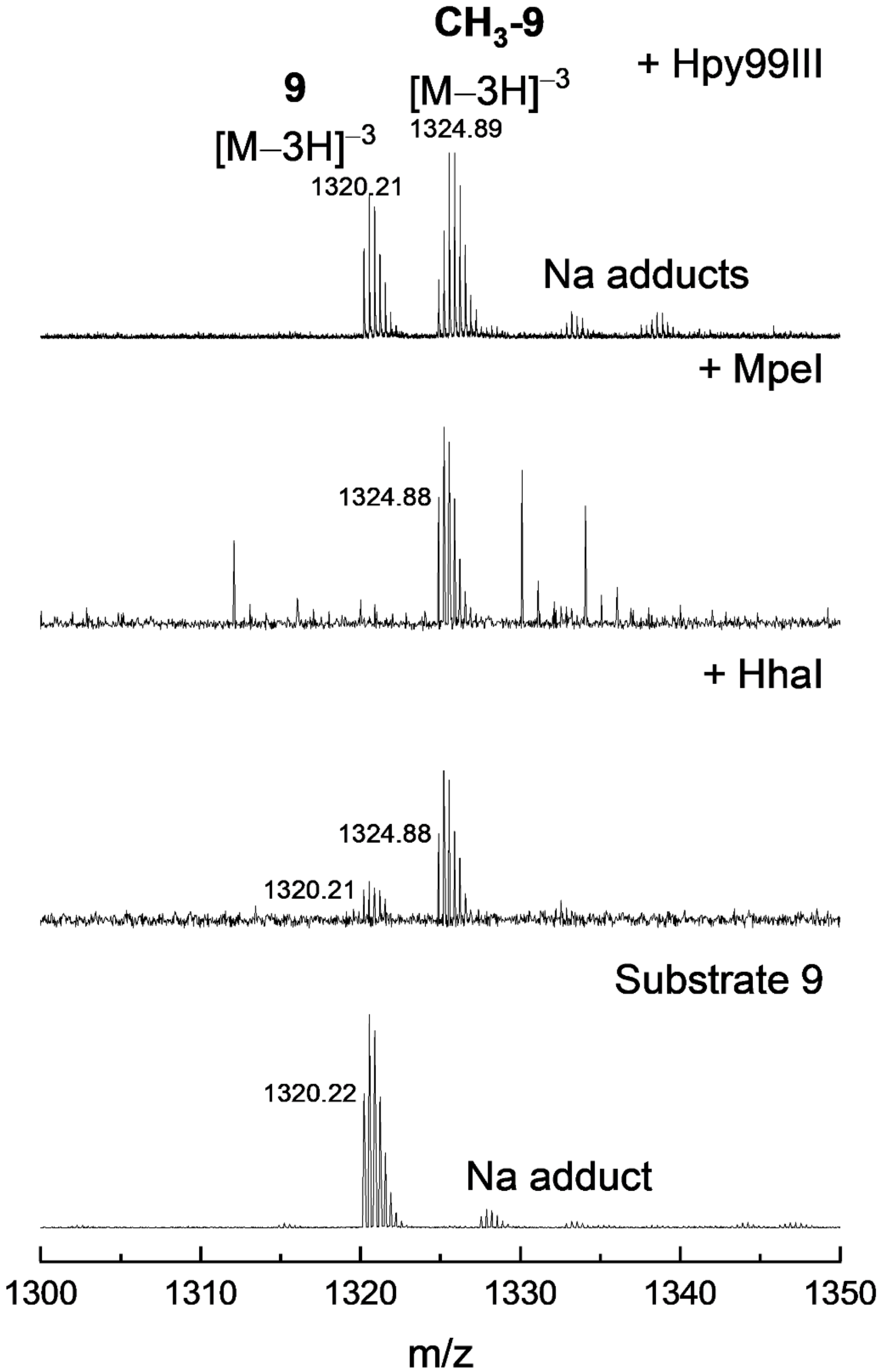

Figure S24: Stacked HR-ESI-MS spectra of oligonucleotide methylation biotransformations of substrate 9 with M.HhaI, M.MpeI and M.Hpy99III using enzymatically synthesised (*S*,*S*)-SAM. Plotted spectra are the raw MS data with multiply charged species. Here, the −3-charge state of oligonucleotide 9 alongside the corresponding methylated oligonucleotide is shown.

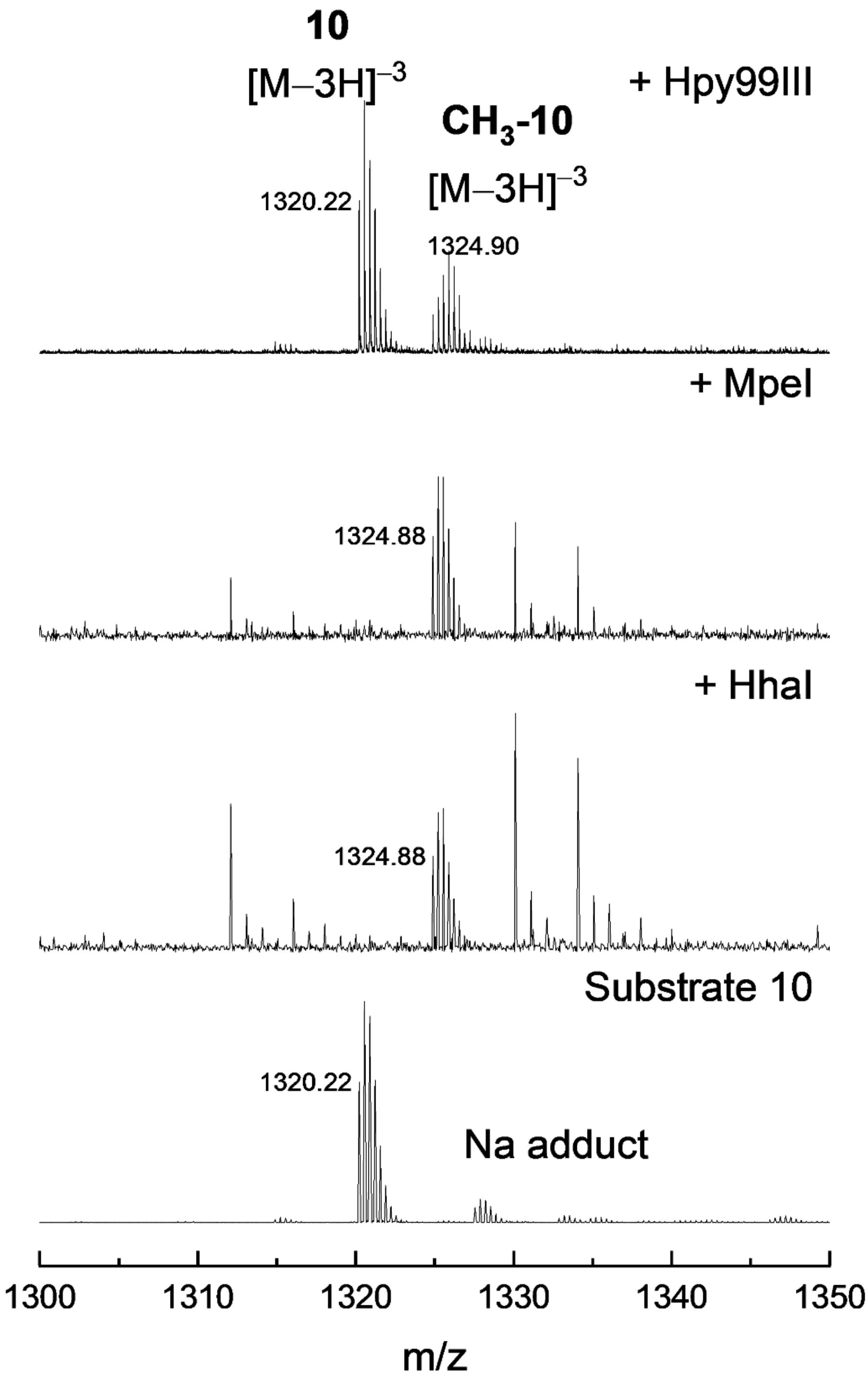

Figure S25: Stacked HR-ESI-MS spectra of oligonucleotide methylation biotransformations of substrate 10 with M.HhaI, M.MpeI and M.Hpy99III using enzymatically synthesised (*S*,*S*)-SAM. Plotted spectra are the raw MS data with multiply charged species. Here, the −3-charge state of oligonucleotide 10 alongside the corresponding methylated oligonucleotide is shown.

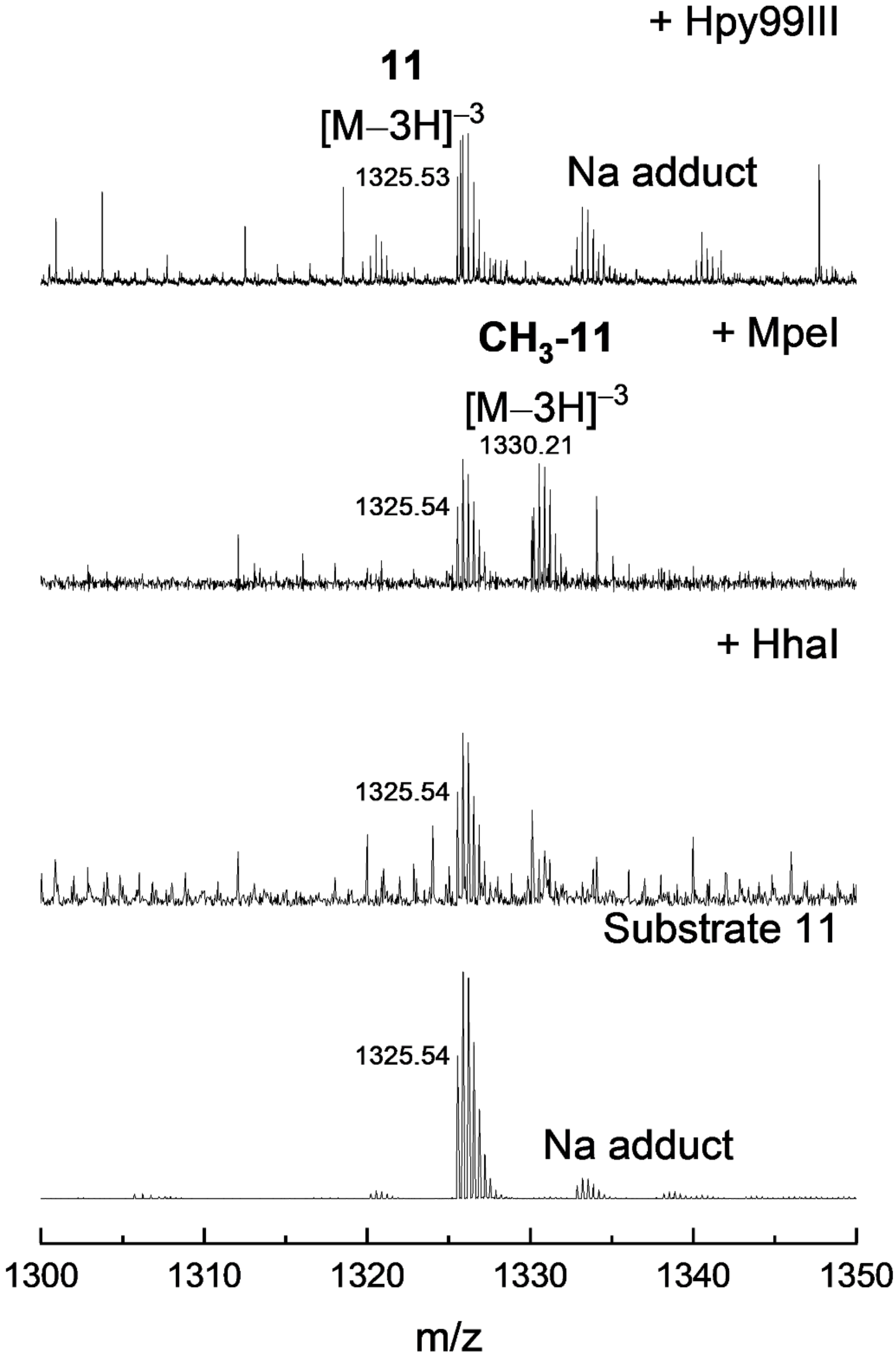

Figure S26: Stacked HR-ESI-MS spectra of oligonucleotide methylation biotransformations of substrate 11 with M.HhaI, M.MpeI and M.Hpy99III using enzymatically synthesised (*S*,*S*)-SAM. Plotted spectra are the raw MS data with multiply charged species. Here, the −3-charge state of oligonucleotide 11 alongside the corresponding methylated oligonucleotide is shown.

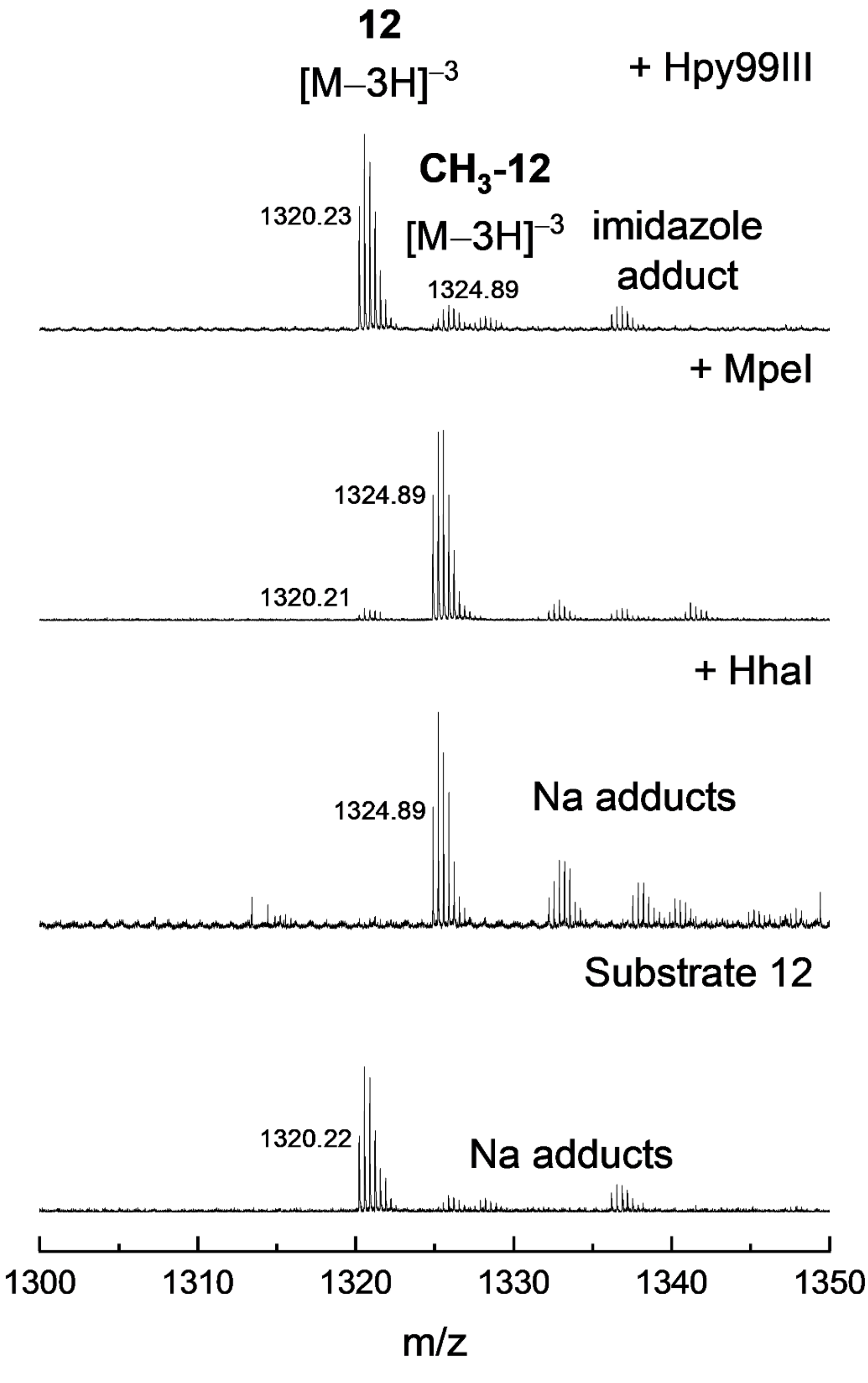

Figure S27: Stacked HR-ESI-MS spectra of oligonucleotide methylation biotransformations of substrate 12 with M.HhaI, M.MpeI and M.Hpy99III using enzymatically synthesised (*S*,*S*)-SAM. Plotted spectra are the raw MS data with multiply charged species. Here, the −3-charge state of oligonucleotide 13 alongside the corresponding methylated oligonucleotide is shown.

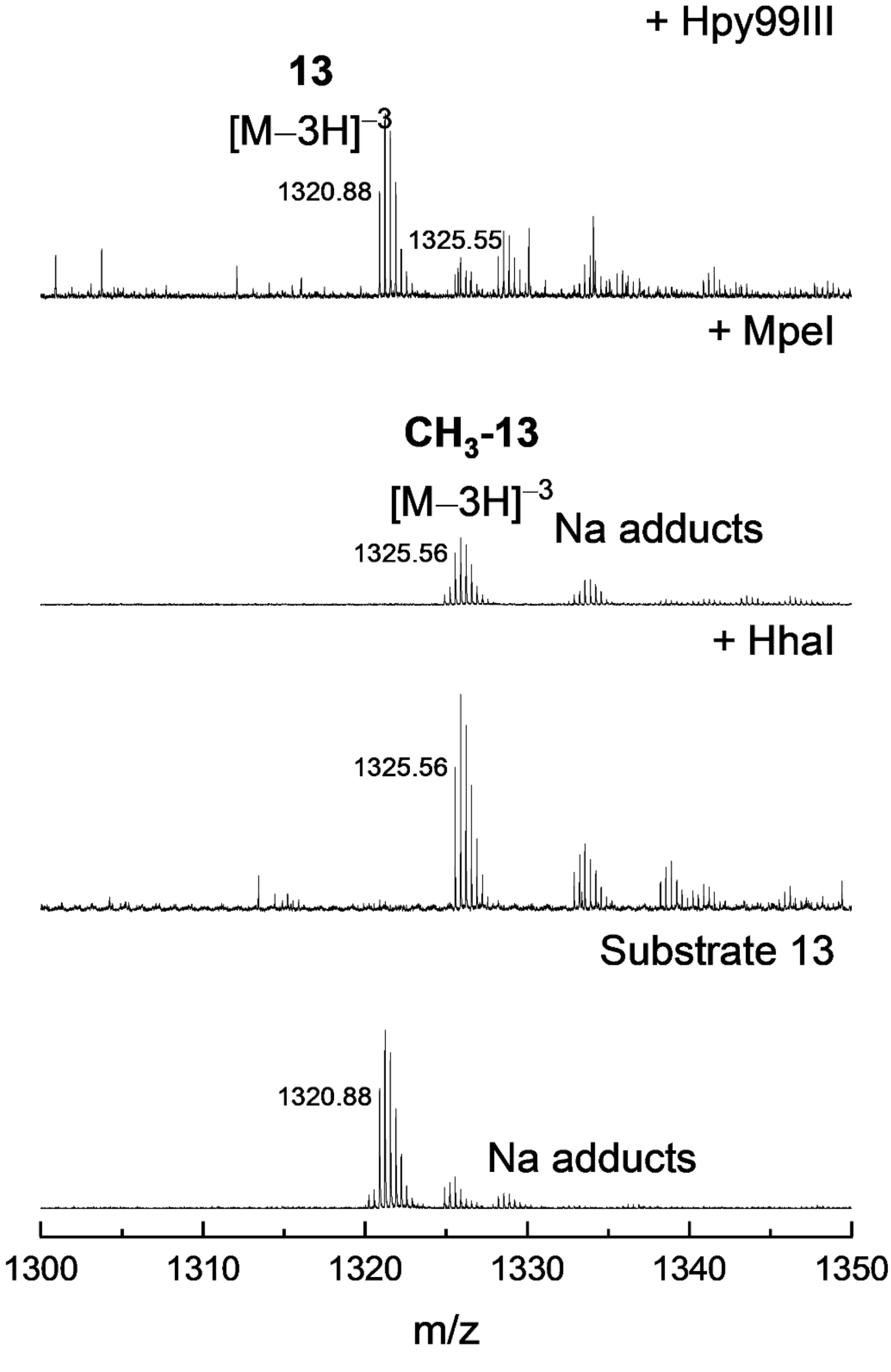

Figure S28: Stacked HR-ESI-MS spectra of oligonucleotide methylation biotransformations of substrate 13 with M.HhaI, M.MpeI and M.Hpy99III using enzymatically synthesised (*S*,*S*)-SAM. Plotted spectra are the raw MS data with multiply charged species. Here, the −3-charge state of oligonucleotide 13 alongside the corresponding methylated oligonucleotide is shown.

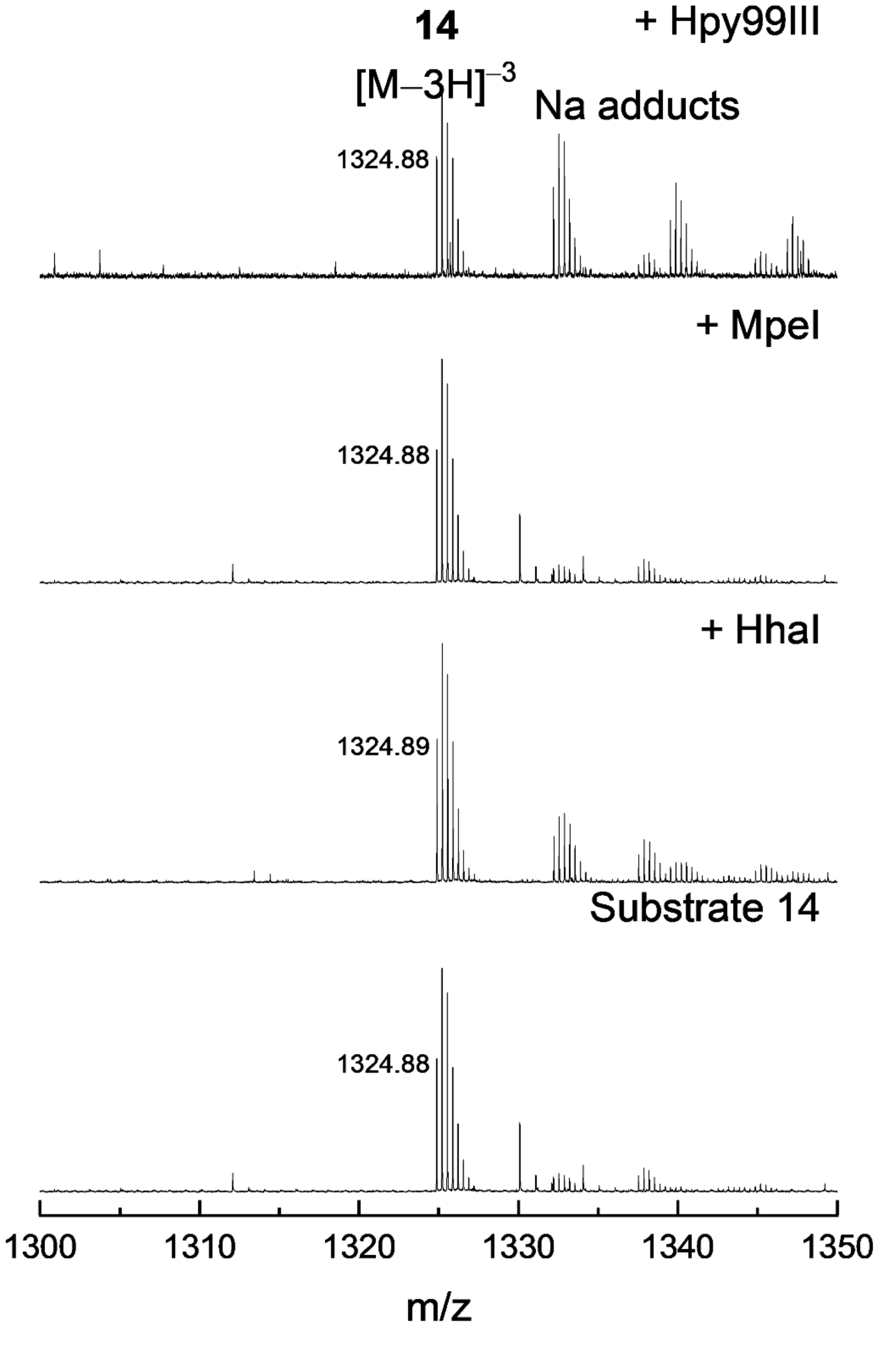

Figure S29: Stacked HR-ESI-MS spectra of oligonucleotide methylation biotransformations of substrate 14 with M.HhaI, M.MpeI and M.Hpy99III using enzymatically synthesised (*S*,*S*)-SAM. Plotted spectra are the raw MS data with multiply charged species. Here, the −3-charge state of oligonucleotide 14 alongside the corresponding methylated oligonucleotide is shown.

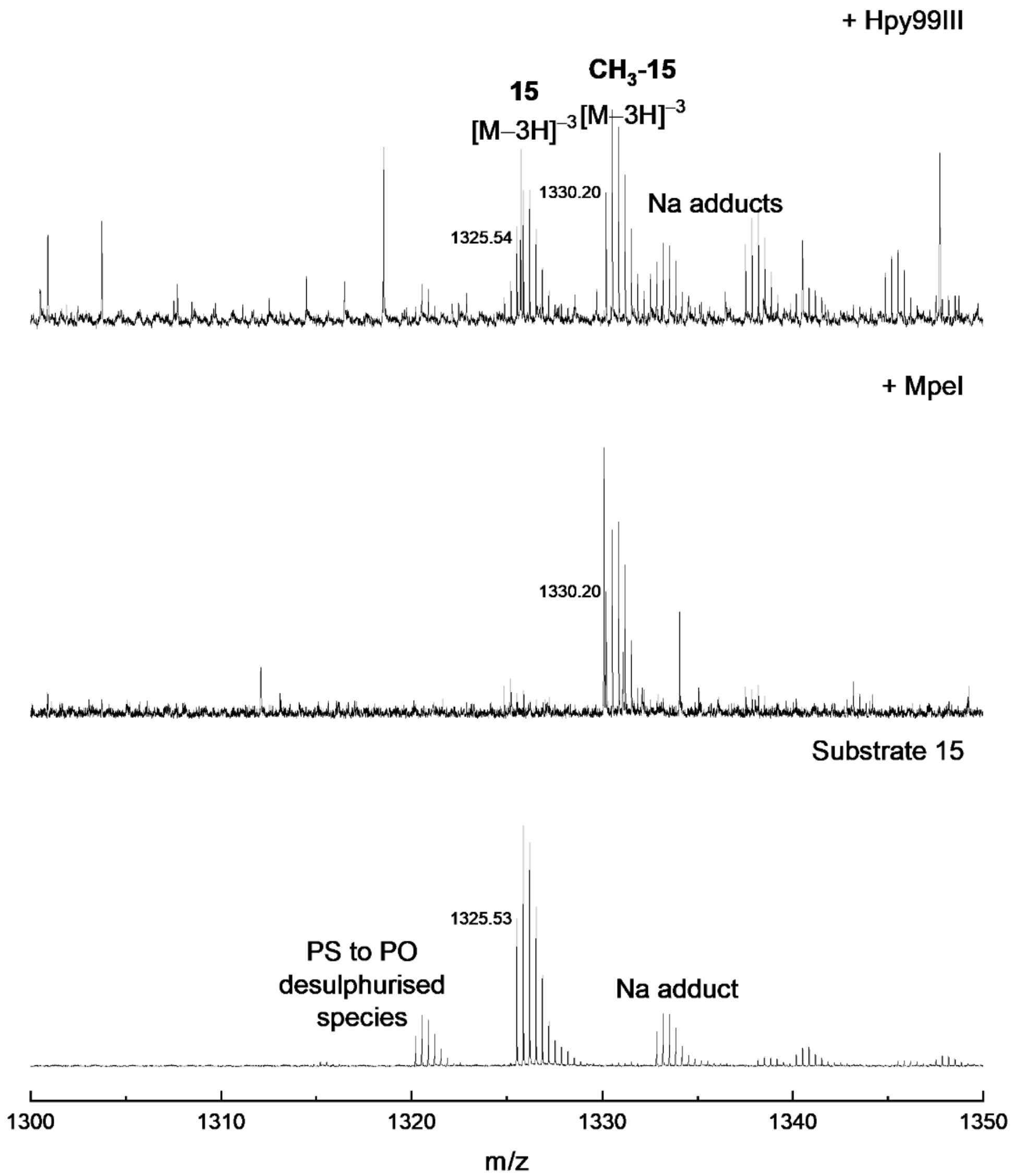

Figure S30: Stacked HR-ESI-MS spectra of oligonucleotide methylation biotransformations of substrate 15 with M.MpeI and M.Hpy99III using enzymatically synthesised (*S*,*S*)-SAM. Plotted spectra are the raw MS data with multiply charged species. Here, the −3-charge state of oligonucleotide 15 alongside the corresponding methylated oligonucleotide is shown.

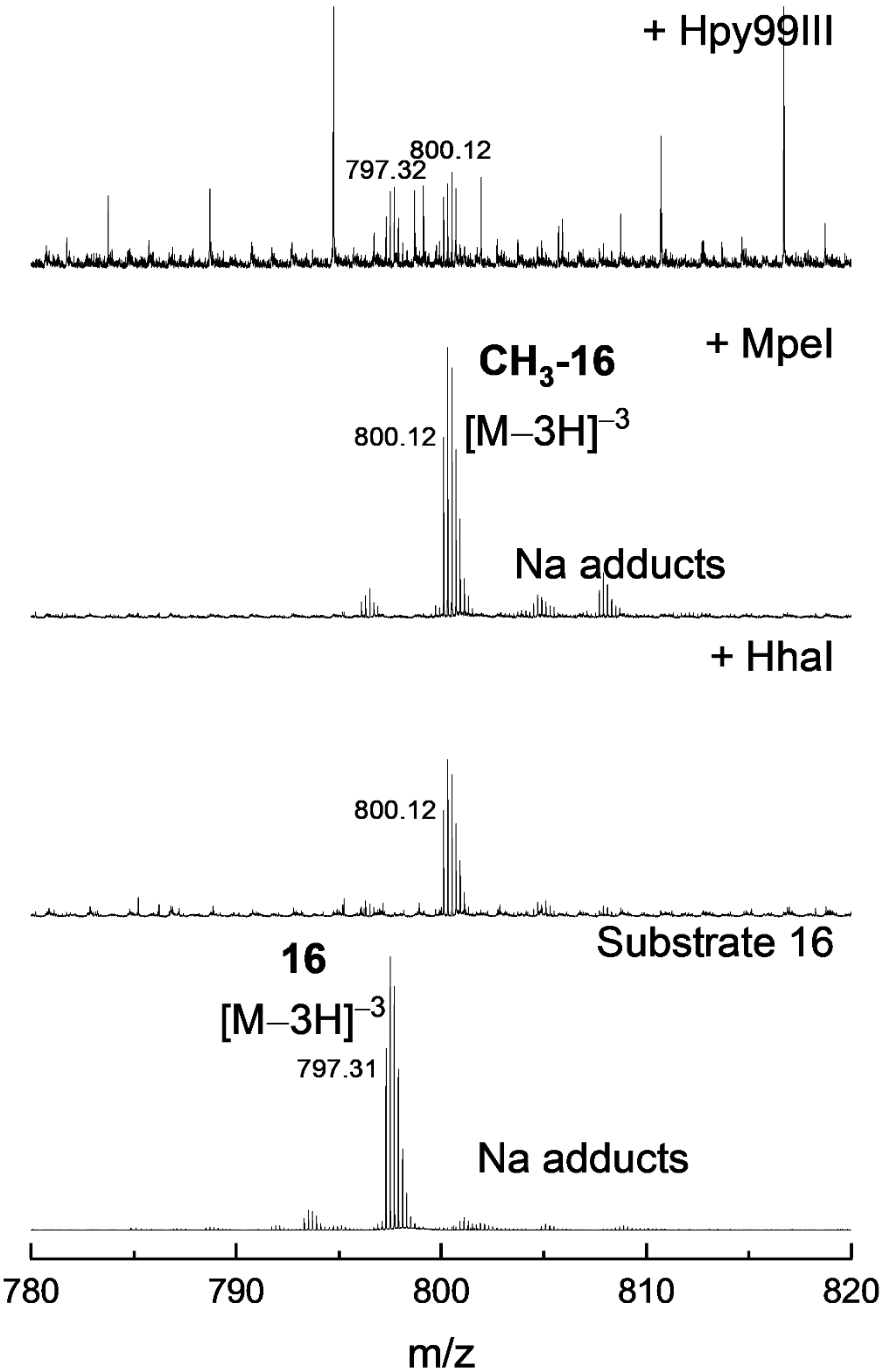

Figure S31: Stacked HR-ESI-MS spectra of oligonucleotide methylation biotransformations of substrate 16 with M.HhaI, M.MpeI and M.Hpy99III using enzymatically synthesised (*S*,*S*)-SAM. Plotted spectra are the raw MS data with multiply charged species. Here, the −5-charge state of oligonucleotide 16 alongside the corresponding methylated oligonucleotide is shown.

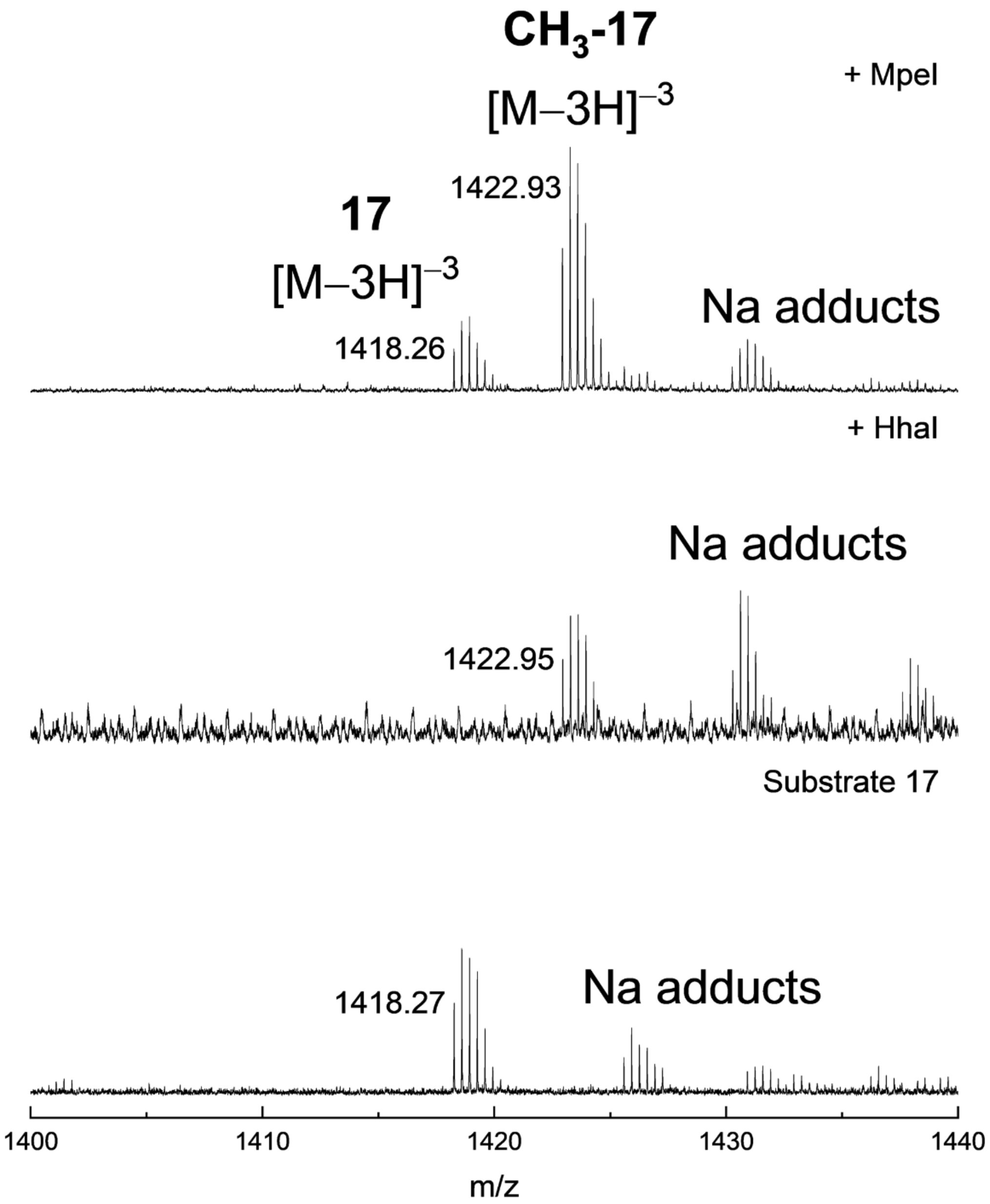

Figure S32: Stacked HR-ESI-MS spectra of oligonucleotide methylation biotransformations of substrate 17 with M.MpeI using enzymatically synthesised (*S*,*S*)-SAM. Plotted spectra are the raw MS data with multiply charged species. Here, the −4-charge state of oligonucleotide 17 alongside the corresponding methylated oligonucleotide is shown.

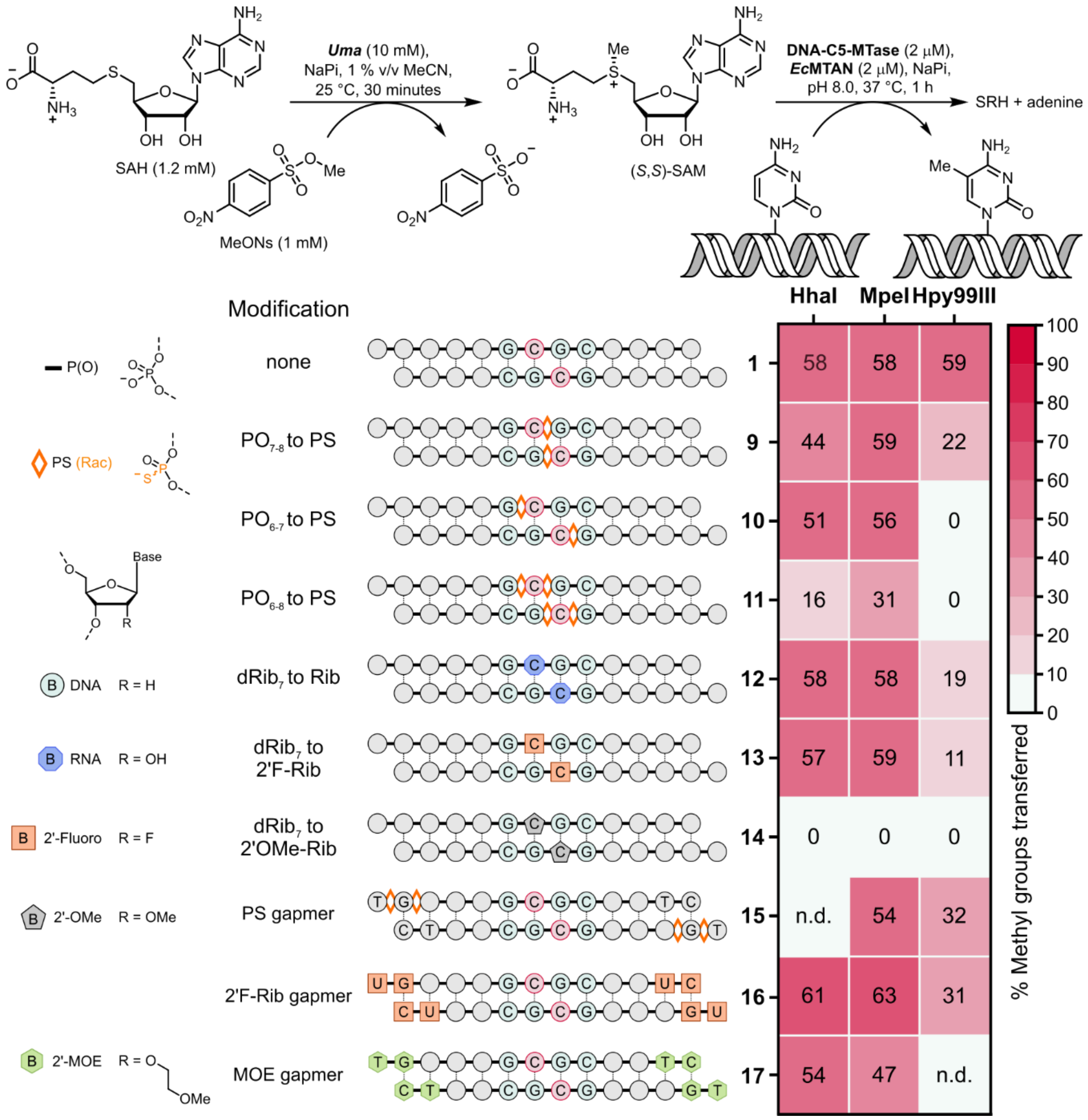

Figure S33: Heatmap of modified substrate biotransformations as a function of % of methyl groups transferred from the methyl source to oligonucleotide. These values are calculated as the amount of methylated oligonucleotide formed compared to the amount of SAM added to each reaction. For enzymatically formed SAM, this is corrected for the efficiency of SAM synthesis from MeONs (c. 95%).

Table S11: Full percent methylation data (mean of three independent repetitions ± standard deviation) used to generate the heatmap of modified substrate acceptance (main text Figure 3).

Substrate	% methylation		
	**M.HhaI**	**M.MpeI**	**M.Hpy99III**
**1**	91 ± 3	92 ± 4	93 ± 7
**9**	69 ± 6	93 ± 2	34 ± 6
**10**	81 ± 9	88 ± 4	0 ± 0
**11**	25 ± 2	49 ± 5	0 ± 0
**12**	91 ± 1	91 ± 7	30 ± 4
**13**	90 ± 2	94 ± 3	17 ± 4
**14**	0 ± 0	0 ± 0	0 ± 0
**15**	n.d.	85 ± 1	50 ± 10
**16**	97 ± 2	99 ± 1	49 ± 7
**17**	85 ± 4	75 ± 2	n.d.

n.d. – not determined

Methylation of Aganirsen analogues

A representative ASO therapeutic, Aganirsen was selected based on the presence of multiple DNA-C5-MTase recognition sites — namely, two MpeI (**C**G) sites, one HpaII (C**C**GG) and four CviPI sites (G**C**). The all-phosphodiester compound was synthesised for examination of methylation efficiency and regioselectivity by MpeI.

Reaction conditions were as described above except the concentration of MpeI was increased to 4 µM. Methylation of the all-phosphodiester analogue of aganirsen (**18**) proceeded to 91 ± 0.5% efficiency (single methylation only) without a second exogenous complementary strand. Two plausible methylation sites for MpeI exist in this compound and so the premethylated analogues **18a** and **18b** were used to establish the methylation selectivity. Oligonucleotide **18a** was not efficiently turned over, with background methylation of 2 ± 1% observed. On the other hand, substrate **18b** was methylated with similar efficiency to the parent strand **18** (88 ± 5%) to produce the doubly methylated analogue of Aganirsen (one methyl group installed during oligo synthesis and one enzymatically, see HRMS data below). This confirms the site selective methylation of the site closest to the 5' end of the substrate. Upon further inspection, a 6-nucleotide palindromic sequence is observed centred around this CG site. Plausibly, the formation of a 6-base pair duplex from this motif enables the selective binding and methylation of this site by MpeI.

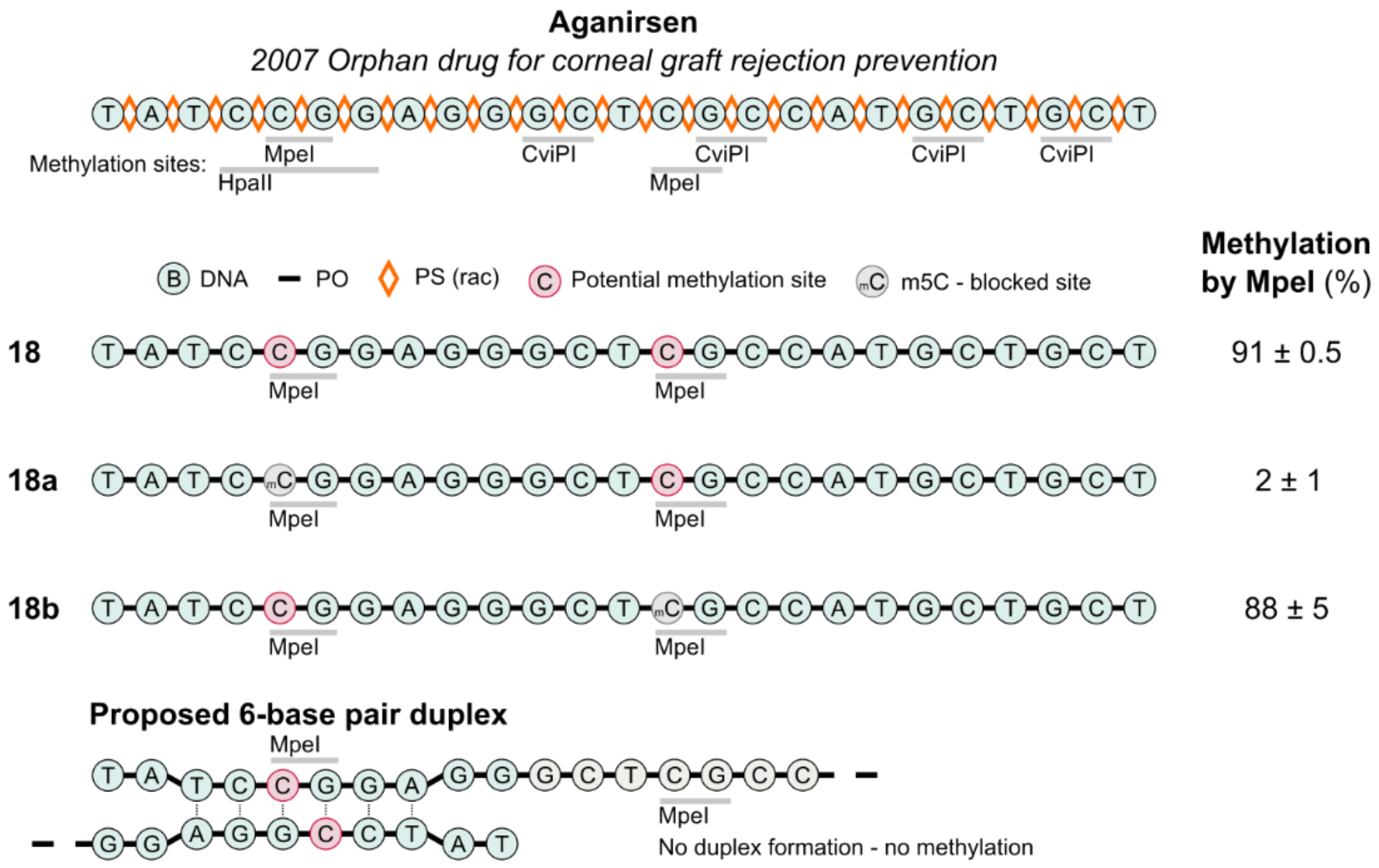

Figure S34: Methylation of Aganirsen analogues by MpeI.

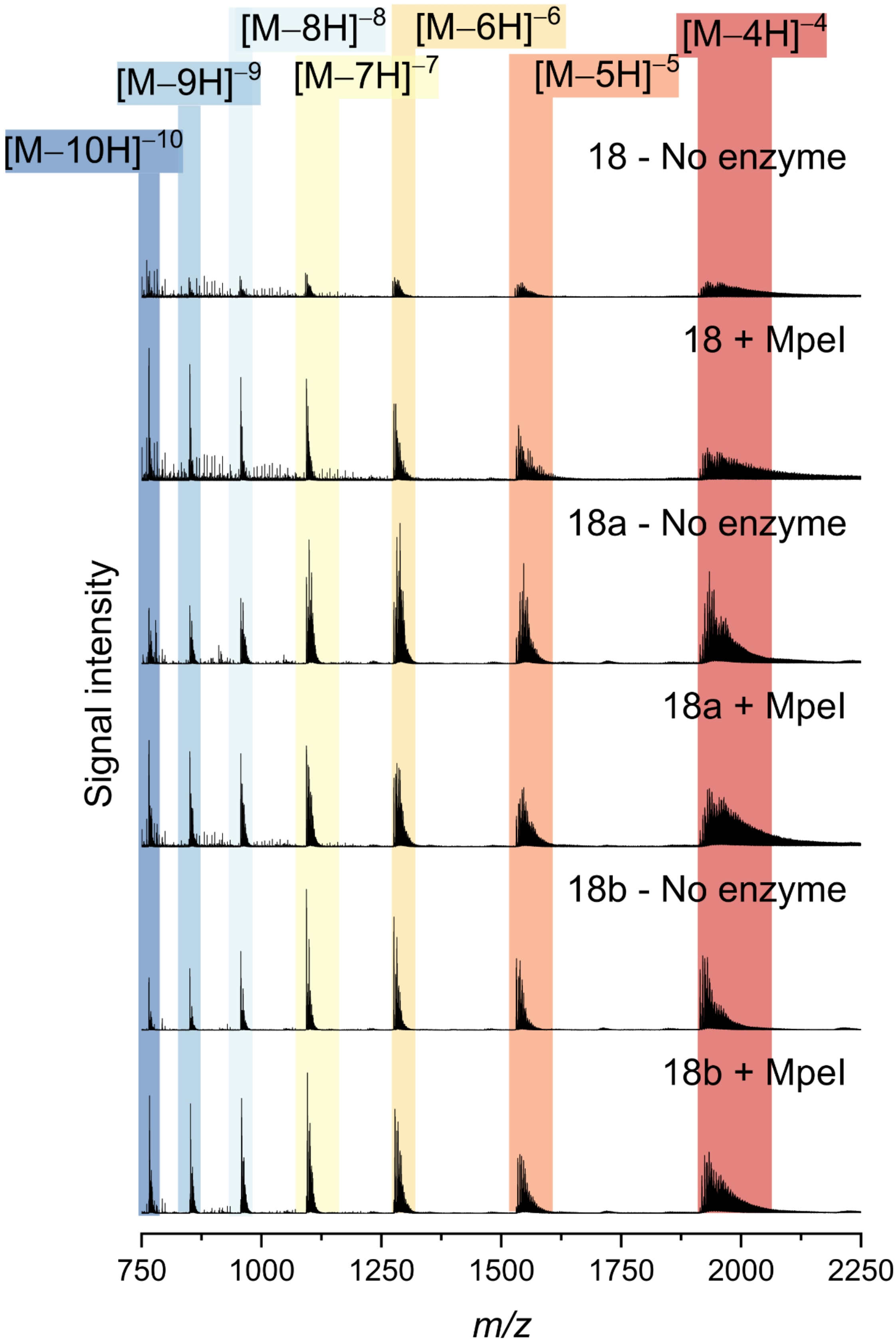

Figure S35: Stacked raw HR-ESI-MS spectra from biotransformations and no enzyme control reactions with substrates **18**, **18a** and **18b** showing the full spectral region and charge states.

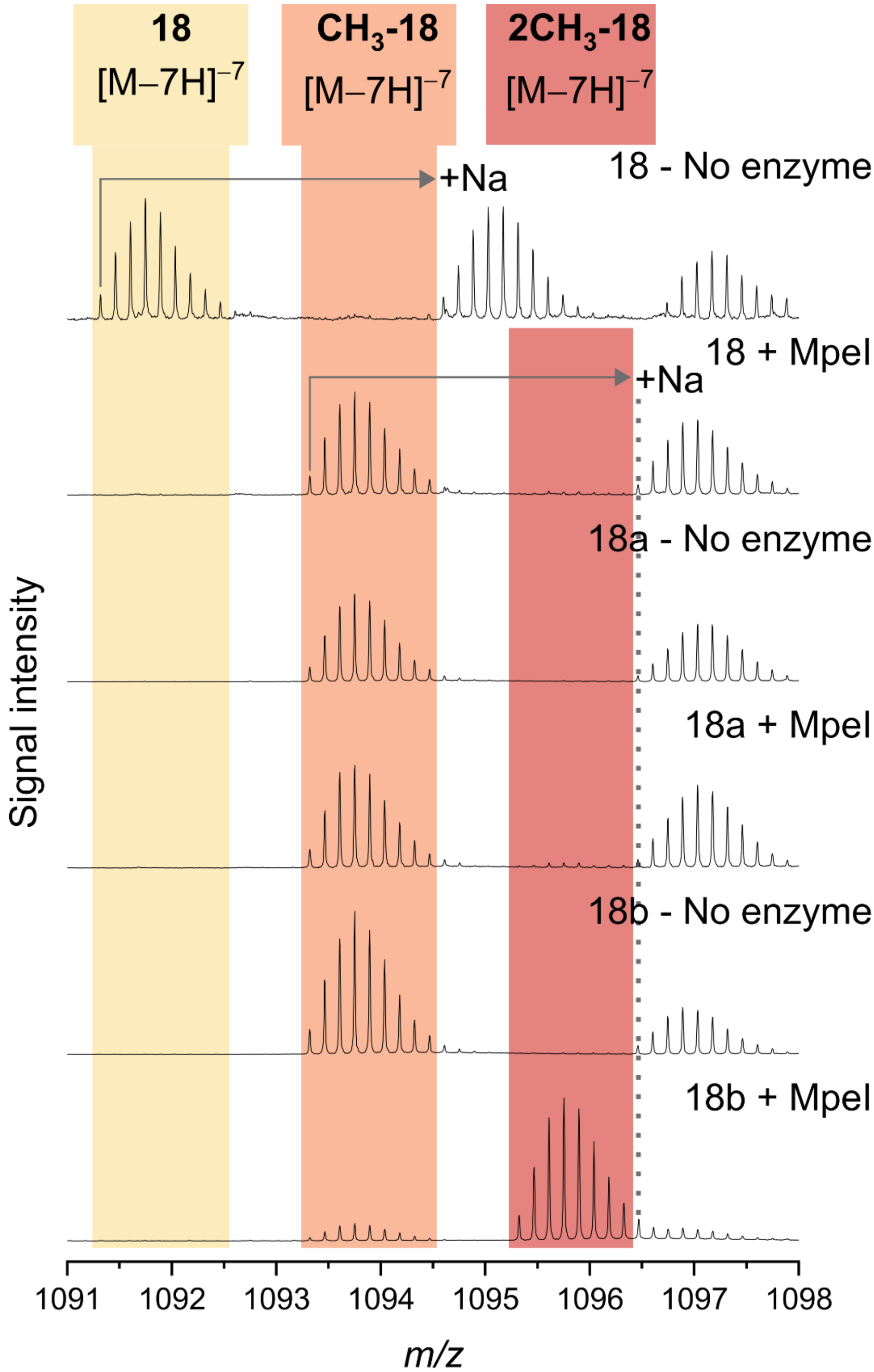

Figure S36: Stacked raw HR-ESI-MS spectra from biotransformations and no enzyme control reactions with substrates **18**, **18a** and **18b** showing the −7 charge states.

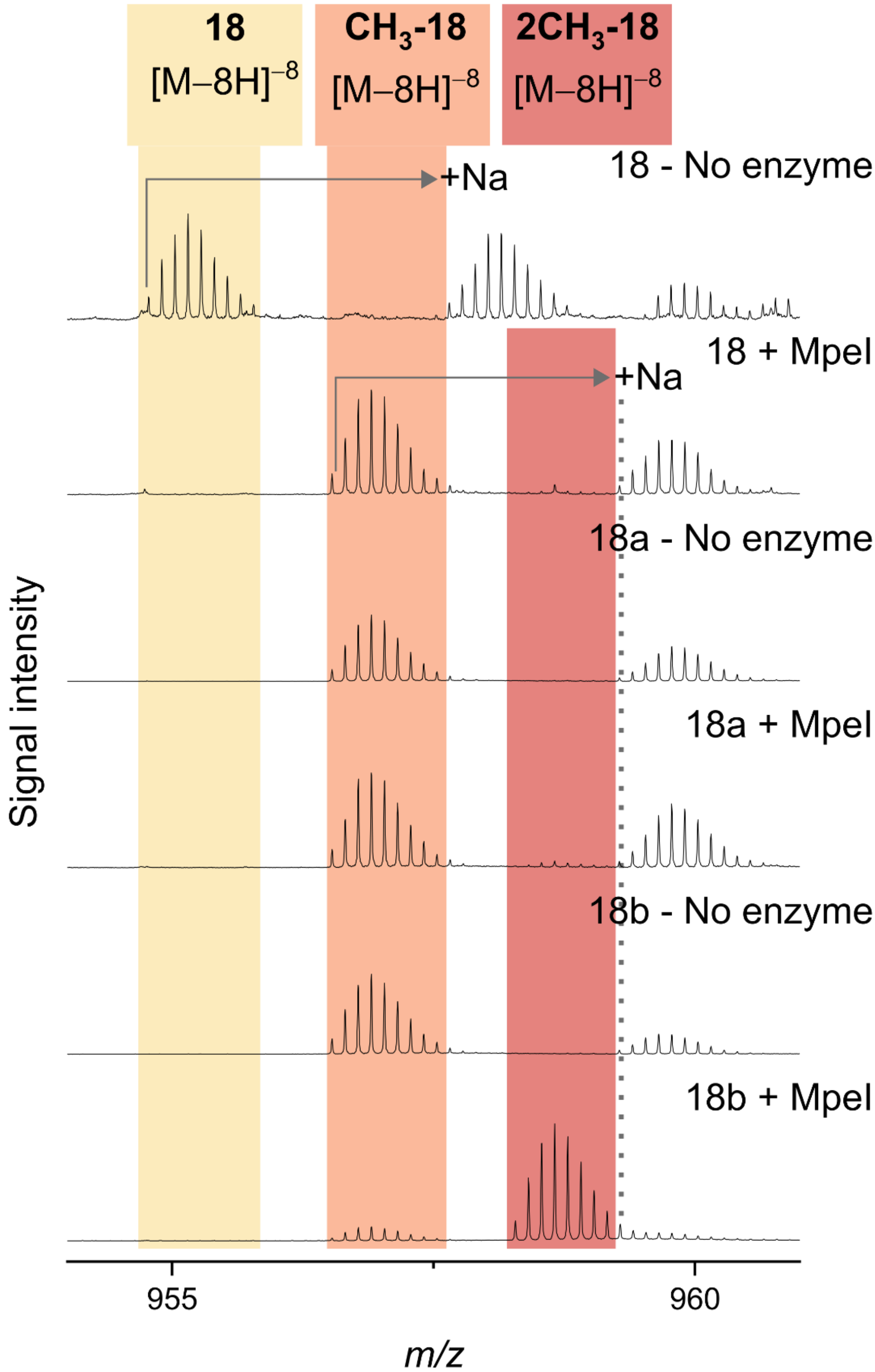

Figure 37: Stacked raw HR-ESI-MS spectra from biotransformations and no enzyme control reactions with substrates **18**, **18a** and **18b** showing the −8 charge states.

Calculated monoisotopic mass **18** $[M{-}8H]^{-8}$: 954.7783, observed: 954.777 (error 1.3 ppm). Calculated monoisotopic mass **CH_3-18** $[M{-}8H]^{-8}$: 956.5296, observed: 956.530 (error <1 ppm). Calculated monoisotopic mass **$2CH_3$-18b** $[M{-}8H]^{-8}$: 958.2822, observed: 958.283 (error <1 ppm).

Scale up biotransformations

Biotransformations at 2 mg scale with stable isotope precursors were performed as follows:

SAM formation. A 1.5 mL reaction was performed in a 15 mL falcon tube and contained: *Uma*HMT (10 µM), SAH (1.2 mM, from a 5 mM stock in 2.5% v/v DMSO $_{(aq.)}$), MeONs (natural isotope abundance or d_3-MeONs, 1 mM, from a 100 mM stock in MeCN) and NaPi buffer (50 mM, pH 8.0). The reaction was incubated horizontally in a shaking incubator at 25 °C for 30 minutes and the SAM concentration quantified by IEX-HPLC. No work up or purification was performed, the reaction mixture was used directly as a stock solution for the DNA methylation reaction.

DNA methylation. A 10 mL reaction was performed in a 50 mL falcon tube and contained; M.MpeI (2 µM), *Ec*MTAN (2 µM), SAM (75 µM, as a crude preparation from the HMT reaction, 1.5 eq.), oligonucleotide substrate GCGC-13mer (50 µM single stranded basis, 0.197 mg mL^{-1}, 1.97 mg), DTT (0.5 mM) and NaPi buffer (50 mM, pH 8.0). The reaction was incubated horizontally in a shaking incubator at 37 °C for 1 hour after which time protein aggregation was observed. The reaction was analysed by HRMS following DNA precipitation (analytical procedure as described) and the conversion to methylated oligonucleotide was found to be quantitative. The reaction mixture was centrifuged (5251 rcf, 15 minutes) and the supernatant decanted for dialysis against MilliQ water (Spectra/Por®3 dialysis membrane, 3.5 kDa MWCO, 18 mm width) thrice.

Natural isotope abundance MeONs: The product was isolated as 1.74 mg of a white solid (88% yield). HRMS (ESI) calculated 3961.7135 (for $C_{128}H_{163}N_{47}O_{77}P_{12}$); found 3961.74 after deconvolution (M, 6.7 ppm error).

***d*₃-MeONs**: The product was isolated as 1.15 mg of a white solid (58% yield). HRMS (ESI) calculated 3964.7324 (for $C_{128}H_{160}D_3N_{47}O_{77}P_{12}$); found 3964.75 after deconvolution (M, 4.4 ppm error).

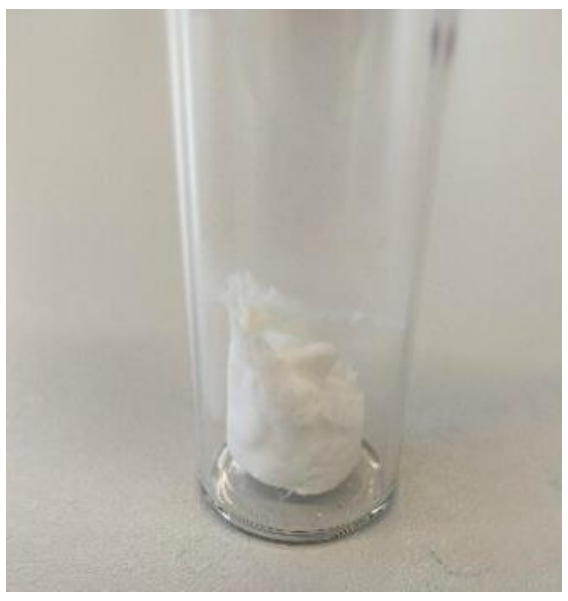

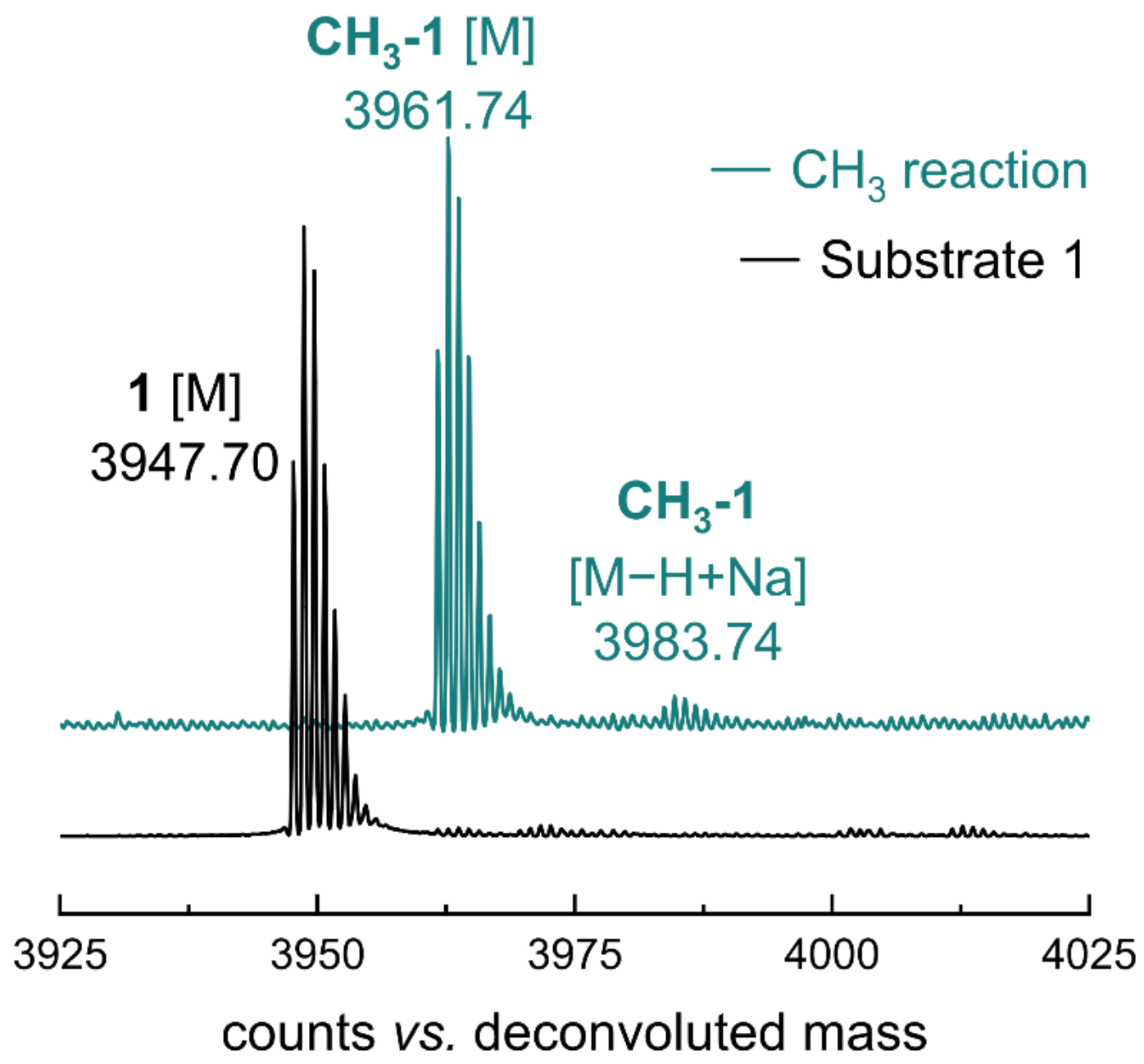

Figure S38: Deconvoluted HR-ESI-MS data for the product of a 10 mL scale up biotransformation of **1** with natural isotope abundance MeONs (blue, top) overlaid with substrate **1** spectra. No remaining substrate (**1**) was detected by HRMS after 1h of reaction time. Marked masses correspond to the monoisotopic neutral mass of each species detected after deconvolution.

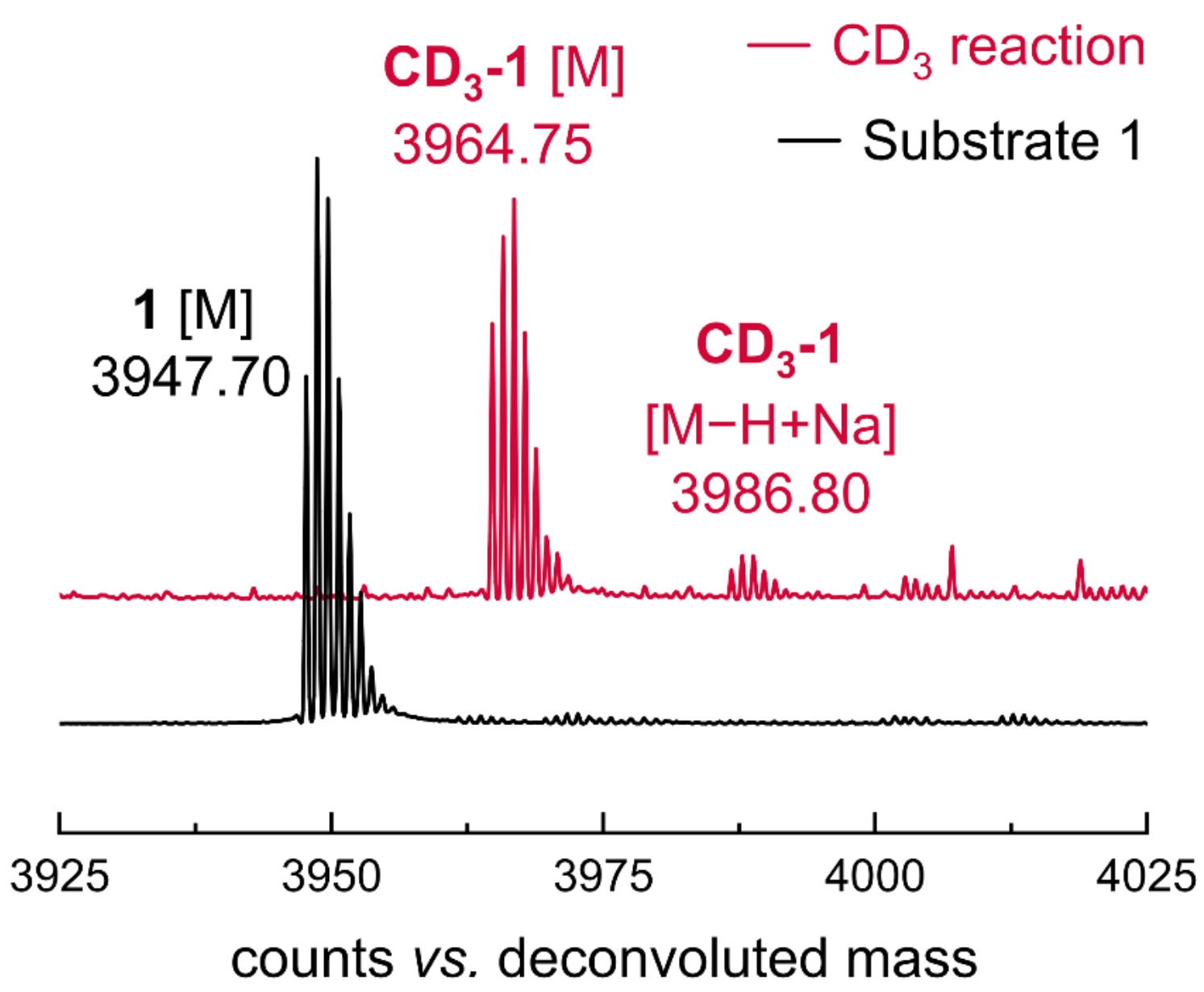

Figure S39: Deconvoluted HR-ESI-MS data for the product of a 10 mL scale up biotransformation of **1** with d_3-MeONs (red, top) overlaid with substrate **1** spectra. No remaining substrate (**1**) was detected by HRMS after 1h of reaction time. Marked masses correspond to the monoisotopic neutral mass of each species detected after deconvolution.

Method for enzymatic radiolabelling of oligonucleotides

SAM formation. [^{3}H]methyl nosylate (1.85 TBq; 50 mCi) was purchased from RC Tritec AG as a solution in toluene (approx. concentration 185 – 370 GBq mL^{-1} (5 – 10 mCi mL^{-1}) with molar radioactivity of 3 TBq mmol^{-1} (81 Ci mmol^{-1}). The toluene was removed *in vacuo* and the resulting solid resuspended in acetonitrile, transferred to a 5 mL Eppendorf tube and the acetonitrile removed under an argon stream. DMSO (6.25 µL) was added to the residue, and the reaction was prepared with a total volume of 625 µL and the following concentrations of each component (diluted from stock solutions): ***Uma*HMT** (10 µM), SAH (1.2 mM, from a 5 mM stock in 2.5% v/v DMSO$_{(aq.)}$) and NaPi buffer (50 mM, pH 8.0). The final concentration of [^{3}H]MeONs in the reaction was 1 mM. The reactions were incubated in a thermomixer at 25 °C for 30 minutes, then used directly as a stock solution for the subsequent DNA methylation reaction without work up or purification.

DNA methylation. 100 µL reactions were performed in 500 µL Eppendorf tubes and contained; **DNA-C5-MTase** (2 µM), ***Ec*MTAN** (2 µM), [^{3}H]SAM (75 µM, as a crude preparation from the HMT reaction, 1.5 eq.), oligonucleotide substrate (50 µM single stranded basis, c. 20 µg for a 4000 g mol^{-1} oligonucleotide), DTT (1 mM) and NaPi buffer (50 mM, pH 8.0). The reactions were incubated in a thermomixer at 37 °C for 1 hour. The reaction was analysed by Radio-HPLC as described in "Materials and methods".

Table S12: Summary of oligonucleotide radiolabelling efficiency.

Substrate	Enzyme	DoL / %	MA / Ci mmol^{-1}	^{3}H-Oligo:SAM / %
**1**	M.HhaI	69	55.3	69
**2**	M.HaeIII	>99	>80	75
**6**	M.HaeIII	69	55.3	71
**1**	M.MpeI	>99	>80	72
**3**	M.MpeI	97	77.7	77
**4**	M.MpeI	76	60.4	72
**3**	M.HpaII	85	67.9	74
**6**	EcDCM	28	22.3	35

DoL (degree of labelling) is calculated as the % of molar activity detected on the final oligonucleotide compared to molar activity of the [^{3}H]MeONs reagent (c. 3 TBq mmol^{-1}; 81 Ci mmol^{-1}). MA is the molar activity of the oligonucleotide product calculated per labelled oligonucleotide strand. ^{3}H-Oligo:SAM (%) is calculated by integration of Radio-HPLC traces (below).

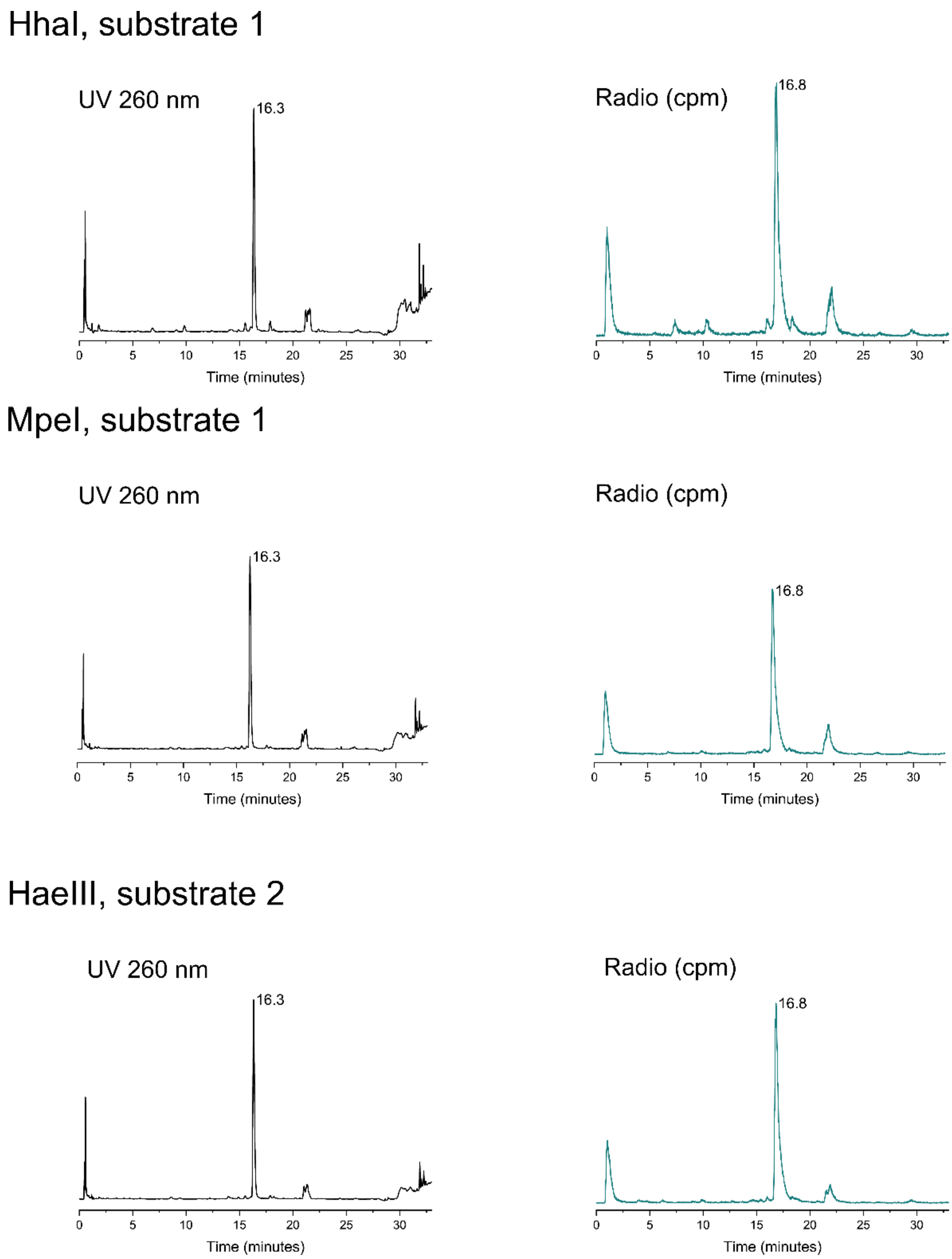

Figure S40: Exemplary HPLC traces from enzymatic radiolabelling experiments.

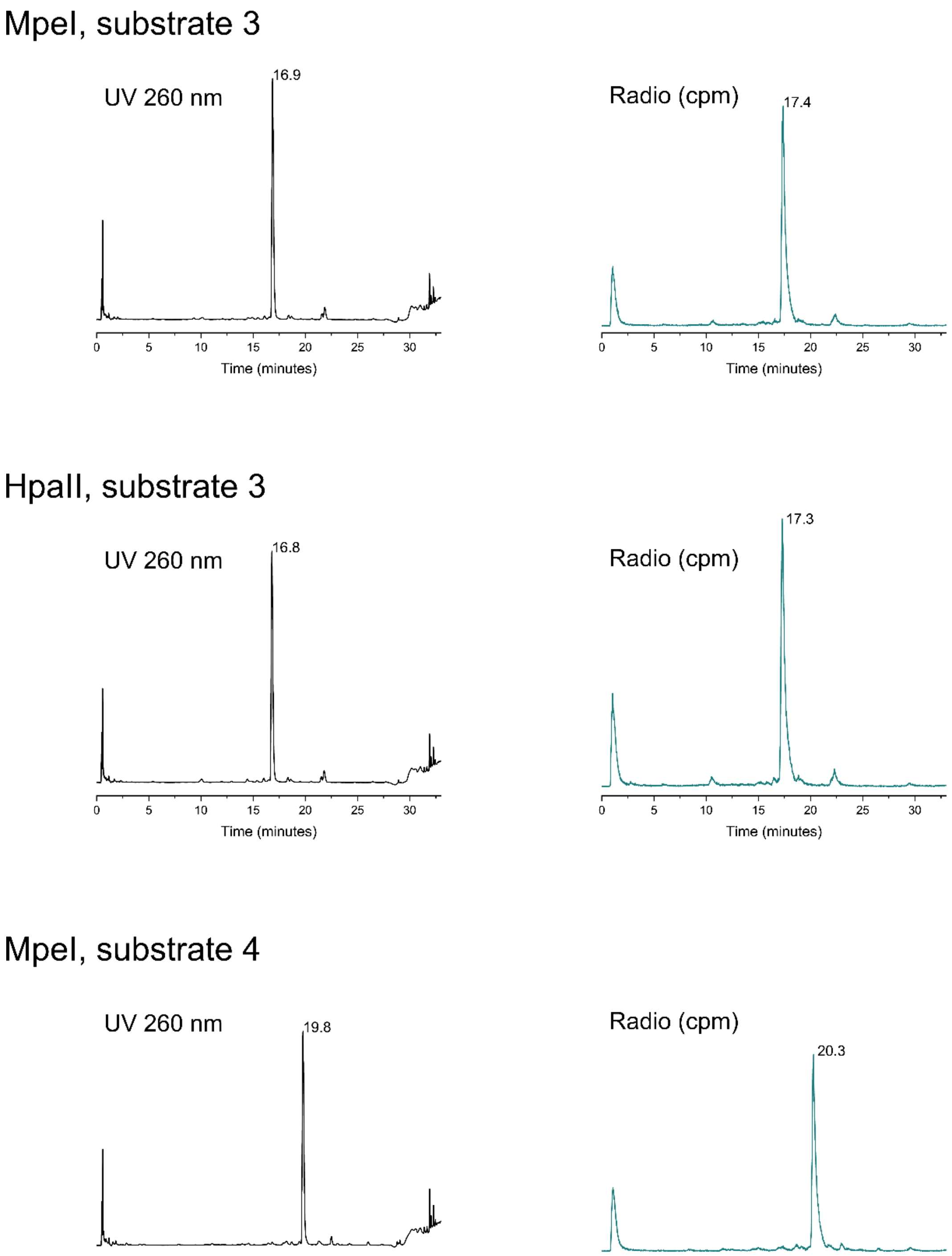

Figure S41: Exemplary HPLC traces from enzymatic radiolabelling experiments.

HaeIII, substrate 6

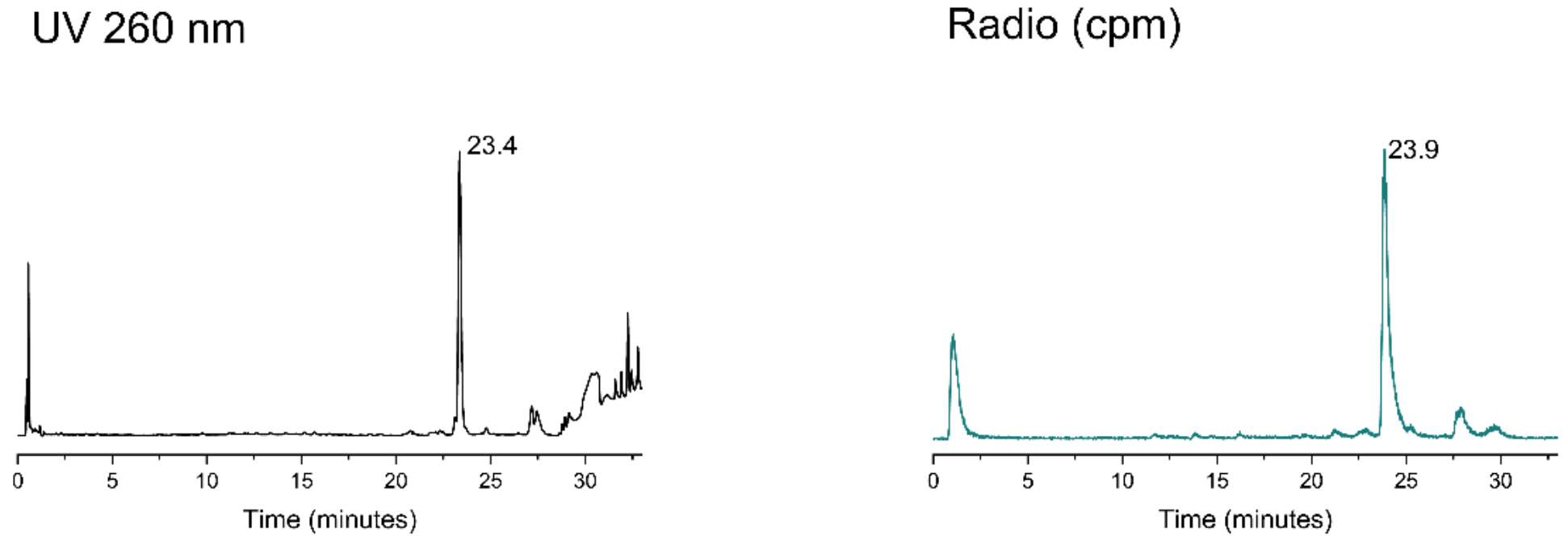

EcDCM, substrate 6

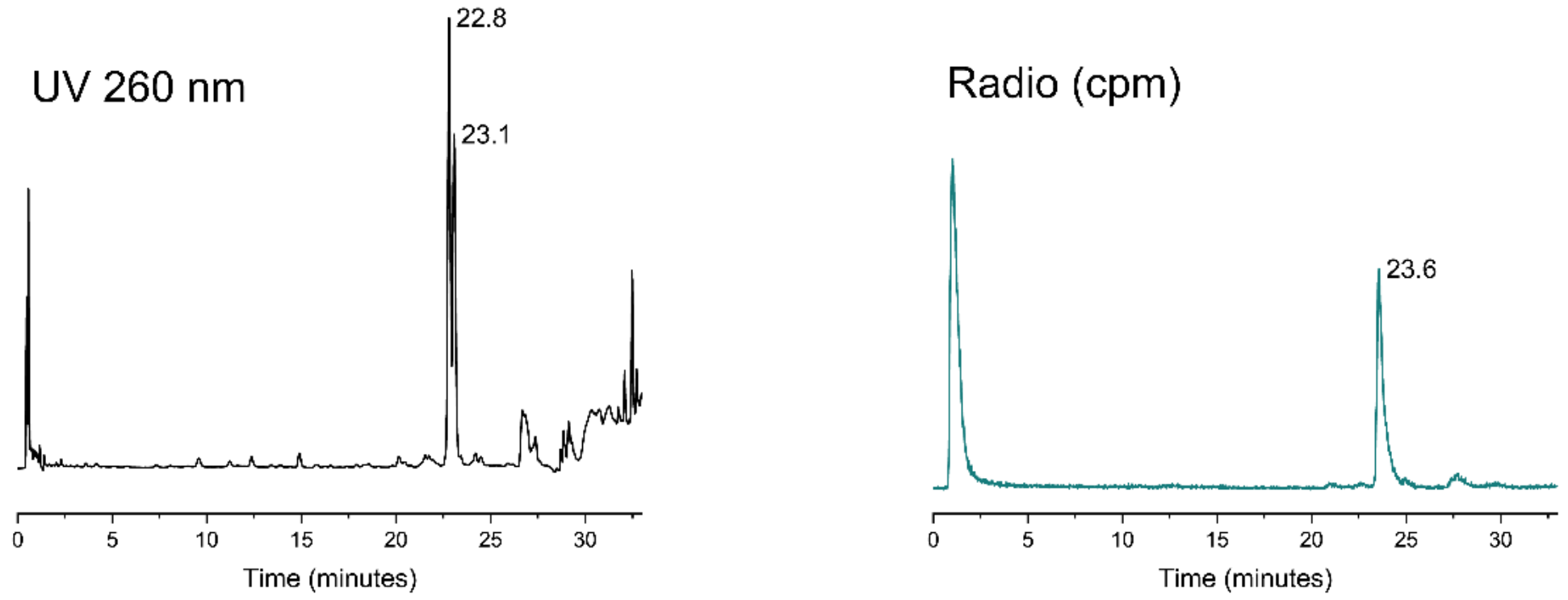

No enzyme, substrate 6

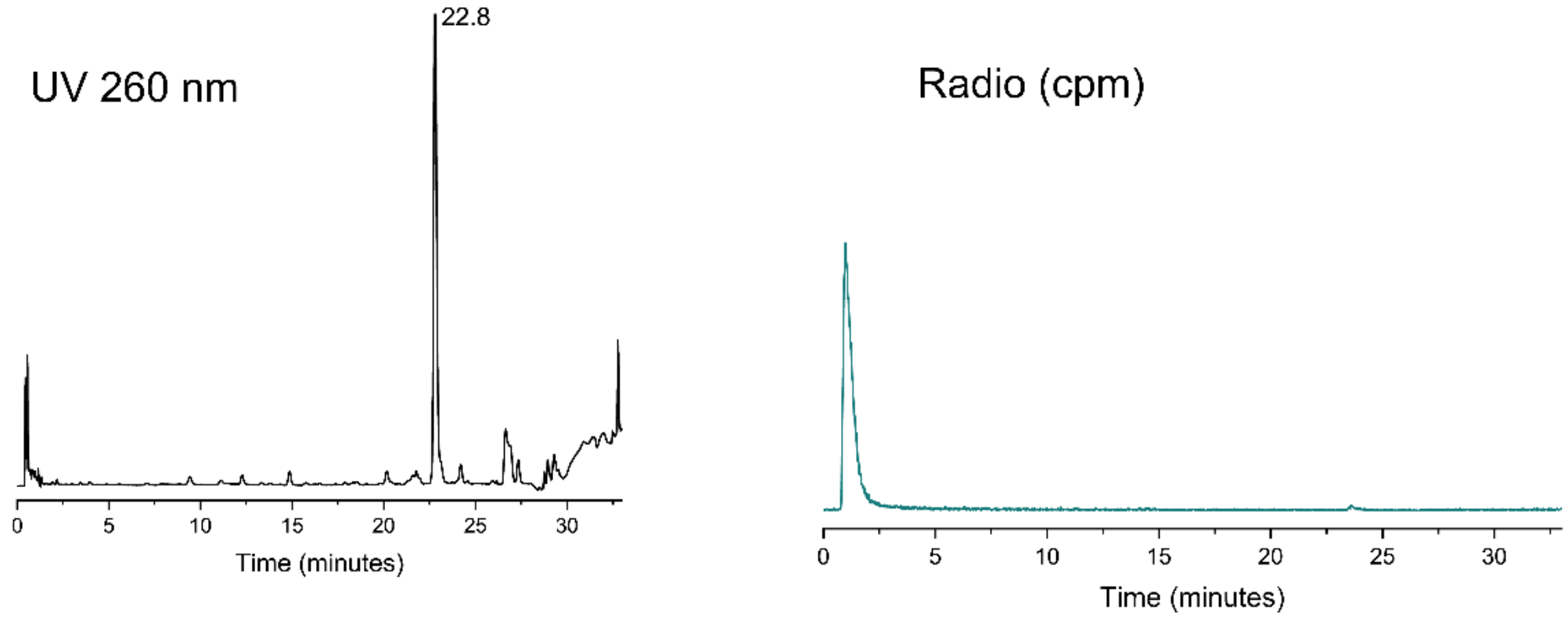

Figure S42: Exemplary HPLC traces from enzymatic radiolabelling experiments.

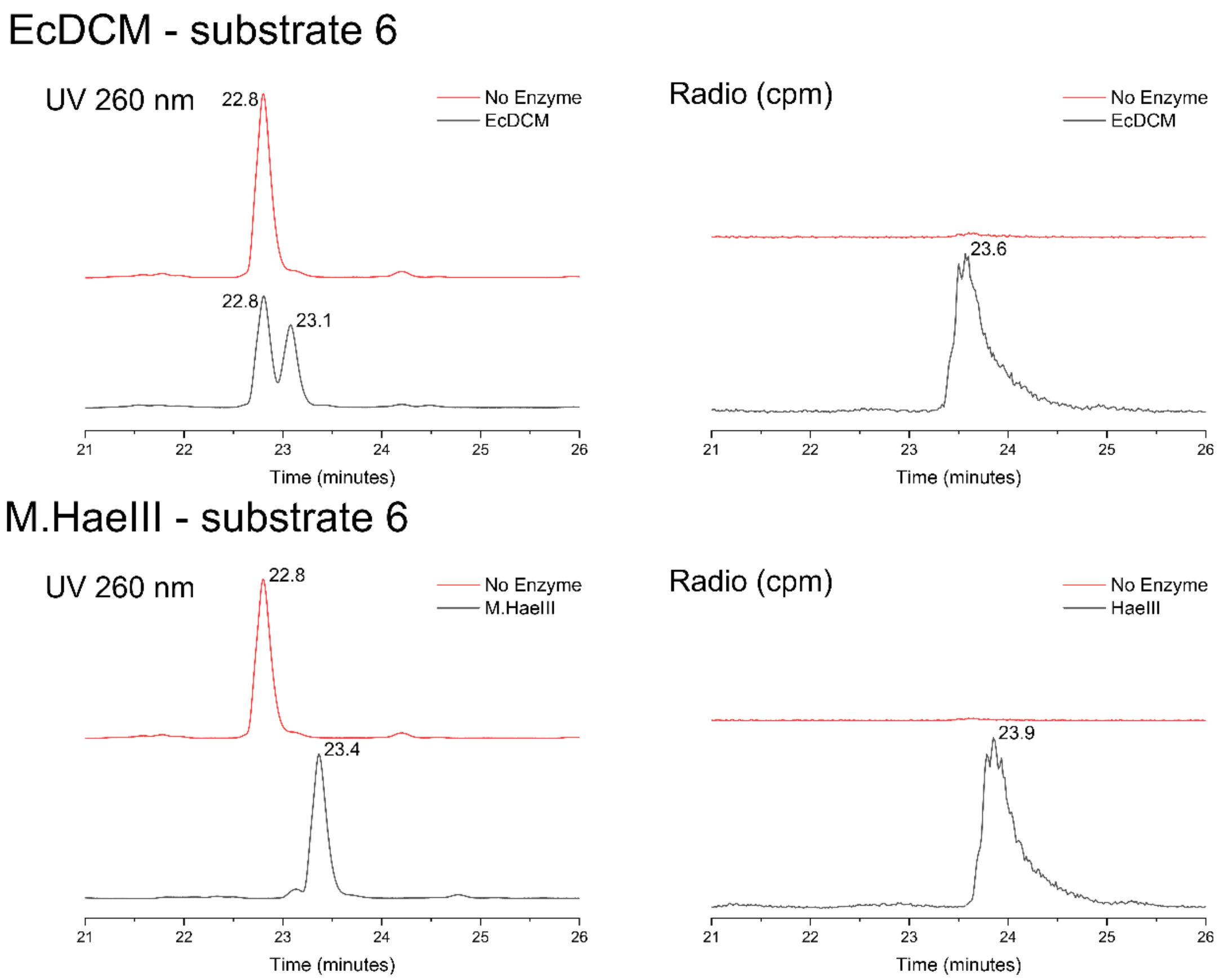

Figure S43: Stacked HPLC-UV and Radio-HPLC traces of substrate 6 with and without DNA-MTase EcDCM (top) or HaeIII (bottom). Emergence of a new peak in the UV trace with increased retention time and a corresponding radioactive peak in the radioactivity trace indicates that tritium-methylation of the substrate has occurred.

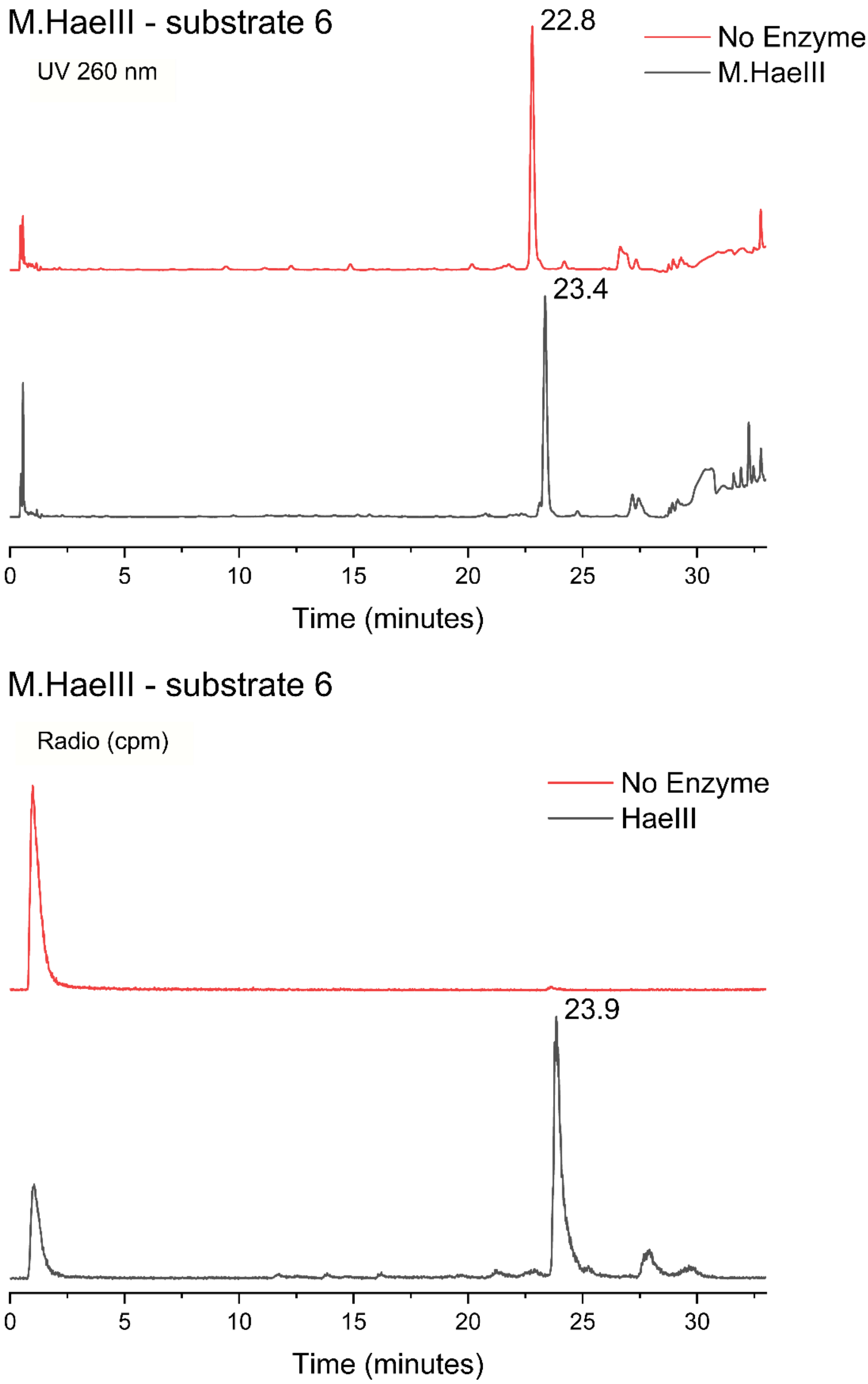

Figure S44: Stacked HPLC-UV (top) and Radio-HPLC (bottom) traces of substrate 6 with and without HaeIII (full spectral region).

Representative protocol for enzymatic radiolabelling of an antisense oligonucleotide

Oligonucleotide preparation. DNA-MTases require a duplex DNA substrate for substrate recognition. Accordingly, for labelling of a single stranded ASO, the following two criteria must be considered.

1. Identify an appropriate DNA-MTase with a recognition site that is present in your oligonucleotide of interest.
    a. Several DNA methyltransferases are commercially available from NEB (e.g. 'CpG Methyltransferase M.SssI': NEB catalog no. M0226S and 'GpC Methyltransferase M.CviPI': NEB catalog no. M0227S and others).
    b. Alternatively, appropriate enzyme candidates can be searched from enzyme databases including REBASE, where DNA-MTases with characterised methylation sites have been deposited (>110 unique sites as of 2026.05.18).
2. The activity of the DNA-MTase should be assessed against the oligonucleotide substrate with and without annealing of a short complementary templating strand.
    a. We observe that some antisense oligonucleotides (e.g. aganirsen) can be efficiently methylated without addition of a templating strand, presumably due to a transient duplex formation of a short self-complementary sequence under reaction conditions.

If the activity should be dependent on the presence of a second templating strand, we suggest use of a c. 8mer complement which can be synthesised with a m5C preinstalled at the methylation site, ensuring that only the target therapeutic oligonucleotide strand is methylated with the radiolabel.

To anneal the complementary DNA strands the following protocol can be applied: Prepare solutions of each oligonucleotide at 2x the desired concentration of duplex oligonucleotide stock solution (e.g. for MTase reactions prepared with a 200 µM oligonucleotide stock solution, prepare solutions of each oligonucleotide at 400 µM) in a suitable buffer (e.g. 10 mM NaPi or Tris-HCl with 50 mM NaCl and optionally 1 mM EDTA, pH 7.5 – 8.0). Mix the two oligonucleotide solutions in a 1:1 ratio and heat at 95°C for 5 minutes and then allow to cool to room temperature slowly (c. 30 – 60 minutes). The duplex oligonucleotide solution is now ready to use. If desired, the duplex formation can be confirmed with native PAGE.

SAM formation. [^{3}H]methyl nosylate (1.85 TBq; 50 mCi) was purchased from RC Tritec AG as a solution in toluene (approx. concentration 185 – 370 GBq mL^{-1} (5 – 10 mCi mL^{-1}) with molar radioactivity of 3 TBq mmol^{-1} (81 Ci mmol^{-1}). The toluene was removed *in vacuo* and the resulting solid resuspended in acetonitrile, transferred to a 5 mL Eppendorf tube and the acetonitrile removed under an argon stream. DMSO (6.25 µL) was added to the residue, and the reaction was prepared with a total volume of 625 µL and the following concentrations of each component (diluted from stock solutions): ***Uma*HMT** (10 µM), SAH (1.2 mM, from a 5 mM stock in 2.5% v/v DMSO $_{(aq.)}$) and NaPi buffer (50 mM, pH 8.0). The final concentration of [^{3}H]MeONs in the reaction was 1 mM. The reactions were incubated in a thermomixer at 25 °C for 30 minutes, then used directly as a stock solution for the subsequent DNA methylation reaction without work up or purification.

DNA methylation. Reactions were performed on 100 µL to 10 mL scale and contained; **DNA-C5-MTase** (2 µM), ***Ec*MTAN** (2 µM), [^{3}H]SAM (75 µM, as a crude preparation from the HMT reaction, 1.5 eq.), oligonucleotide substrate (50 µM single stranded basis), DTT (1 mM) and NaPi buffer (50 mM, pH 8.0). The reactions were incubated in a thermomixer at 37 °C for 1 hour. The reaction was analysed by Radio-HPLC as described in "Materials and methods". Where high methylation efficiency is obtained, the oligonucleotide sample can be purified from protein, small molecules and buffer salts as follows. Proteins were precipitated by heat treatment at 95 °C for at least 10 minutes followed by centrifugation (21,300 x g, 5 minutes). The supernatant was decanted and filtered through a centrifugal filtration device (Amicon, 3K MWCO) and washed with water thrice. Alternatively, the

supernatant can be desalted by iPrOH/EtOH precipitation as described in “Materials and methods”. The purified oligonucleotide was analysed by HPLC-UV/Radio and liquid scintillation counting.

As an instructive example, the sequences of FDA approved ASOs and a comparison with known DNA-C5-MTase recognition sites is given in Table S13, below. Methylation sites are given including the identity of the cytosine or 5-methylcytosine (here, mC) in the oligonucleotide. Where ‘mC’ is marked, radiomethylation of the unmethylated oligonucleotide analogue would give an identical sequence as the drug substance, whereas where the site is marked with ‘C’ the radiomethylated substance would incorporate a mC nucleobase which is not present in the true drug substance.

Table S13: Overview of FDA approved antisense oligonucleotide therapies (excluding PMO and siRNAs), their nucleobase sequences and overlap with known DNA-C5-MTase methylation sites.

Name	Year	Nucleobase sequence (5' - 3')	Me site 1	Me site 2
Fomivirsen	1998	GCGTTTGCTCTTCTTCTTGCG	GC (M.CviPI)	-
Mipomersen	2013	**GmCmCUC**AGUCUGmCUUmC**GmCAmCC**	GmC (M.CviPI)	CUG (M1I880)
Nusinersen	2016	**TmCAmCTTTmCATAATGmCTGG**	**GmC** (M.CviPI)	-
Inotersen	2016	**TmCTTG**GTTACATGAA**ATmCmCmC**	CATG (M.FatI)	-
Volanesorsen	2019	**AGmCTT**mCTTGTmCmCAGmC**TTTAT**	mCmC (M.CviPII)	mCAG (M1I880)
Eplontersen	2023	**TmCTTG**GTTACATGAA**ATmCmCmC***	CATG (M.FatI)	-
Tofersen	2023	**mCAGGA**TAmCATTTmCTA**mCAGmCT**	-	-
Olezarsen	2024	***AGmCTT**mCTTGTmCmCAGmC**TTTAT**	mCmC (M.CviPII)	mCAG (M1I880)
Donidalorsen	2025	***TGmCAA**GmCTmCTTGGmC**AAAmCA**	GmC (M.CviPI)	-

Nucleobases marked in bold indicate a 2'-MOE nucleotide and those not in bold indicate a 2’deoxy nucleotide. *indicates a triGalNAc conjugation. mC = 5-methyl cytosine. Enyzme names are given where they have been assigned in the literature (e.g. M.CviPI) or, where no name has been assigned, the UniProt ID is given (e.g. M1I880)

References

(1) Lamiable-Oulaidi, F.; Harijan, R. K.; Shaffer, K. J.; Crump, D. R.; Sun, Y.; Du, Q.; Gulab, S. A.; Khan, A. A.; Luxenburger, A.; Woolhouse, A. D.; Sidoli, S.; Tyler, P. C.; Schramm, V. L. Synthesis and Characterization of Transition-State Analogue Inhibitors against Human DNA Methyltransferase 1. *J. Med. Chem.* **2022**, *65* (7), 5462–5494. https://doi.org/10.1021/acs.jmedchem.1c01869.

(2) Liao, C.; Seebeck, F. P. S-Adenosylhomocysteine as a Methyl Transfer Catalyst in Biocatalytic Methylation Reactions. *Nat Catal* **2019**, *2* (8), 696–701. https://doi.org/10.1038/s41929-019-0300-0.

(3) Daujotytė, D.; Vilkaitis, G.; Manelytė, L.; Skalicky, J.; Szyperski, T.; Klimašauskas, S. Solubility Engineering of the HhaI Methyltransferase. *Protein Engineering, Design and Selection* **2003**, *16* (4), 295–301. https://doi.org/10.1093/proeng/gzg034.
